# A Study of an 8-Aminoquinoline-Directed C(sp^2^)–H Arylation Reaction on the Route to Chiral Cyclobutane Keto Acids from Myrtenal

**DOI:** 10.1021/acs.joc.1c00774

**Published:** 2021-05-27

**Authors:** Monireh Pourghasemi Lati, Jonas Ståhle, Michael Meyer, Oscar Verho

**Affiliations:** † Department of Organic Chemistry, Arrhenius Laboratory, Stockholm University, SE-106 91 Stockholm, Sweden; ‡ Department of Medicinal Chemistry, Uppsala Biomedical Centre, Uppsala University, SE-751 23 Uppsala, Sweden

## Abstract

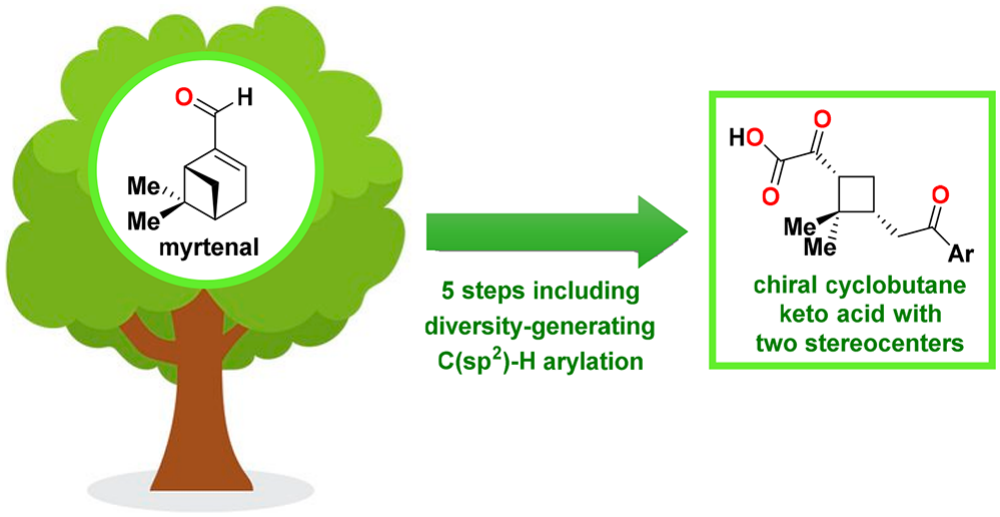

This work outlines a synthetic route that can be used to access chiral cyclobutane keto acids with two stereocenters in five steps from the inexpensive terpene myrtenal. Furthermore, the developed route includes an 8-aminoquinoline-directed C(sp^2^)–H arylation as one of its key steps, which allows a wide range of aryl and heteroaryl groups to be incorporated into the bicyclic myrtenal scaffold prior to the ozonolysis-based ring-opening step that furnishes the target cyclobutane keto acids. This synthetic route is expected to find many applications connected to the synthesis of natural product-like compounds and small molecule libraries.

Fossil-based feedstocks have been instrumental for the development of our modern society, and today they constitute the primary carbon source for the organic chemicals utilized by the academic and industrial research spheres.^1^ Unfortunately, the use of fossil-based feedstocks is associated with many detrimental effects on our climate, and furthermore, they are a finite resource, which means that our society will soon need to transition to other renewable alternatives. As a result, research within the field of chemistry has during the past several decades focused extensively on the development of new synthetic processes that make use of green and renewable building blocks in place of fossil-based feedstocks. Here, biomass-derived feedstocks represent a promising and sustainable carbon source that is capable of meeting the future needs of our society.^2^ Of the many compounds available in the biomass pool, terpenes constitute a particularly well-utilized class of natural products^3^ that has been extensively used in the manufacturing of chemical reagents,^4^ fragrances,^3a,5^ fuels,^6^ pharmaceuticals^3a,7^ and polymers.^8^

In parallel to research in sustainable chemistry, the field of C–H functionalization has expanded rapidly over the past decade and provided many new opportunities for synthetic chemistry.^9^ C–H functionalization chemistry holds great potential to be used for the diversification and structural elaboration of biomass-derived synthetic precursors.^10^ For example, our group recently demonstrated that 8-aminoquinoline (8-AQ)-directed C–H arylation chemistry can be used to access chiral cyclobutane derivatives with three contiguous stereocenters from the terpene verbenone.^11^ This study of ours was inspired by the work from the groups of Baran^12^ and Reismann^13^ on the 8-AQ-directed C–H functionalization of cyclobutane derivatives, which in turn was an extension of the pioneering work of Daugulis and co-workers (Scheme 1a).^14^ However, it is important to point out here that the 8-AQ-directed C–H functionalization methodology has also been applied to many other compound classes by several other research groups.^15^

**Scheme 1:**
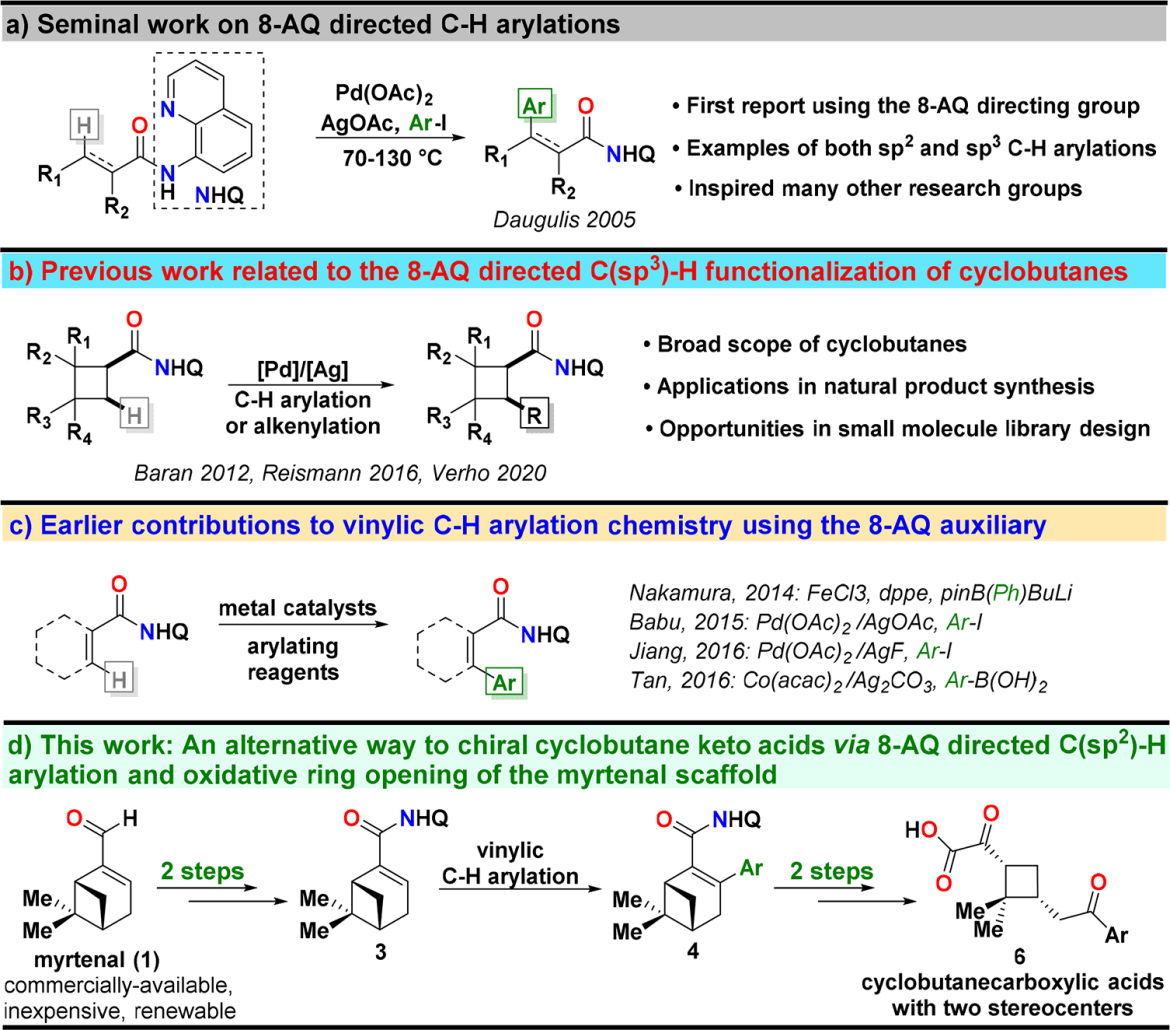
(a) Seminal Work of Daugulis and Co-workers on 8-AQ-Directed C–H Functionalization Chemistry, (b) Prior Art in 8-AQ-Directed C–H Functionalization of Cyclobutanes, (c) Previous contributions to Vinylic C–H Arylation Using the 8-AQ-Directing Group, and (d) Our Work Outlining a Novel Synthetic Route to Chiral Cyclobutane Keto Acids That Proceeds via Vinylic C–H Arylation and Oxidative Ring Opening of the Myrtenal Scaffold

As a part of our group’s ongoing efforts to establish new synthetic pathways to structurally elaborate cyclobutane derivatives from biomass-derived precursors, we became interested in the terpene myrtenal as it presented an inexpensive starting point for accessing a novel series of chiral cyclobutane keto acids with two stereocenters. Unlike previous C–H functionalization-based approaches to accessing complex cyclobutane derivatives that have involved either directed C–H alkenylation or arylation directly on the cyclobutane scaffold (Scheme 1b), we sought to explore an alternative path based on 8-AQ-assisted vinylic C–H arylation chemistry^16^ (Scheme 1c) followed by oxidative opening of the myrtenal scaffold. Our synthetic route that is outlined in Scheme 1d begins with the preparation of 8-AQ amide **3** from myrtenal (**1**) via two simple transformations, i.e., aldehyde oxidation^17^ and installation of the 8-AQ auxiliary. As will be demonstrated herein, 8-AQ amide **3** represents an excellent substrate for directed vinylic C–H arylation chemistry, and this reaction step can be utilized as a point of diversification prior to the generation of the cyclobutane keto acid core. From the C–H arylated compounds **4**, it is then possible to access the target keto acid derivatives simply by removing the 8-AQ auxiliary and carrying out an oxidative ring opening by ozonolysis.

The central C(sp^2^)–H arylation reaction was optimized with 4-iodoanisole as the arylating agent, and the results from this study are summarized in Table 1. As our first attempt, we performed the reaction with 3 equiv of 4-iodoanisole, 5 mol % Pd(OAc)_2_, and 2 equiv of AgOAc for 16 h at 110 °C, which encouragingly resulted in a 50% yield of desired product **4a** (entry 1). It is worth noting that the conversion was full under these reaction conditions, which indicated that the reaction suffered from considerable selectivity issues. In a first effort to improve this reaction, we investigated the effects of additives, as this has in previous C–H functionalization studies been shown to have a strong influence on both reaction efficiency and selectivity. Dibenzyl phosphate [(BnO)_2_PO_2_H, 0.2 equiv],^11,15g,15h,18^ a commonly used additive for C–H functionalizations, was found to have a significant negative effect on this reaction, resulting in an only 12% yield of **4a** after 16 h at 110 °C (entry 2). A low yield was also observed with 0.2 equiv of pivalic acid (PivOH, entry 3), which constitutes another popular additive for C–H functionalization reactions.^19^ To our delight, a better result was obtained with NaOAc as the additive, which was in line with the findings from our previous study on the 8-AQ-directed C(sp^2^)–H arylation of benzofuran derivatives.^15a^ For example, when the reaction was carried out with 0.2 equiv of NaOAc at 110 °C, product **4a** could be obtained in 56% yield with 90% conversion (entry 4). However, the best results with NaOAc were obtained when the reaction temperature was decreased to 100 °C and the reaction time increased to 24 h. Under these modified conditions, it was possible to achieve a higher yield of 61% with 82% conversion when using 0.2 equiv of NaOAc (entry 5), which improved further to 68% yield and 78% conversion with 1.0 equiv of NaOAc (entry 6).

**Table 1:**
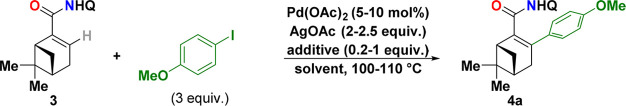
Optimization of the Pd-Catalyzed C–H Arylation of Substrate **3** with 4-Iodoanisole^a^

entry	additive (equiv)	solvent	temp (°C)	time (h)	conversion (%)^b^	yield (%)^b^
1	none	toluene	110	16	>95	50
2	(BnO)_2_PO_2_H (0.2)	toluene	110	16	37	12
3	PivOH (0.2)	toluene	110	16	79	44
4	NaOAc (0.2)	toluene	110	16	90	56
5	NaOAc (0.2)	toluene	100	24	82	61
6	NaOAc (1.0)	toluene	100	24	78	68
7	NaOAc (1.0)	HFIP	100	24	77	44
8	NaOAc (1.0)	MeCN	100	24	52	28
9	NaOAc (1.0)	DCE	100	24	81	75
10	NaOAc (1.0)	CPME	100	24	81	61
11	NaOAc (1.0)	2-MTHF	100	24	>95	63
12	NaOAc (1.0)	*t*-amyl-OH	100	24	>95	71
13^c^	NaOAc (1.0)	DCE	100	24	>95	67
14^d^	NaOAc (1.0)	DCE	100	24	>95	72
15^e^	NaOAc (1.0)	DCE	100	24	>95	58
16^f^	NaOAc (1.0)	*t*-amyl-OH	100	24	>95	73

a Reagents and conditions: substrate **3** (0.15 mmol, Q = 8-quinolinyl), 4-iodoanisole (3 equiv), Pd(OAc)_2_ (5 mol %), AgOAc (2.0 equiv), and the additive(s) were dissolved in solvent (0.5 M) and heated at the given temperature under an inert atmosphere.

b Conversions and yields were determined by ^1^H NMR against 1,3,5-trimethoxybenzene as the internal standard.

c With 2.5 equiv of AgOAc.

d With 10 mol % Pd(OAc)_2_.

e The reaction concentration was 1 M.

f Reaction performed on a 2.5 mmol scale.

After having evaluated different additives, we continued the optimization study with a solvent screen. Here, we found that the use of HFIP and MeCN resulted in significantly lower yields of product **4a** (44% and 28%, entries 7 and 8, respectively). Markedly better results were obtained with DCE that gave a 75% yield of **4a** with 81% conversion (entry 9). However, it should be pointed out that DCE is subject to regulatory controls in the European Union, and its use in commercial processes is expected to become limited worldwide soon.^20^ Gratifyingly, this C(sp^2^)–H arylation reaction also works well in a set of more process-friendly solvents (entries 10–12), such as cyclopentyl methyl ether (CPME), 2-methyltetrahydrofuran (2-MTHF), and *tert*-amyl alcohol (*t*-amyl-OH). Of these three solvents, *t*-amyl-OH gave the best results (71% yield with >99% conversion, entry 12). In attempts to further improve this C(sp^2^)–H arylation reaction, we increased the loadings of Pd(OAc)_2_ and AgOAc, as well as the reaction concentration, but unfortunately, neither of these alterations had any beneficial effect on the yield of **4a**.

On the basis of the results from the optimization study with 4-iodoanisole, we decided to use the following reaction conditions for the survey of aryl iodide scope: 3 equiv of aryl iodide, 5 mol % Pd(OAc)_2_, 2 equiv of AgOAc, and 1 equiv of NaOAc in DCE for 16 h at 100 °C. Here, it should be pointed out that we opted to continue with DCE based on the higher selectivity and yield of the C(sp^2^)–H arylation reaction in this solvent. However, if one wishes to perform this reaction on a larger scale, *tert*-amyl alcohol will serve excellently as a more process-friendly replacement for DCE. As shown by entry 16, it was possible to obtain product **4a** in a yield of 73%, when carrying out the C(sp^2^)–H arylation on a 2.5 mmol scale in *tert*-amyl alcohol.

As can be seen from the substrate scope study summarized in Figure 1, it was possible to introduce a wide range of aryl groups into compound **3** using the optimized reaction conditions. This C(sp^2^)–H arylation was found to work most efficiently with aryl iodides carrying electron-donating groups, as exemplified by the reactions giving products **4a–c**. Compared to product **4a** that was isolated in 74% yield [cf. 75% yield vs the internal standard (Table 1, entry 9)], products **4b** and **4c** were acquired in comparable yields (73% and 67%, respectively). Similar performance was also observed with iodobenzene as the arylating agent; however, due to co-elution issues between substrate **3** and product **4d** that complicated the column chromatography purification procedure, we were able to isolate **4d** in only 61% yield. Product **4e**, which originated from the arylation with 2-iodonaphthalene, could on the contrary be obtained in a significantly higher yield (76%).

**Figure 1:**
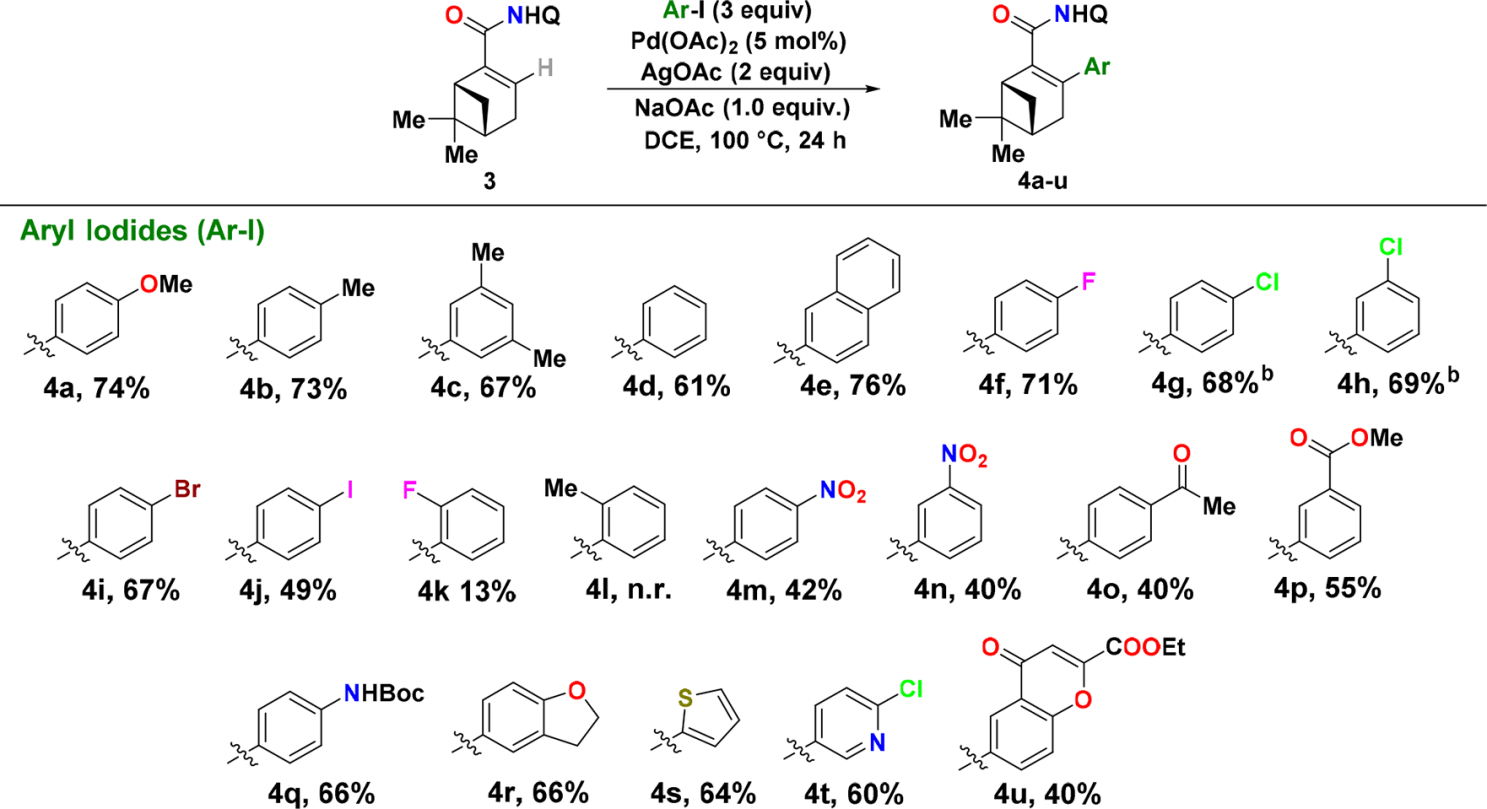
Scope of the Pd-catalyzed C–H arylation of substrate **3**. ^a^Reaction conditions: substrate **3** (0.15 mmol, Q = 8-quinolinyl), aryl iodide (3 equiv), Pd(OAc)_2_ (5 mol %), AgOAc (2.0 equiv), and NaOAc (1 equiv) were suspended in DCE (0.3 mL) and heated at 100 °C under an argon atmosphere for 24 h. All yields refer to isolated yields following silica gel chromatography. ^b^With a 48 h reaction time.

Satisfying results were also observed when *meta-* and *para-*halogenated aryl iodides were used, and in these cases, products **4f–j** could be obtained in 49–71% yield. However, in the reactions to form the chlorinated products **4g** and **4h**, it proved necessary to increase the reaction time to 48 h to push the reaction toward full conversion, simply because we were unable to separate starting material **3** from products **4g** and **4h** using column chromatography.^21^

Unfortunately, moving the substituent to the *ortho* position of the aryl iodide was found to be detrimental, as exemplified by the reactions of **4k** and **4l**. In addition, the use of aryl iodides carrying strong electron-withdrawing substituents, such as nitro, keto and ester groups, was also found to have a noticeable negative impact on the reaction efficiency. However, in all of these cases, products **4m–4p** could still be obtained in synthetically useful yields (40–55%). On the contrary, good performance was observed with an aryl iodide carrying a Boc-protected amino group, as exemplified by the reaction giving product **4q** in 66% yield.

To our delight, it also proved possible to install different heteroaromatic motifs into substrate **3**. For example, when 2,3-dihydro-5-iodo-benzofuran, 2-iodothiophene, and 2-chloro-5-iodopyridine were used as the heteroarylating agents, the corresponding products **4r–4t** were obtained in good yields (60–66%). The reaction with ethyl-6-iodo-4-oxo-4H-chromene-2-carboxylate, on the contrary, proved to be less efficient but still afforded product **4u** in a synthetically useful yield of 40%.

After having completed our survey of the aryl iodide scope of the C(sp^2^)–H arylation, we moved on to our next goal, which was to establish a synthetic pathway from myrtenal to the envisioned chiral cyclobutane keto acid derivatives. Aiming to provide a proof of this concept, we sought to develop a synthetic route to cyclobutane keto acid **6**, utilizing the C(sp^2^)–H arylation product **4a** as a model intermediate (Scheme 2). As mentioned previously, substrate **3** can be obtained from myrtenal in two simple and high-yielding synthetic steps. First, myrtenal (**1**) was oxidized to the corresponding carboxylic acid **2** in 80% yield using a previously reported NaClO_2_–H_2_O_2_-based protocol.^17^ Then, the 8-AQ auxiliary was installed onto **2** in 81% yield using a two-step sequence that proceeded via the intermediate acid chloride. As highlighted above, the C(sp^2^)–H arylation can be carried out efficiently on a 2.5 mmol scale with *tert*-amyl alcohol as the solvent to provide compound **4a** in 74% yield.

**Scheme 2:**
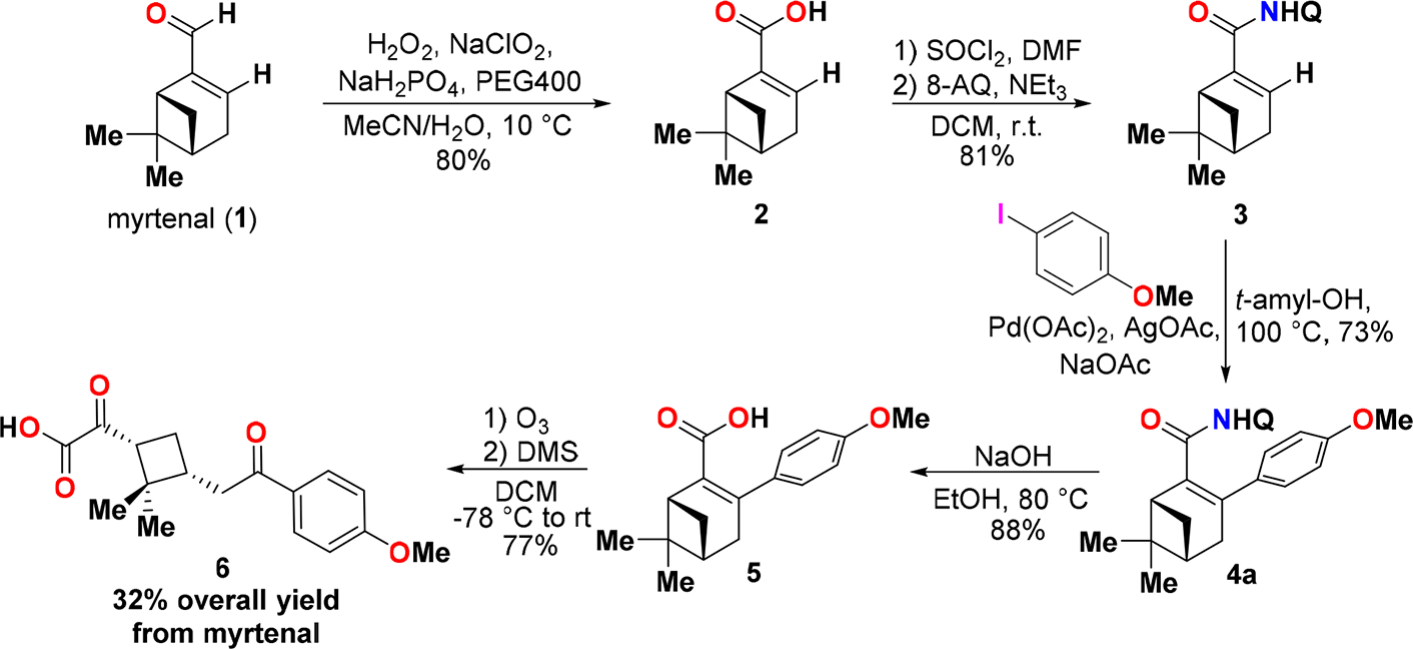
Proof-of-Concept Synthesis of a Cyclobutane Keto Acid Derivative from Myrtenal

From product **4a**, the next step was to remove the 8-AQ auxiliary, which could be accomplished in 88% yield using NaOH in EtOH. Finally, to access cyclobutane keto acid derivative **6**, we used an ozonolysis-based approach to open the bicyclic monoterpenoid scaffold of compound **5** over the C=C bond. This allowed us to obtain cyclobutane keto acid **6** with two stereocenters in 77% yield after just an extraction workup.

In summary, a concise synthetic route that allows access to cyclobutane keto acids with two stereocenters from the inexpensive terpene myrtenal from has been presented. As a proof of concept, we demonstrated the synthesis of cyclobutane keto acid **6** that was achieved in an overall yield of 32% over five steps. However, it should be pointed out that this synthetic route holds great potential to be used for the preparation of a wide range of such cyclobutane keto acid derivatives, as the C(sp^2^)–H arylation step allows for the introduction of considerable diversity. As we show herein, it was possible to introduce a wide range of aryl and heteroaryl substituents into this myrtenal-derived core, to give a variety C(sp^2^)–H-arylated products in good to high yields.

Given the many interesting biological activities displayed by cyclobutane derivatives,^22^ we foresee that our developed route will find applications related to the synthesis of small molecule libraries as well as novel natural product-like compounds.^23^

## Experimental Section

### General Experimental Information

All reagents and solvents were purchased and used as received from commercial vendors or synthesized according to cited procedures. With regard to the 1*R*-(−)-myrtenal (**1**) that was used in this work, it was obtained from Merck (98%, article no. 218243). Oxygen and/or moisture sensitive reactions were carried out in oven- or flame-dried glassware under a nitrogen atmosphere using appropriately dried solvents. Yields refer to chromatographically isolated compounds, unless otherwise stated. Room temperature in the laboratory was 21–23 °C. Flash chromatography was performed using 15–45 μm silica gel cartridges (60 Å mesh) on a Teledyne Isco Combiflash Rf. SiliaSep SiO_2_ cartridges used for these purifications were provided from SiliCycle. Analytical thin layer chromatography (TLC) was performed on 0.25 mm silica gel 60-F plates and visualized by UV light (254 nm) or suitable TLC stain. Chemical shifts are reported in parts per million relative to the NMR solvent peaks. Nuclear magnetic resonance spectra were recorded on a Bruker Advance spectrometer (^1^H, ^13^C, and ^19^F NMR). Deuterated solvents for NMR analyses was obtained from Sigma-Aldrich. Data for ^1^H NMR are reported as follows: chemical shift, multiplicity (br, broad; s, singlet; d, doublet; t, triplet; m, multiplet), integration, and coupling constants. High-resolution mass spectroscopy (HRMS) was performed on a Bruker microTOF/ESI mass spectrometer.

### General Method A: Optimization of the Pd-Catalyzed C–H Arylation of Substrate **4a** (Table 1)


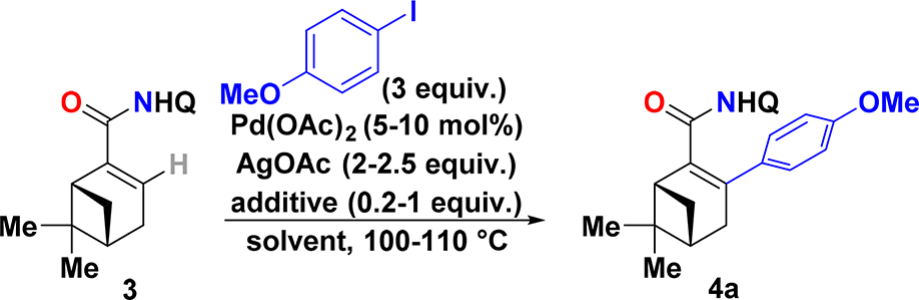
A small capped vial equipped with a stirring bar was charged with substrate **3** (0.15 mmol, 1.0 equiv), Pd(OAc)_2_ (5–10 mol %), AgOAc (0.3–0.375 mmol, 2.0–2.5 equiv), 4-iodoanisole (0.45 mmol, 3.0 equiv), and an additive (0.03–0.15 mmol, 0.2–1.0 equiv). All of the solids were then suspended in dry solvent, and the reaction vessel was evacuated and refilled with N_2_ before being placed in a preheated oil bath (at the given temperature) for the time given in Table 1. After completion of the reaction, the crude mixture was allowed to cool to rt. It was then diluted with EtOAc and filtered through a pad of Celite, and the filtrate was concentrated *in vacuo*. The crude reaction mixture was then redissolved in CDCl_3_ (3 mL), and 1,3,5-trimethoxybenzene (0.15 mmol) was added as an internal standard to allow for yield determinations by ^1^H NMR spectroscopy.

### General Method B: Scope of the Pd-Catalyzed C–H Arylation of Substrate **4a** (Figure 1)

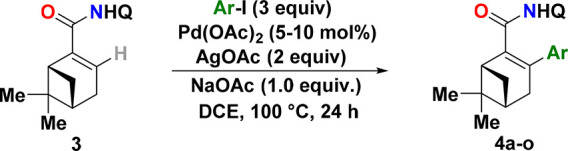



A small capped vial equipped with a stirring bar was charged with substrate **3** (43.9 mg, 0.15 mmol, 1.0 equiv), Pd(OAc)_2_ (1.68 or 3.36 mg, 5 or 10 mol %), AgOAc (50.1 mg, 0.3 mmol, 2.0 equiv), aryl halide (0.45 mmol, 3.0 equiv), and NaOAc (12.3 mg, 0.15 mmol, 1.0 equiv). All of the solids were then suspended in dry solvent, and the reaction vessel was evacuated and refilled with N_2_ before being placed in a preheated oil bath (at the given temperature) for the time given in Table 1. After completion of the reaction, the crude mixture was allowed to cool to rt. It was then diluted with EtOAc and filtered through a pad of Celite, and the filtrate was concentrated *in vacuo*. All products were purified by column chromatography (EtOAc/pentane gradients), and their yield and characterization data are presented below.

#### (1*R*,5*R*)-3-(4-Methoxyphenyl)-6,6-dimethyl-*N*-(quinolin-8-yl)bicyclo[3.1.1]hept-2-ene-2-carboxamide (**4a**)

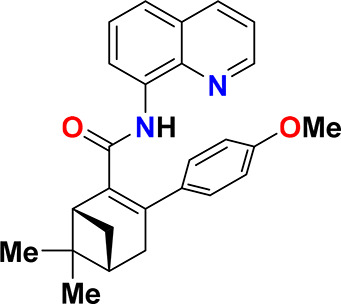



The reaction was performed according to general method B with 5 mol % Pd(OAc)_2_, and the product was isolated after column chromatography (gradient from 0% to 10% EtOAc in pentane) in 44.2 mg (74%) as a yellow amorphous solid: ^1^H NMR (400 MHz, CDCl_3_) δ 9.58 (s, 1H), 8.77 (dd, 1H, *J* = 7.65, 1.30 Hz), 8.44 (dd, 1H, *J* = 4.21 Hz, 1.69 Hz), 8.06 (dd, 1H, *J* = 8.28, 1.69 Hz), 7.52–7.48 (m, 1H), 7.41 (dd, 1H, *J* = 8.28, 1.30 Hz), 7.36–7.31 (m, 3H), 3.64 (s, 3H), 6.78–6.76 (m, 2H), 3.04 (t, 1H, *J* = 5.72 Hz), 2.84 (dd, 1H, *J* = 18.3, 2.97 Hz), 2.71 (dd, 1H, *J* = 18.3, 2.63 Hz), 2.63–2.58 (m, 1H), 2.32–2.28 (m, 1H), 1.47 (d, 1H, *J* = 9.06 Hz), 1.44 (s, 3H), 1.08 (s, 3H); ^13^C{^1^H} NMR (175 MHz, CDCl_3_) δ 168.1, 159.6, 147.3, 140.0, 138.5, 138.2, 136.5, 134.8, 132.2, 129.1, 127.9, 127.6, 121.24, 121.16, 116.6, 114.1, 55.3, 44.1, 40.8, 38.5, 38.4, 31.8, 26.0, 21.4 (700 MHz was necessary to resolve all ^13^C resonances, but we have also provided the corresponding 100 MHz ^13^C NMR spectrum of **4a** in the Supporting Information); HRMS (ESI) *m***/***z* [M + Na]^+^ calcd for C_26_H_26_N_2_O_2_Na 421.1886, found 421.1883.

#### (1*R*,5*R*)-3-(4-Methylphenyl)-6,6-dimethyl-*N*-(quinolin-8-yl)bicyclo[3.1.1]hept-2-ene-2-carboxamide (**4b**)

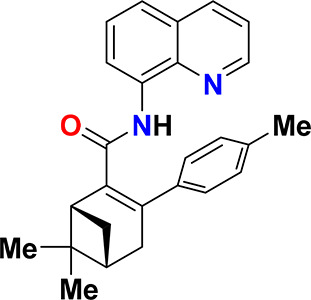



The reaction was performed according to general method B with 5 mol % Pd(OAc)_2_, and the product was isolated after column chromatography (gradient from 0% to 10% EtOAc in pentane) in 41.9 mg (73%) as a yellow amorphous solid: ^1^H NMR (400 MHz, CDCl_3_) δ 9.56 (s, 1H), 8.77 (dd, 1H, *J* = 7.66, 1.31 Hz), 8.42 (dd, 1H, *J* = 4.21, 1.69 Hz), 8.06 (dd, 1H, *J* = 8.27, 1.69 Hz), 7.51–7.47 (m, 1H), 7.42 (dd, 1H, *J* = 8.28, 1.31 Hz), 7.34–7.28 (m, 3H), 7.06–7.03 (m, 2H) 3.03 (t, 1H, *J* = 5.71 Hz), 2.85 (dd, 1H, *J* = 18.3, 2.95 Hz), 2.72 (dd, 1H, *J* = 18.3, 2.62 Hz), 2.64–2.58 (m, 1H), 2.32–2.27 (m, 1H), 2.19 (s, 3H), 1.48 (d, 1H, *J* = 9.07 Hz), 1.44 (s, 3H), 1.09 (s, 3H); ^13^C{^1^H} NMR (100 MHz, CDCl_3_) δ 167.7, 147.2, 140.2, 138.8, 138.4, 137.5, 136.8, 135.9, 134.8, 129.3, 129.1, 127.7, 127.3, 121.1, 120.9, 116.1, 44.0, 40.6, 38.34, 38.27, 31.7, 25.8, 21.2, 21.1; HRMS (ESI) *m***/***z* [M + Na]^+^ calcd for C_26_H_26_N_2_ONa 405.1937, found 405.1937.

#### (1*R*,5*R*)-3-(3,5-Dimethylphenyl)-6,6-dimethyl-*N*-(quinolin-8-yl)bicyclo[3.1.1]hept-2-ene-2-carboxamide (**4c**)

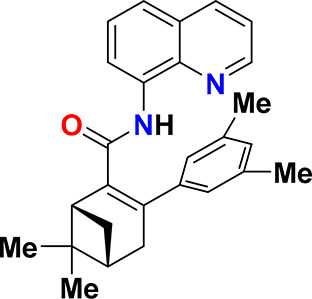



The reaction was performed according to general method B with 5 mol % Pd(OAc)_2_, and the product was isolated after column chromatography (gradient from 0% to 10% EtOAc in pentane) in 39.9 mg (67%) as a yellow amorphous solid: ^1^H NMR (400 MHz, CDCl_3_) δ 9.48 (s, 1H), 8.74 (dd, 1H, *J* = 7.65, 1.30 Hz), 8.40 (dd, 1H, *J* = 4.21, 1.70 Hz), 8.03 (dd, 1H, *J* = 6.60, 1.70 Hz), 7.48–7.44 (m, 1H), 7.39 (dd, 1H, *J* = 8.26, 1.30 Hz), 7.31 (dd, 1H, *J* = 8.26, 4.21), 6.99 (s, 2H), 6.72 (s, 1H), 3.01 (t, 1H, *J* = 5.69 Hz), 2.81 (dd, 1H, *J* = 18.4, 2.92 Hz), 2.77 (dd, 1H, *J* = 18.4, 2.63 Hz), 2.61–2.56 (m, 1H), 2.29–2.25 (m, 1H), 2.12 (s, 6H), 1.44 (d, 1H, *J* = 9.06 Hz), 1.42 (s, 3H), 1.07 (s, 3H); ^13^C{^1^H} NMR (100 MHz, CDCl_3_) δ 167.7, 147.3, 140.2, 139.6, 139.2, 138.4, 137.9, 135.8, 134.9, 129.3, 127.7, 127.3, 125.5, 121.1, 120.9, 116.0, 43.8, 40.6, 38.6, 38.2, 31.7, 21.21, 21.16; HRMS (ESI) *m***/***z* [M + Na]^+^ calcd for C_27_H_28_N_2_ONa 419.2094, found 419.2091.

#### (1*R*,5*R*)-6,6-Dimethyl-3-phenyl-*N*-(quinolin-8-yl)bicyclo[3.1.1]hept-2-ene-2-carboxamide (**4d**)

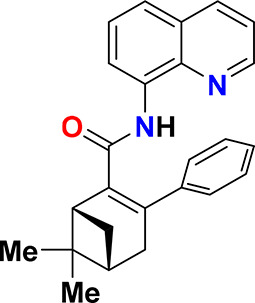



The reaction was performed according to general method B with 5 mol % Pd(OAc)_2_, and the product was isolated after column chromatography (gradient from 0% to 5% EtOAc in pentane) in 33.7 mg (61%) as a yellow amorphous solid: ^1^H NMR (400 MHz, CDCl_3_, 298 K) δ 9.50 (s, 1H), 8.75–8.73 (m, 1H), 8.40 (dd, 1H, *J* = 4.20, 1.69 Hz), 8.01 (dd, 1H, *J* = 8.03, 1.47 Hz), 7.48–7.44 (m, 1H), 7.41–7.37 (m, 3H), 7.29 (dd, 1H, *J* = 8.26, 4.20 Hz), 7.23–7.19 (m, 2H), 7.11 (tt, 1H, *J* = 7.42, 1.29 Hz), 3.02 (t, 1H, *J* = 5.73 Hz), 2.84 (dd, 1H, *J* = 18.4, 2.97 Hz), 2.71 (dd, 1H, *J* = 18.4, 2.62 Hz), 2.62–2.57 (m, 1H), 2.31–2.27 (m, 1H), 1.47 (d, 1H, *J* = 9.09 Hz), 1.43 (s, 3H), 1.08 (s, 3H); ^13^C{^1^H} NMR (100 MHz, CDCl_3_) δ 167.5, 147.4, 140.6, 139.7, 138.6, 138.4, 135.9, 134.7, 128.4, 127.8, 127.68, 127.65, 127.3, 121.2, 121.0, 116.0, 44.0, 40.6, 38.34, 38.26, 31.6, 25.8, 21.2; HRMS (ESI) *m***/***z* [M + Na]^+^ calcd for C_25_H_24_N_2_ONa 391.1781, found 391.1779.

#### (1*R*,5*R*)-6,6-Dimethyl-3-(naphthalen-2-yl)-*N*-(quinolin-8-yl)bicyclo[3.1.1]hept-2-ene-2-carboxamide (**4e**)

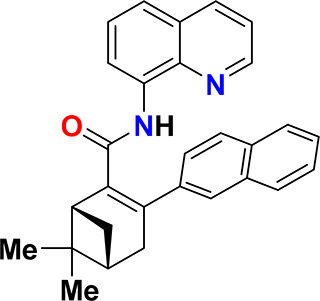



The reaction was performed according to general method B with 5 mol % Pd(OAc)_2_, and the product was isolated after column chromatography (gradient from 5% to 10% EtOAc in pentane) in 47.7 mg (76%) as a white amorphous solid: ^1^H NMR (400 MHz, CDCl_3_) δ 9.52 (s, 1H), 8.73 (dd, 1H, *J* = 7.73, 1.25 Hz), 7.96 (d, 1H, *J* = 1.13 Hz), 7.88–7.84 (m, 2H), 7.63 (dd, 1H, *J* = 7.99, 0.59 Hz), 7.57 (d, 1H, *J* = 8.54 Hz), 7.49–7.44 (m, 1H), 7.43–7.38 (m, 3H), 7.36 (dd, 1H, *J* = 4.23, 1.66 Hz), 7.31 (dd, 1H, *J* = 8.25, 1.25 Hz), 6.98 (dd, 1H, *J* = 8.25, 4.23 Hz), 3.13 (t, 1H, *J* = 5.71 Hz), 2.97 (dd, 1H, *J* = 18.5, 2.89 Hz), 2.82 (dd, 1H, *J* = 18.5, 2.63 Hz), 2.67–2.62 (m, 1H), 2.35–2.30 (m, 1H), 1.51 (d, 1H, *J* = 9.1 Hz), 1.46 (s, 3H), 1.13 (s, 3H); ^13^C{^1^H} NMR (100 MHz, CDCl_3_) δ 167.1, 147.0, 140.9, 139.2, 138.2, 137.4, 135.5, 134.6, 133.7, 133.1, 128.3, 128.0, 127.6, 127.4, 127.1 126.6, 126.4, 126.1, 125.8, 120.94, 120.90, 116.0, 43.7, 40.7, 39.0, 38.2, 31.7, 25.9, 21.3; HRMS (ESI) *m***/***z* [M + Na]^+^ calcd for C_29_H_26_N_2_ONa 441.1937, found 441.1941.

#### (1*R*,5*R*)-3-(4-Fluorophenyl)-6,6-dimethyl-*N*-(quinolin-8-yl)bicyclo[3.1.1]hept-2-ene-2-carboxamide (**4f**)

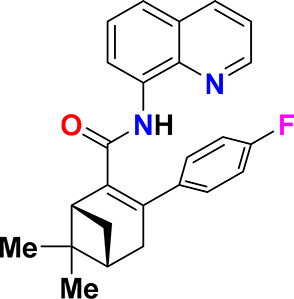



The reaction was performed according to general method B with 5 mol % Pd(OAc)_2_, and the product was isolated after column chromatography (gradient from 0% to 10% EtOAc in pentane) in 41.2 mg (71%) as a yellow amorphous solid: ^1^H NMR (400 MHz, CDCl_3_) δ 9.51 (s, 1H), 8.74 (dd, 1H, *J* = 7.59, 1.30 Hz), 8.45 (dd, 1H, *J* = 4.22, 1.66 Hz), 8.05 (dd, 1H, *J* = 8.28, 1.66 Hz), 7.50–7.46 (m, 1H), 7.42–7.31 (m, 4H), 6.94–6.90 (m, 2H), 3.01 (t, 1H, *J* = 5.70 Hz), 2.82 (dd, 1H, *J* = 18.3, 2.94 Hz), 2.68 (dd, 1H, *J* = 18.3, 2.64 Hz), 2.62–2.57 (m, 1H), 2.31–2.28 (m, 1H), 1.46 (d, 1H, *J* = 9.12 Hz), 1.43 (s, 3H), 1.07 (s, 3H); ^13^C{^1^H} NMR (100 MHz, CDCl_3_) δ 167.3, 162.6 (d, *J* = 246 Hz), 147.4, 141.0, 138.2, 137.4, 136.1, 135.7 (d, *J* = 3.67 Hz), 134.5, 129.5 (d, *J* = 8.07 Hz), 127.8, 127.3, 121.3, 121.2, 116.2, 115.4 (d, *J* = 21.8 Hz), 44.0, 40.6, 38.34, 38.29, 31.6, 25.8, 21.2; HRMS (ESI) *m***/***z* [M + Na]^+^ calcd for C_25_H_23_N_2_OFNa 409.1687, found 409.1685.

#### (1*R*,5*R*)-3-(4-Chlorophenyl)-6,6-dimethyl-*N*-(quinolin-8-yl)bicyclo[3.1.1]hept-2-ene-2-carboxamide (**4g**)

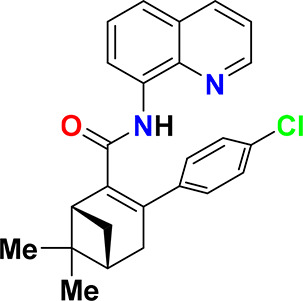



The reaction was performed according to general method B with 5 mol % Pd(OAc)_2_ for 48 h, and the product was isolated after column chromatography (gradient from 0% to 5% EtOAc in pentane) in 41.1 mg (68%) as a yellow amorphous solid: ^1^H NMR (400 MHz, CDCl_3_) δ 9.50 (s, 1H), 8.72 (dd, 1H, *J* = 7.55, 1.37 Hz), 8.46 (dd, 1H, *J* = 4.23, 1.69 Hz), 8.05 (d, 1H d, *J* = 8.29, 1.69 Hz), 7.49–7.45 (m, 1H), 7.36–7.31 (m, 4H), 7.21–7.17 (m, 2H), 3.02 (t, 1H, *J* = 5.69 Hz), 2.80 (dd, 1H, *J* = 18.3, 2.93 Hz), 2.67 (dd, 1H, *J* = 18.3, 2.64 Hz), 2.62–2.57 (m, 1H), 2.31–2.28 (m, 1H), 1.45 (d, 1H, *J* = 9.14 Hz), 1.42 (s, 3H), 1.06 (s, 3H); ^13^C{^1^H} NMR (100 MHz, CDCl_3_) δ 167.1, 147.6, 141.3, 138.3, 138.2, 137.3, 136.1, 134.5, 133.7, 129.2, 128.7, 127.7, 127.3, 121.33, 121.27, 116.2, 44.0, 40.6, 38.3, 38.2, 31.6, 25.8, 21.2; HRMS (ESI) *m***/***z* [M + Na]^+^ calcd for C_25_H_23_N_2_OClNa 425.1391, found 425.1401.

#### (1*R*,5*R*)-3-(3-Chlorophenyl)-6,6-dimethyl-*N*-(quinolin-8-yl)bicyclo[3.1.1]hept-2-ene-2-carboxamide (**4h**)

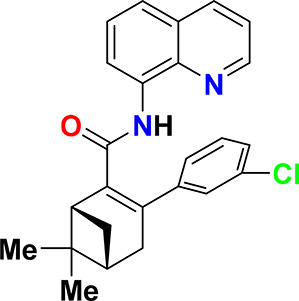



The reaction was performed according to general method B with 5 mol % Pd(OAc)_2_ for 48 h, and the product was isolated after column chromatography (gradient from 0% to 5% EtOAc in pentane) in 42.0 mg (69%) as a white amorphous solid: ^1^H NMR (500 MHz, CDCl_3_) δ 9.47 (s, 1H), 8.71 (d, 1H, *J* = 8.71 Hz), 8.51 (dd, 1H, *J* = 4.15, 1.39 Hz), 8.03 (dd, 1H, *J* = 8.23, 1.24 Hz), 7.49–7.43 (m, 2H), 7.40 (d, 1H, *J* = 8.25 Hz), 7.33 (dd, 1H, *J* = 8.23, 4.15 Hz), 7.21–7.18 (m, 1H), 7.10–7.07 (m, 1H), 7.05–7.00 (m, 1H), 3.01 (t, 1H, *J* = 5.65 Hz), 2.80 (dd, 1H, *J* = 18.3, 2.71 Hz), 2.68 (dd, 1H, *J* = 18.3, 2.41 Hz), 2.63–2.58 (m, 1H), 2.31–2.28 (m, 1H), 1.44 (d, 1H, *J* = 9.34 Hz), 1.43 (s, 3H), 1.07 (s, 3H); ^13^C{^1^5H} NMR (125 MHz, CDCl_3_) δ 167.0, 147.8, 141.9, 141.7, 138.4, 137.2, 135.9, 134.50, 134.47, 129.8, 127.8, 127.7 (two overlapping C), 127.3, 126.4, 121.4, 121.2, 116.0, 44.0, 40.6, 38.3 (two overlapping C), 31.6, 25.9, 21.2; HRMS (ESI) *m***/***z* [M + Na]^+^ calcd for C_25_H_23_N_2_OClNa 425.1391, found 425.1374.

#### (1*R*,5*R*)-3-(4-Bromophenyl)-6,6-dimethyl-*N*-(quinolin-8-yl)bicyclo[3.1.1]hept-2-ene-2-carboxamide (**4i**)

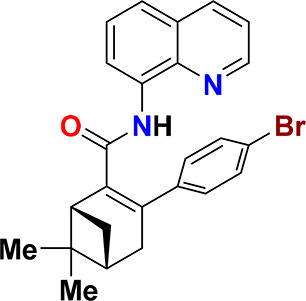



The reaction was performed according to general method B with 5 mol % Pd(OAc)_2_, and the product was isolated after column chromatography (gradient from 5% to 10% EtOAc in pentane) in 45.0 mg (67%) as a white amorphous solid: ^1^H NMR (400 MHz, CDCl_3_) δ 9.48 (s, 1H), 8.71 (dd, 1H, *J* = 7.50, 1.43 Hz), 8.46 (dd, 1H, *J* = 4.22, 1.70 Hz), 8.05 (dd, 1H, *J* = 8.26, 1.70 Hz), 7.50–7.45 (m, 1H), 7.42 (dd, 1H, *J* = 8.26, 1.48 Hz), 7.36–7.33 (m, 3H), 7.27–7.25 (m, 2H + CDCl_3_ peak), 3.02 (t, 1H, *J* = 5.70 Hz), 2.81 (dd, 1H, *J* = 18.6, 2.95 Hz), 2.68 (dd, 1H, *J* = 18.6, 2.67 Hz), 2.62–2.57 (m, 1H), 2.31–2.28 (m, 1H), 1.43 (d, 1H), 1.42 (s, 3H), 1.05 (s, 3H); ^13^C{^1^H} NMR (100 MHz, CDCl_3_) δ 167.1, 147.7, 141.3, 138.6, 138.3, 137.3, 135.9, 134.5, 131.6, 129.5, 127.7, 127.2, 121.9, 121.34, 121.25, 116.1, 44.0, 40.5, 38.2, 38.1, 31.5, 25.8, 21.2; HRMS (ESI) *m***/***z* [M + Na]^+^ calcd for C_25_H_23_N_2_OBr^79^Na and C_25_H_23_N_2_OBr^81^Na 469.0886 and 471.0867, found 469.0890 and 471.0863.

#### (1*R*,5*R*)-3-(4-Iodophenyl)-6,6-dimethyl-*N*-(quinolin-8-yl)bicyclo[3.1.1]hept-2-ene-2-carboxamide (**4j**)

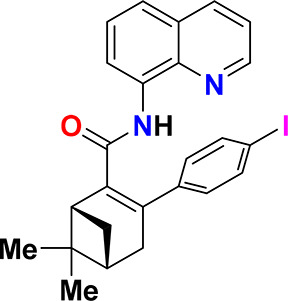



The reaction was performed according to general method B with 5 mol % Pd(OAc)_2_, and the product was isolated after column chromatography (gradient from 0% to 10% EtOAc in pentane) in 36.3 mg (49%) as a yellow amorphous solid: ^1^H NMR (400 MHz, CDCl_3_) δ 9.49 (s, 1H), 8.71 (dd, 1H, *J* = 7.51, 1.19 Hz), 8.48 (dd, 1H, *J* = 4.21, 1.63 Hz), 8.07 (dd, 1H, *J* = 8.27, 1.63 Hz), 7.57–7.52 (m, 2H), 7.50–7.42 (m, 2H), 7.37 (dd, 1H, *J* = 8.27, 4.21 Hz), 7.15–7.11 (m, 2H), 3.01 (t, 1H, *J* = 5.67 Hz), 2.80 (dd, 1H, *J* = 18.3, 2.95 Hz), 2.67 (dd, 1H, *J* = 18.3, 2.61 Hz), 2.62–2.56 (m, 1H), 2.31–2.27 (m, 1H), 1.44 (d, 1H, *J* = 9.29 Hz), 1.42 (s, 3H), 1.05 (s, 3H); ^13^C{^1^H} NMR (100 MHz, CDCl_3_) δ 161.7, 147.7, 141.3, 139.2, 138.2, 137.5, 137.4, 136.1, 134.4, 129.8, 127.8, 127.3, 121.4, 121.3, 116.3, 93.5, 44.0, 40.5, 38.2, 38.0, 31.5, 25.8, 21.2; HRMS (ESI) *m***/***z* [M + Na]^+^ calcd for C_25_H_23_N_2_OINa 517.0747, found 517.0739.

#### (1*R*,5*R*)-3-(2-Fluorophenyl)-6,6-dimethyl-*N*-(quinolin-8-yl)bicyclo[3.1.1]hept-2-ene-2-carboxamide (**4k**)

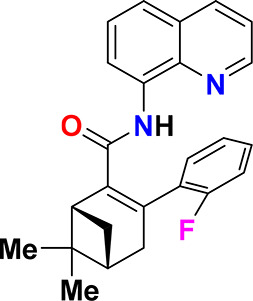



The reaction was performed according to general method B with 5 mol % Pd(OAc)_2_, and the product was isolated after column chromatography (gradient from 5% to 10% EtOAc in pentane) in 7.5 mg (13%) as a yellow amorphous solid: ^1^H NMR (400 MHz, CDCl_3_) δ 9.63 (s, 1H), 8.72 (dd, 1H, *J* = 7.56, 1.30 Hz), 8.48 (dd, 1H, *J* = 4.19, 1.63 Hz), 8.03 (dd, 1H, *J* = 8.26, 1.63 Hz), 7.48–7.44 (m, 1H), 7.39 (dd, 1H, *J* = 8.26, 1.37 Hz), 7.35–7.30 (m, 2H), 7.14–7.09 (m, 1H), 7.03–6.97 (m, 2H), 3.05 (t, 1H, *J* = 5.72 Hz), 2.83 (dd, 1H, *J* = 18.4, 3.04 Hz), 2.69 (dd, 1H, *J* = 18.4, 2.61 Hz), 2.63–2.59 (m, 1H), 2.30–2.26 (m, 1H), 1.50 (d, 1H, *J* = 9.11 Hz), 1.44 (s, 3H), 1.12 (s, 3H); ^13^C{^1^H} NMR (100 MHz, CDCl_3_) δ 166.6, 159.9 (d, *J* = 246 Hz), 147.4, 142.9, 138.3, 136.0, 134.5, 133.4, 130.3, 130.2, 129.3 (d, *J* = 8.07 Hz), 127.7, 127.4, 127.3 (d, *J* = 16.1 Hz), 124.3 (d, *J* = 3.65 Hz), 121.1 (d, *J* = 16.1 Hz), 116.1 (d, *J* = 22.1 Hz), 115.7, 43.8, 40.6, 38.2, 38.0 (d, *J* = 1.47 Hz), 31.6, 25.9, 21.1; HRMS (ESI) *m***/***z* [M + Na]^+^ calcd for C_25_H_23_N_2_OFNa 409.1687, found 409.1682.

#### (1*R*,5*R*)-3-(4-Nitrophenyl)-6,6-dimethyl-*N*-(quinolin-8-yl)bicyclo[3.1.1]hept-2-ene-2-carboxamide (**4m**)

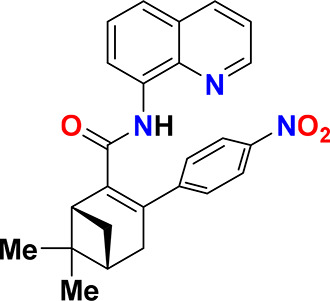



The reaction was performed according to general method B with 5 mol % Pd(OAc)_2_, and the product was isolated after column chromatography (gradient from 0% to 10% EtOAc in pentane) in 26.0 mg (42%) as a yellow amorphous solid: ^1^H NMR (400 MHz, CDCl_3_) δ 9.44 (s, 1H), 8.69 (dd, 1H, *J* = 7.41, 1.41 Hz), 8.40 (dd, 1H, *J* = 4.25, 1.68 Hz), 8.08–8.04 (m, 3H), 7.57–7.54 (m, 2H), 7.50–7.42 (m, 2H), 7.31 (dd, 1H, *J* = 8.27, 4.25 Hz), 3.00 (t, *J* = 5.66 Hz, 1H), 2.86 (dd, 1H, *J* = 18.3, 2.92 Hz), 2.72 (dd, 1H, *J* = 18.3, 2.64 Hz), 2.67–2.62 (m, 1H), 2.37–2.33 (m, 1H), 1.49 (d, 1H, *J* = 9.23 Hz), 1.45 (s, 3H), 1.09 (s, 3H); ^13^C{^1^H} NMR (100 MHz, CDCl_3_) δ 166.7, 147.6, 147.3, 146.7, 143.6, 138.2, 136.3, 135.9, 134.1, 128.7, 127.8, 127.4, 123.8, 121.7, 121.5, 116.4, 44.4, 40.5, 38.4, 37.6, 31.5, 25.8, 21.3; HRMS (ESI) *m***/***z* [M + Na]^+^ calcd for C_25_H_23_N_3_O_3_Na 436.1632, found 436.1623.

#### (1*R*,5*R*)-3-(3-Nitrophenyl)-6,6-dimethyl-*N*-(quinolin-8-yl)bicyclo[3.1.1]hept-2-ene-2-carboxamide (**4n**)

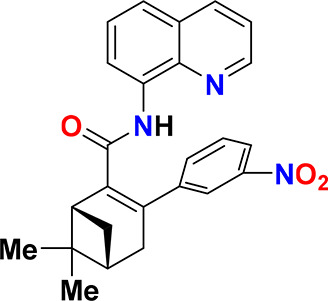



The reaction was performed according to general method B with 5 mol % Pd(OAc)_2_, and the product was isolated after column chromatography (gradient from 0% to 10% EtOAc in pentane) in 25.0 mg (40%) as a white amorphous solid: ^1^H NMR (500 MHz, CDCl_3_) δ 9.40 (s, 1H), 8.65 (d, 1H, *J* = 7.44 Hz), 8.33 (dd, 1H, *J* = 4.20, 1.38 Hz), 8.31–8.29 (m, 1H), 8.01 (dd, 1H, *J* = 8.24, 1.38 Hz), 7.92 (d, 1H, *J* = 8.55 Hz), 7.62 (d, 1H, *J* = 7.64 Hz), 7.44–7.41 (m, 1H), 7.39–7.37 (m, 1H), 7.28 (dd, 1H, *J* = 8.24, 4.20 Hz) 7.24–7.21 (m, 1H), 2.97 (t, 1H, *J* = 5.60 Hz), 2.83 (dd, 1H, *J* = 18.3, 2.76 Hz), 2.71 (dd, 1H, *J* = 18.3, 2.40 Hz), 2.63–2.59 (m, 1H), 2.34–2.29 (m, 1H), 1.47 (d, 1H, *J* = 9.25 Hz), 1.42 (s, 3H), 1.08 (s, 3H); ^13^C{^1^H} NMR (125 MHz, CDCl_3_) δ 166.7, 148.4, 147.8, 143.3, 141.6, 138.2, 136.2, 135.8, 134.4, 134.1, 129.5, 127.8, 127.3, 122.52, 122.49, 121.6, 121.5, 116.2, 44.3, 40.5, 38.4, 37.9, 31.6, 25.8, 21.3; HRMS (ESI) *m***/***z* [M + Na]^+^ calcd for C_25_H_23_N_3_O_3_Na 436.1632, found 436.1642.

#### (1*R*,5*R*)-3-(4-Acetylphenyl)-6,6-dimethyl-*N*-(quinolin-8-yl)bicyclo[3.1.1]hept-2-ene-2-carboxamide (**4o**)

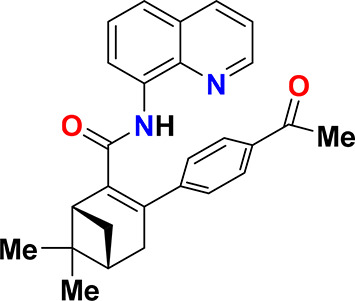



The reaction was performed according to general method B with 5 mol % Pd(OAc)_2_, and the product was isolated after column chromatography (gradient from 10% to 30% EtOAc in pentane) in 24.6 mg (40%) as a yellow amorphous solid: ^1^H NMR (400 MHz, CDCl_3_) δ 9.45 (s, 1H), 8.71 (dd, 1H, *J* = 7.60, 1.21 Hz), 8.35 (dd, 1H, *J* = 4.22, 1.67 Hz), 8.02 (dd, 1H, *J* = 8.28, 1.67 Hz), 7.80–7.77 (m, 2H), 7.50–7.45 (m, 3H), 7.40 (dd, 1H, *J* = 8.27, 1.36 Hz), 7.27 (dd, 1H, *J* = 8.28, 4.22 Hz), 3.00 (t, 1H, *J* = 5.67 Hz), 2.85 (dd, 1H, *J* = 18.3, 2.97 Hz), 2.71 (dd, 1H, *J* = 18.3, 2.63 Hz), 2.65–2.59 (m, 1H), 2.39 (s, 3H), 2.33–2.29 (m, 1H), 1.48 (d, 1H, *J* = 9.17 Hz), 1.43 (s, 3H), 1.09 (s, 3H); ^13^C{^1^H} NMR (100 MHz, CDCl_3_) δ 197.6, 167.1, 147.5, 144.7, 142.3, 138.3, 137.2, 136.3, 136.0, 134.4, 128.5, 128.0, 127.7, 127.3, 121.33, 121.26, 116.2, 44.2, 40.5, 38.3, 37.7, 31.5, 26.5, 25.8, 21.2; HRMS (ESI) *m***/***z* [M + Na]^+^ calcd for C_27_H_26_N_2_O_2_Na 433.1886, found 433.1883.

#### Methyl 3-[(1*R*,5*R*)-6,6-Dimethyl-2-(quinolin-8-ylcarbamoyl)bicyclo[3.1.1]hept-2-en-3-yl]benzoate (**4p**)

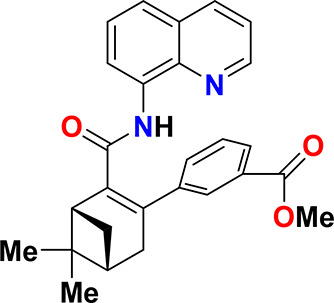



The reaction was performed according to general method B with 5 mol % Pd(OAc)_2_, and the product was isolated after column chromatography (gradient from 5% to 30% EtOAc in pentane) in 35.0 mg (55%) as a white amorphous solid: ^1^H NMR (400 MHz, CDCl_3_) δ 9.44 (s, 1H), 8.71 (dd, 1H, *J* = 7.60, 1.24 Hz), 8.32 (dd, 1H, *J* = 4.21, 1.68 Hz), 8.11 (t, 1H, *J* = 1.63 Hz), 8.01 (dd, 1H, *J* = 8.28, 1.63 Hz), 7.79 (dt, 1H, *J* = 7.80, 1.28 Hz), 7.56–7.52 (m, 1H), 7.47–7.43 (m, 1H), 7.38 (dd, 1H, *J* = 8.26, 1.34 Hz), 7.27 (dd, 1H, *J* = 8.28, 4.21 Hz), 7.21 (td, 1H, *J* = 7.72, 0.40 Hz), 3.88 (s, 3H), 3.02 (t, 1H, *J* = 5.68 Hz), 2.85 (dd, 1H, *J* = 18.4, 2.94 Hz), 2.73 (dd, 1H, *J* = 18.4, 2.61 Hz), 2.64–2.58 (m, 1H), 2.33–2.28 (m, 1H), 1.47 (d, 1H, *J* = 9.15 Hz), 1.43 (s, 3H), 1.10 (s, 3H); ^13^C{^1^H} NMR (100 MHz, CDCl_3_) δ 167.1, 167.0, 147.6, 141.7, 140.1, 138.3, 137.5, 135.9, 134.5, 132.7, 130.4, 128.9, 128.7 (two overlapping C), 127.7, 127.3, 121.3, 121.2, 116.0, 52.2, 44.1, 40.6, 38.31, 38.29, 31.6, 25.9, 21.3; HRMS (ESI) *m***/***z* [M + Na]^+^ calcd for C_27_H_26_N_2_O_3_Na 449.1836, found 449.1851.

#### *tert*-Butyl {4-[(1*R*,5*R*)-6,6-Dimethyl-2-(quinolin-8-ylcarbamoyl)bicyclo[3.1.1]hept-2-en-3-yl]phenyl}carbamate (**4q**)

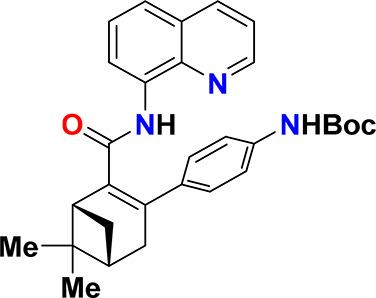



The reaction was performed according to general method B with 5 mol % Pd(OAc)_2_, and the product was isolated after column chromatography (gradient from 5% to 30% EtOAc in pentane) in 47.9 mg (66%) as a white amorphous solid: ^1^H NMR (500 MHz, CDCl_3_) δ 9.55 (s, 1H), 8.72 (d, 1H, *J* = 7.52 Hz), 8.47 (d, 1H, *J* = 4.10 Hz), 8.02 (d, 1H, *J* = 8.33 Hz), 7.49–7.44 (m, 1H), 7.39 (d, 1H, *J* = 7.90 Hz), 7.33–7.30 (m, 2H), 7.28 (dd, 1H, *J* = 8.33, 4.10 Hz), 7.23–7.18 (m, 2H), 6.27 (s, 1H), 2.99 (t, 1H, *J* = 5.62 Hz), 2.82 (dd, 1H, *J* = 18.3, 2.72 Hz), 2.68 (dd, 1H, *J* = 18.3, 2.35 Hz), 2.60–2.55 (m, 1H), 2.29–2.25 (m, 1H), 1.48 (s, 9H), 1.44 (d, 1H, *J* = 9.10 Hz), 1.41 (s, 3H), 1.05 (s, 3H); ^13^C{^1^H} NMR (125 MHz, CDCl_3_) δ 167.7, 152.5, 147.7, 140.4, 138.5, 138.2, 138.1, 135.8, 134.8, 134.3, 128.5, 127.7, 127.3, 121.2, 121.0, 118.4, 116.0, 80.6, 44.0, 40.7, 38.4, 38.2, 31.7, 28.3, 25.9, 21.3; HRMS (ESI) *m***/***z* [M + Na]^+^ calcd for C_30_H_33_N_3_O_3_Na 506.2414, found 506.2391.

#### (1*R*,5*R*)-3-(2,3-Dihydrobenzofuran-5-yl)-6,6-dimethyl-*N*-(quinolin-8-yl)bicyclo[3.1.1]hept-2-ene-2-carboxamide (**4r**)

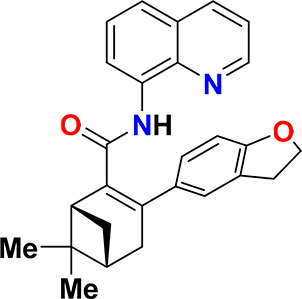



The reaction was performed according to general method B with 5 mol % Pd(OAc)_2_, and the product was isolated after column chromatography (gradient from 5% to 30% EtOAc in pentane) in 42.0 mg (68%) as a white amorphous solid: ^1^H NMR (400 MHz, CDCl_3_) δ 9.56 (s, 1H), 8.75 (dd, 1H, *J* = 7.66, 1.21 Hz), 8.43 (dd, 1H, *J* = 4.29, 1.62 Hz), 8.04 (dd, 1H, *J* = 8.28, 1.62 Hz), 7.49–7.46 (m, 1H), 7.40 (dd, 1H, *J* = 8.26, 1.31 Hz), 7.32 (dd, 1H, *J* = 8.28, 4.20 Hz), 7.20–7.15 (m, 2H), 6.70 (d, 1H, *J* = 8.12 Hz), 4.37–4.24 (m, 2H), 3.02 (t, 1H, *J* = 5.72 Hz), 2.94–2.75 (m, 3H), 2.68 (dd, 1H, *J* = 18.3, 2.61 Hz), 2.60–2.55 (m, 1H), 2.29–2.24 (m, 1H), 1.44–1.41 (overlapping s and d, 4H), 1.05 (s, 3H); ^13^C{^1^H} NMR (100 MHz, CDCl_3_) δ 167.9, 160.1, 147.5, 139.7, 139.0, 138.4, 135.8, 134.9, 131.9, 127.7, 127.5, 127.32, 127.31, 124.7, 121.2, 120.9, 116.0, 109.2, 71.2, 43.9, 40.7, 38.6, 38.3, 31.7, 29.7, 29.4, 25.8, 21.2; HRMS (ESI) *m***/***z* [M + Na]^+^ calcd for C_27_H_26_N_2_O_2_Na 433.1886, found 433.1903.

#### (1*R*,5*R*)-6,6-Dimethyl-*N*-(quinolin-8-yl)-3-(thiophen-2-yl)bicyclo[3.1.1]hept-2-ene-2-carboxamide (**4s**)

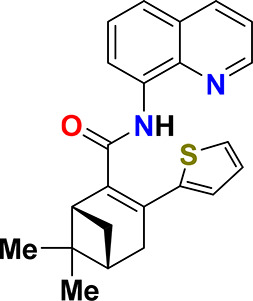



The reaction was performed according to general method B with 5 mol % Pd(OAc)_2_, and the product was isolated after column chromatography (gradient from 0% to 10% EtOAc in pentane) in 36.0 mg (64%) as an olive-colored amorphous solid: ^1^H NMR (400 MHz, CDCl_3_) δ 9.86 (s, 1H), 8.81 (dd, 1H, *J* = 7.60, 1.32 Hz), 8.59 (dd, 1H, *J* = 4.22, 1.68 Hz), 8.10 (dd, 1H, *J* = 8.27, 1.68 Hz), 7.55–7.51 (m, 1H), 7.47 (dd, 1H, *J* = 8.29, 1.39 Hz), 7.37 (dd, 1H, *J* = 8.27, 4.22 Hz), 7.15–7.12 (m, 2H), 6.83–6.81 (m, 1H), 2.91–2.85 (m, 2H), 2.77 (dd, 1H, *J* = 17.8, 2.69 Hz), 2.62–2.57 (m, 1H), 2.32–2.28 (m, 1H), 1.52 (d, 1H, *J* = 9.11 Hz), 1.41 (s, 3H), 1.09 (s, 3H); ^13^C{^1^H} NMR (100 MHz, CDCl_3_) δ 168.0, 147.8, 141.1, 140.8, 138.3, 136.2, 134.7, 128.7, 127.9, 127.4, 127.1, 125.9, 125.3, 121.4 (2C),^24^ 116.5, 45.0, 40.6, 39.1, 37.8, 31.7, 25.8, 21.4; HRMS (ESI) *m***/***z* [M + Na]^+^ calcd for C_23_H_22_N_2_OSNa 397.1345, found 397.1339.

#### (1*R*,5*R*)-3-(6-Chloropyridin-3-yl)-6,6-dimethyl-*N*-(quinolin-8-yl)bicyclo[3.1.1]hept-2-ene-2-carboxamide (**4t**)

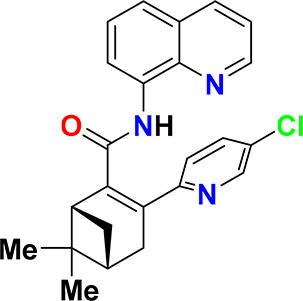



The reaction was performed according to general method B with 5 mol % Pd(OAc)_2_, and the product was isolated after column chromatography (gradient from 5% to 10% EtOAc in pentane) in 36.4 mg (60%) as a white amorphous solid: ^1^H NMR (400 MHz, CDCl_3_) δ 9.50 (s, 1H), 8.67 (dd, 1H, *J* = 7.28, 1.68 Hz), 8.52 (dd, 1H, *J* = 4.23, 1.68 Hz), 8.45 (dd, 1H, *J* = 2.49, 0.60 Hz), 8.08 (dd, 1H, *J* = 8.29, 1.68 Hz), 7.61 (dd, 1H, *J* = 8.22, 2.49 Hz), 7.50–7.43 (m, 2H), 7.37 (dd, 1H, *J* = 8.29, 4.23 Hz), 7.10 (dd, 1H, *J* = 8.22, 0.60 Hz), 3.01 (t, 1H, *J* = 5.67 Hz), 2.83 (dd, 1H, *J* = 18.3, 2.97 Hz), 2.68 (dd, 1H, *J* = 18.3, 2.65 Hz), 2.64–2.62 (m, 1H), 2.34–2.32 (m, 1H), 1.46 (d, 1H, *J* = 9.23 Hz), 1.44 (s, 3H), 1.06 (s, 3H); ^13^C{^1^H} NMR (100 MHz, CDCl_3_) δ 166.4, 150.6, 148.3, 148.0, 143.5, 138.3, 136.2, 134.4, 134.0, 133.5, 128.8, 127.8, 127.3, 124.0, 121.6, 121.5, 116.3, 44.2, 40.5, 38.2, 37.8, 31.4, 25.7, 21.2; HRMS (ESI) *m***/***z* [M + Na]^+^ calcd for C_24_H_22_N_3_OClNa 426.1344, found 426.1340.

#### Ethyl 6-[(1*R*,5*R*)-6,6-Dimethyl-2-(quinolin-8-ylcarbamoyl)bicyclo[3.1.1]hept-2-en-3-yl]-4-oxo-4*H*-chromene-2-carboxylate (**4u**)

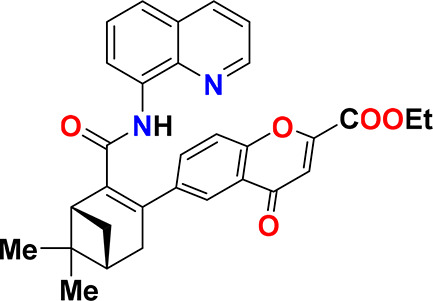



The reaction was performed according to general method B with 5 mol % Pd(OAc)_2_, and the product was isolated after column chromatography (gradient from 5% to 10% EtOAc in pentane) in 30.5 mg (40%) as a yellow amorphous solid: ^1^H NMR (400 MHz, CDCl_3_) δ 9.45 (s, 1H), 8.68 (dd, 1H, *J* = 7.54, 0.99 Hz), 8.29 (d, 1H, *J* = 2.13 Hz), 8.26 (dd, 1H, *J* = 4.21, 1.64 Hz), 8.00 (dd, 1H, *J* = 8.29, 1.64 Hz), 7.70 (dd, 1H, *J* = 8.70, 2.26 Hz), 7.47–7.43 (m, 1H), 7.38 (dd, 1H, *J* = 8.26, 1.30 Hz), 7.31 (d, 1H, *J* = 8.70 Hz), 7.22 (dd, 1H, *J* = 8.29, 4.21 Hz), 7.09 (s, 1H), 4.41 (q, 2H, *J* = 7.13 Hz), 2.98 (t, 1H, *J* = 5.627 Hz), 2.87 (dd, 1H, *J* = 18.4, 2.91 Hz), 2.77 (dd, 1H, *J* = 18.4, 2.58 Hz), 2.66–2.60 (m, 1H), 2.35–2.32 (m, 1H), 1.49 (d, 1H, *J* = 9.19 Hz), 1.43 (s, 3H), 1.40 (t, 3H, *J* = 7.13 Hz), 1.10 (s, 3H); ^13^C{^1^H} NMR (100 MHz, CDCl_3_) δ 178.3, 167.0, 160.4, 155.5, 152.1, 147.5, 142.8, 138.3, 138.0, 136.3, 136.0, 135.2, 134.2, 127.7, 127.3, 124.3, 124.0, 121.4, 121.3, 119.0, 116.1, 114.6, 63.0, 44.2, 40.5, 38.4, 38.0, 31.6, 25.8, 21.3, 14.1; HRMS (ESI) *m***/***z* [M + Na]^+^ calcd for C_31_H_28_N_2_O_5_Na 531.1890, found 531.1882.

### Synthesis of (1*R*)-Myrtenic Acid (**2**)

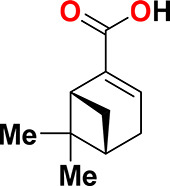



A solution of NaClO_2_ (8.0 g, 70 mmol, 1.4 equiv) in H_2_O (70 mL) was added slowly over 2 h to a stirred mixture of myrtenal (7.7 g, 50 mmol, 1 equiv), NaH_2_PO_4_ (1.6 g, 13 mmol, 0.26 equiv), H_2_O_2_ (35%, 5.0 mL, 52 mmol, 1.04 equiv), and polyethylene glycol (PEG-400, 3.0 g) in CH_3_CN (50 mL) and water (20 mL) at 10 °C. The reaction mixture was stirred for 7 h, after which the reaction was quenched with Na_2_SO_3_ (0.5 g). The resulting mixture was acidified to pH 3 with 10% aqueous HCl and extracted five times with diethyl ether. The organic layers were collected, combined, washed with saturated NaHSO_3_ and deionized H_2_O, and dried over anhydrous Na_2_SO_4_. The organic layer was then concentrated *in vacuo* to furnish compound **2** as a colorless viscous liquid (6.65 g, 80% yield). No further purification of compound **2** was needed, and its characterization data were in accordance with those previously reported.^16^

### Synthesis of 8-AQ Amide Substrate **3**

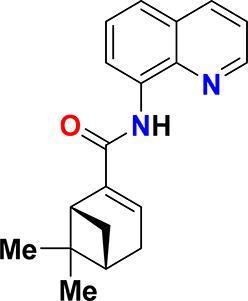



Oxalyl chloride (0.9 mL, 10.5 mmol, 2.1 equiv) was added slowly to a stirred solution of (1*R*)-myrtenic acid (**2**, 0.83 g, 5 mmol, 1 equiv) and catalytic amounts of DMF (2–3 drops) in DCM (10 mL) at 0 °C. The reaction mixture was then allowed to reach rt and stirred for 5 h, after which it was concentrated *in vacuo*. The obtained crude acid chloride was dissolved in DCM (5 mL) and added to a solution of 8-aminoquinoline (0.72 g, 5 mmol, 1 equiv) and triethylamine (0.7 mL, 5 mmol, 1 equiv) in DCM (10 mL) at 0 °C. The subsequent reaction mixture was allowed to reach rt and stirred overnight (∼14 h). The reaction mixture was then washed in a separatory funnel with a saturated sodium bicarbonate solution (2 × 20 mL), dried, and concentrated *in vacuo*. Purification by column chromatography (gradient from 0% to 10% EtOAc in pentane) afforded the desired products as a white amorphous solid (1.18 g, 81% yield): ^1^H NMR (400 MHz, CDCl_3_) δ 10.29 (s, 1H), 8.33–8.31 (m, 2H), 8.14–8.12 (m, 1H), 7.54–7.41 (m, 3H), 6.73–6.72 (m, 1H), 2.97 (td, 1H, *J* = 5.60, 1.71 Hz), 2.58–2.45 (m, 3H), 2.20–2.18 (m, 1H), 1.41 (s, 3H), 1.26 (d, 1H, *J* = 9.10 Hz), 0.90 (s, 3H); ^13^C{^1^H} NMR (100 MHz, CDCl_3_) δ 165.3, 148.1, 144.8, 138.7, 136.3, 134.8, 130.2, 128.0, 127.5, 121.5, 121.2, 116.2, 41.8, 40.5, 37.9, 32.0, 31.5, 26.0, 21.1; HRMS (ESI) *m***/***z* [M + Na]^+^ calcd for C_19_H_20_N_2_ONa 315.1468, found 315.1475.

### Larger Scale Synthesis of Compound **4a** in *tert*-Amyl Alcohol

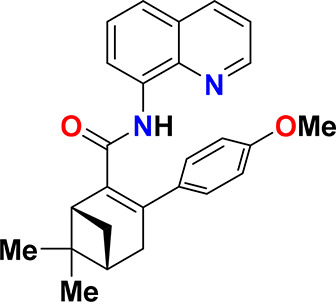



A capped vial equipped with a stirring bar was charged with substrate **3** (0.732 g, 2.5 mmol, 1.0 equiv), Pd(OAc)_2_ (28 mg, 0.125 mmol, 5 mol %), AgOAc (0.83 g, 5 mmol, 2.0 equiv), 4-iodoanisole (1.76 g, 7.5 mmol, 3.0 equiv), and NaOAc (0.205 g, 2.5 mmol, 1.0 equiv). All of the solids were then suspended in *tert*-amyl alcohol (5 mL), and the reaction vessel was evacuated and refilled with N_2_ before being placed in a preheated oil bath at 100 °C for the time given in Table 1. After completion of the reaction, the crude mixture was allowed to cool to rt. It was then diluted with EtOAc and filtered through a pad of Celite, and the filtrate was concentrated *in vacuo*. Purification by column chromatography (gradient from 0% to 10% EtOAc in pentane) afforded the desired product **4a** as a yellow amorphous solid (727 mg, 73% yield).

### Synthesis of Carboxylic Acid **5** by Hydrolytic Cleavage of the 8-AQ-Directing Group

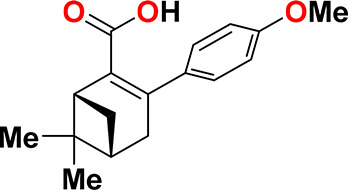



Compound **4a** (39 mg, 0.1 mmol, 1.0 equiv) and NaOH (60 mg, 1.5 mmol, 15.0 equiv) were mixed in EtOH (1.5 mL) in a capped vial and heated at 80 °C for 3 days. After each 24 h, additional NaOH (40 mg, 1.0 mmol, 10.0 equiv) was added to the reaction mixture. When the reaction had reached completion, the mixture was transferred to a separation funnel where it was diluted with 1 M aqueous NaOH (10 mL) and washed with DCM (2 × 10 mL). The aqueous layer was then acidified to pH 1 by the use of concentrated HCl. The acidic aqueous layer was extracted with CHCl_3_/i-PrOH (3:1 ratio, 3 × 15 mL), and the organic layers were subsequently combined, dried over Na_2_SO_4_, and concentrated *in vacuo*, to furnish the pure transacid as a brown solid (24.0 mg, 88% yield). (1*R*,5*R*)-3-(4-Methoxyphenyl)-6,6-dimethylbicyclo[3.1.1]hept-2-ene-2-carboxylic acid (**5**): ^1^H NMR (400 MHz, CDCl_3_) δ 7.18–7.16 (m, 2H), 6.87–6.85 (m, 2H), 3.81 (s, 3H), 2.87 (t, 1H, *J* = 5.74 Hz), 2.69 (dd, 1H, *J* = 19.0, 2.91 Hz), 2.59 (dd, 1H, *J* = 19.0, 2.60 Hz), 2.53–2.48 (m, 1H), 2.21–2.17 (m, 1H), 1.35 (s, 3H), 1.29 (d, 1H, *J* = 9.15 Hz), 0.93 (s, 3H) (proton of COOH not visible); ^13^C{^1^H} NMR (100 MHz, CDCl_3_) δ 171.6, 159.2, 147.7, 133.8, 132.9, 128.3, 113.6, 55.2, 43.2, 40.3, 39.8, 38.1, 31.4, 25.7, 21.0; HRMS (ESI) *m***/***z* [M + Na]^+^ calcd for C_17_H_20_O_3_Na 295.1305, found 295.1297.

### Synthesis of Cyclobutane Keto Acid **6** by Ozonolysis

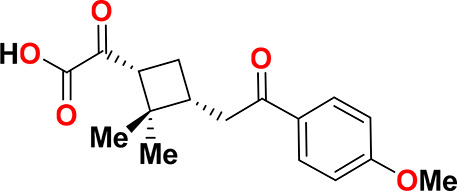



Compound **5** (27 mg, 0.1 mmol, 1 equiv) was dissolved in dry DCM (1 mL) and cooled to −78 °C. First, a stream of ozone was passed through the reaction solution for 5 min (until the characteristic blue color appeared), and then it was replaced by a stream of oxygen that was maintained until decolorization of the reaction solution occurred. Next, dimethyl sulfide (0.1 mL) was added, and the reaction mixture was then allowed to reach rt and stirred for 3 h. Once the reaction had reached completion, the reaction mixture was diluted with DCM (10 mL) and washed with H_2_O (3 × 10 mL). The organic layer was then filtered, dried over Na_2_SO_4_, and concentrated *in vacuo*. No further purification of the product was performed, and it was obtained as a yellow foamy solid (26 mg, purity of ≥90%, 77–83%): ^1^H NMR (400 MHz, CDCl_3_) δ 7.92–7.90 (m, 2H), 6.93 (m, 2H), 3.87 (s, 3H), 3.75–3.69 (m, 1H), 3.00–2.92 (m, 2H), 2.72–2.67 (m, 1H), 2.13–2.09 (m, 2H), 1.47 (s, 3H), 0.86 (m, 3H) (proton of COOH not visible); ^13^C{^1^H} NMR (100 MHz, CDCl_3_) δ 197.8, 195.3, 163.6, 160.1, 130.3, 129.9, 113.8, 55.5, 49.0, 46.5, 39.0, 38.2, 30.2, 22.5, 18.2; HRMS (ESI) *m***/***z* [M + Na]^+^ calcd for C_17_H_20_O_5_Na 327.1203, found 327.1201.

## Supporting Information Available

Raw NMR spectra (PDF)
