# Supplementary material for: A Study of an 8-Aminoquinoline-Directed C(sp2)–H Arylation Reaction on the Route to Chiral Cyclobutane Keto Acids from Myrtenal
Source: J Org Chem. 2021 May 27;86(12):8527–37. doi: 10.1021/acs.joc.1c00774 (PMC8279478; doi:10.1021/acs.joc.1c00774)
Supplement: Supplementary file 1 — jo1c00774_si_001.pdf [file jo1c00774_si_001.pdf]

Supporting Information for "A Study of an 8-Aminoquinoline Directed C(sp^2)-H Arylation Reaction on the Route to Chiral Cyclobutane Keto Acids from Myrtenal"

Monireh Pourghasemi Lati,*[a] Jonas Ståhle,[a] Michael Meyer,[a] Oscar Verho*[a,b]

[a]Department of Organic Chemistry, Arrhenius Laboratory, Stockholm University, SE-106 91 Stockholm, Sweden

[b]Department of Medicinal Chemistry, Uppsala Biomedical Centre, Uppsala University, SE-751 23 Uppsala, Sweden

Department of Organic Chemistry, Arrhenius Laboratory, Stockholm University, SE-106 91 Stockholm, Sweden. E-mail: oscar.verho@ilk.uu.se, monireh.lati@su.se

Table of Content

Raw NMR spectra.. **S3**

Compound **2**.. **S3**

Compound **3**.. **S4**

C–H arylated compounds **4a–u** **S5**

Compound **5**.. **S26**

Compound **6**.. **S27**

Raw NMR spectra

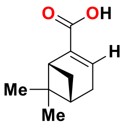

2

^{1}H NMR (400 MHz, $CDCl_3$)

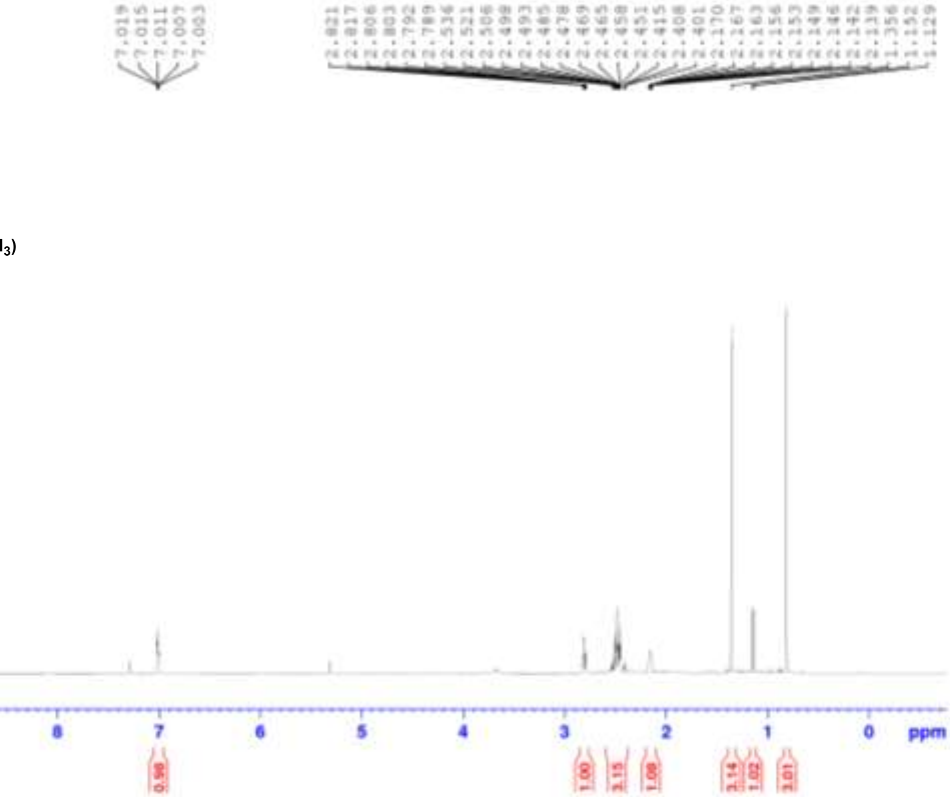

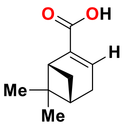

2

^{13}C{^{1}H} NMR (100 MHz, $CDCl_3$)

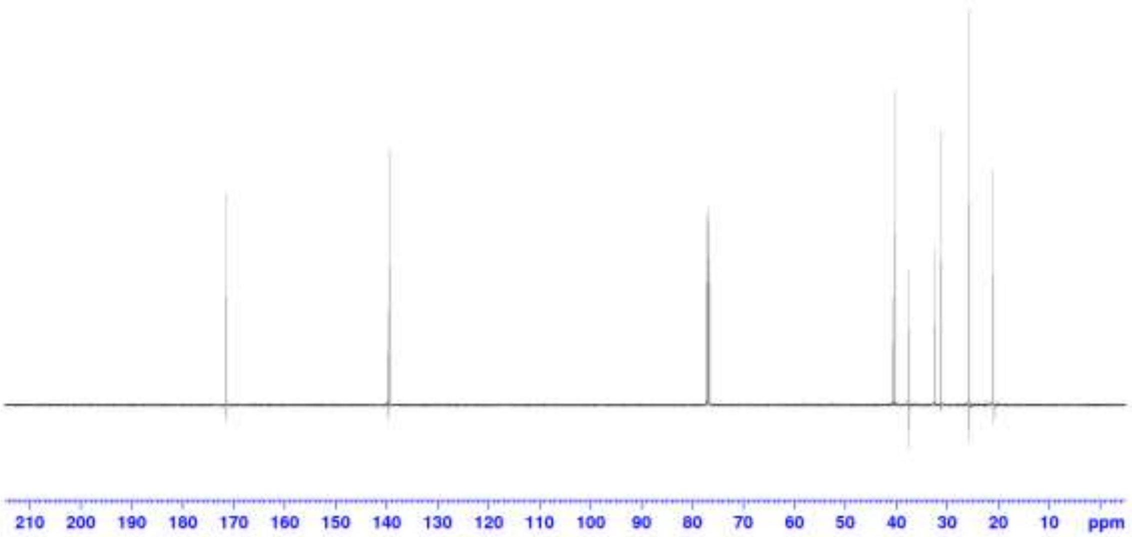

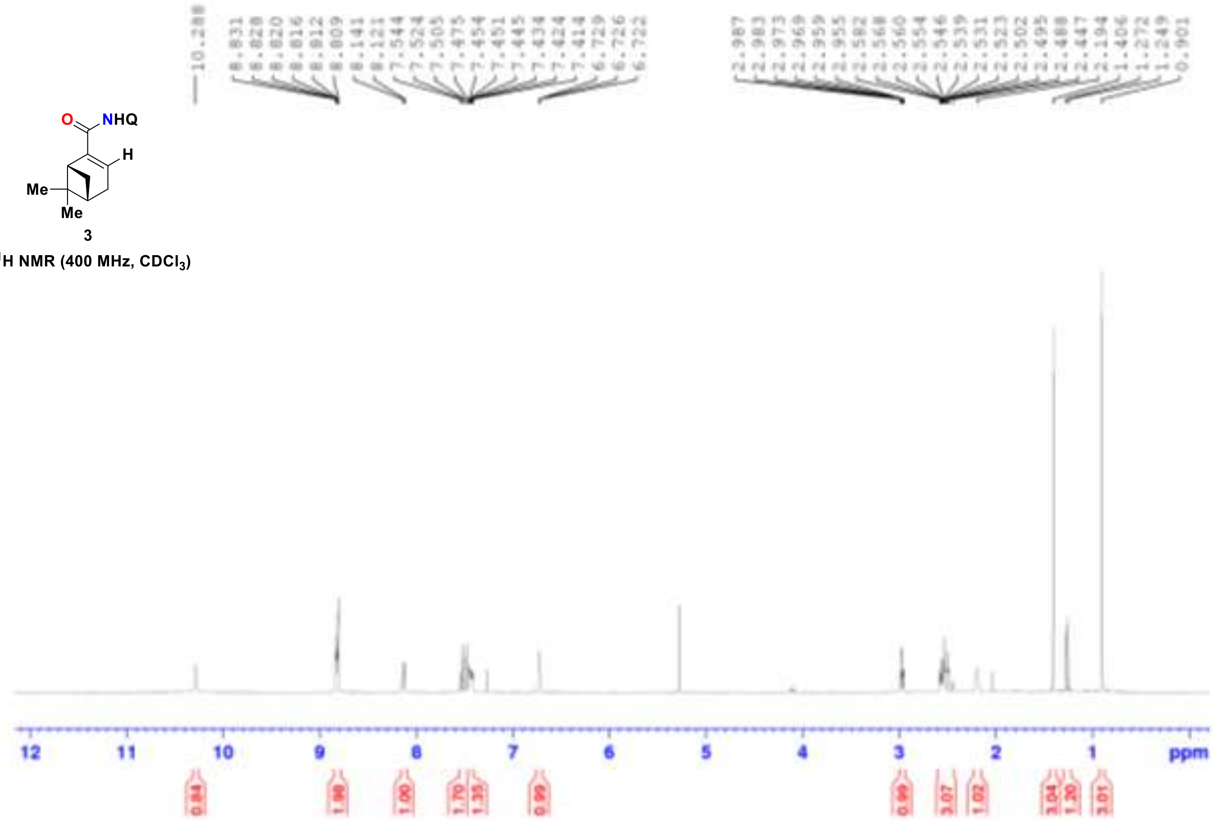

3

^{1}H NMR (400 MHz, $CDCl_3$)

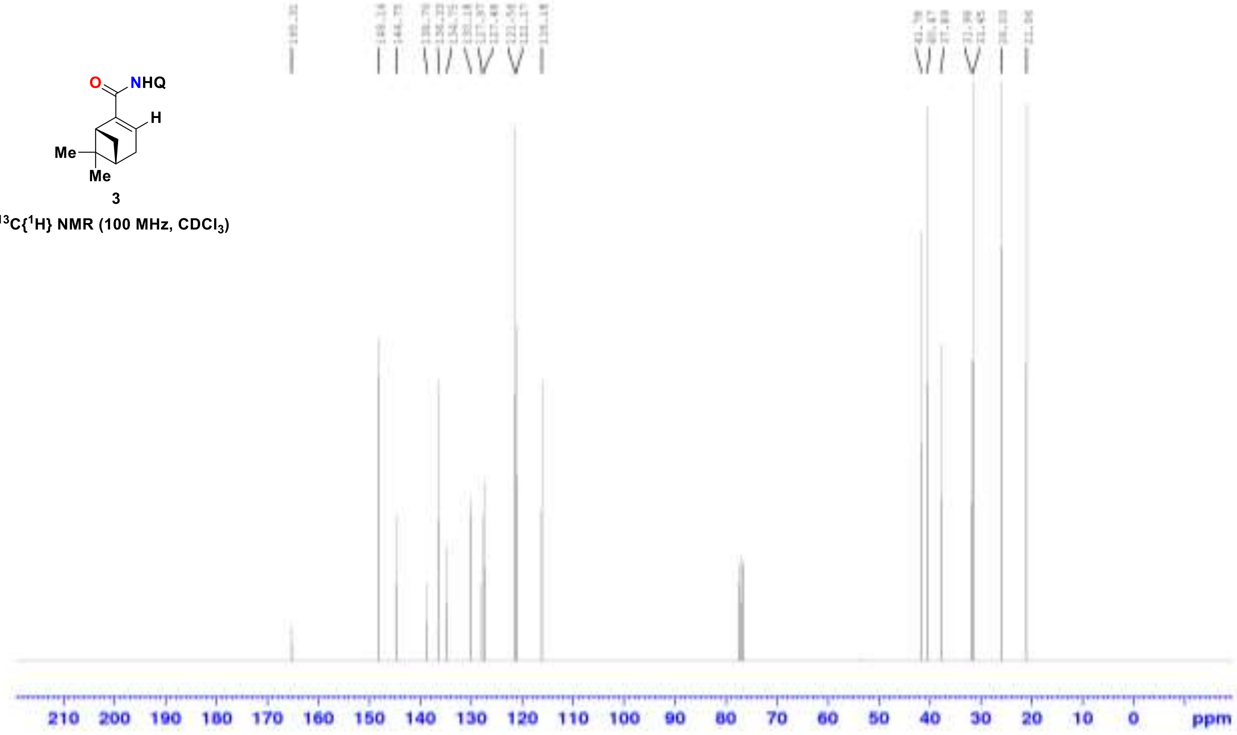

3

$^{13}C\{^{1}H\}$ NMR (100 MHz, $CDCl_3$)

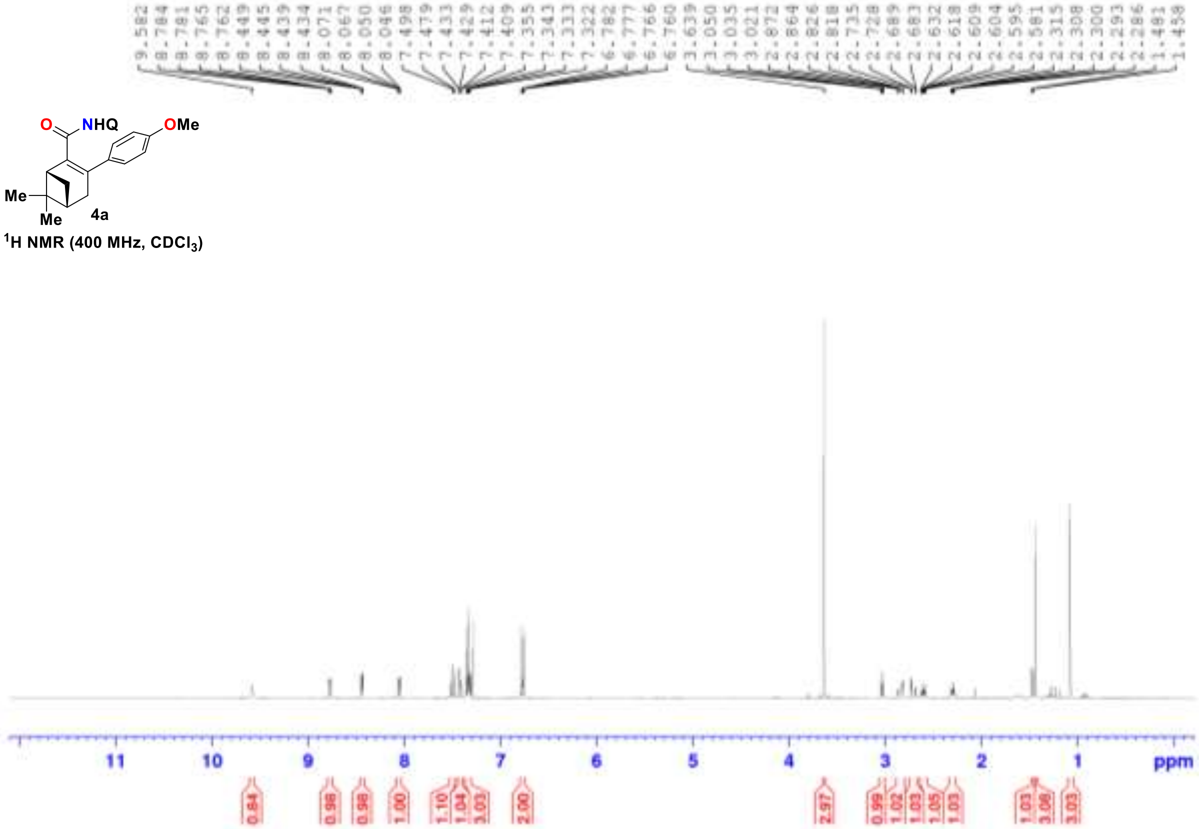

^{1}H NMR (400 MHz, $CDCl_3$)

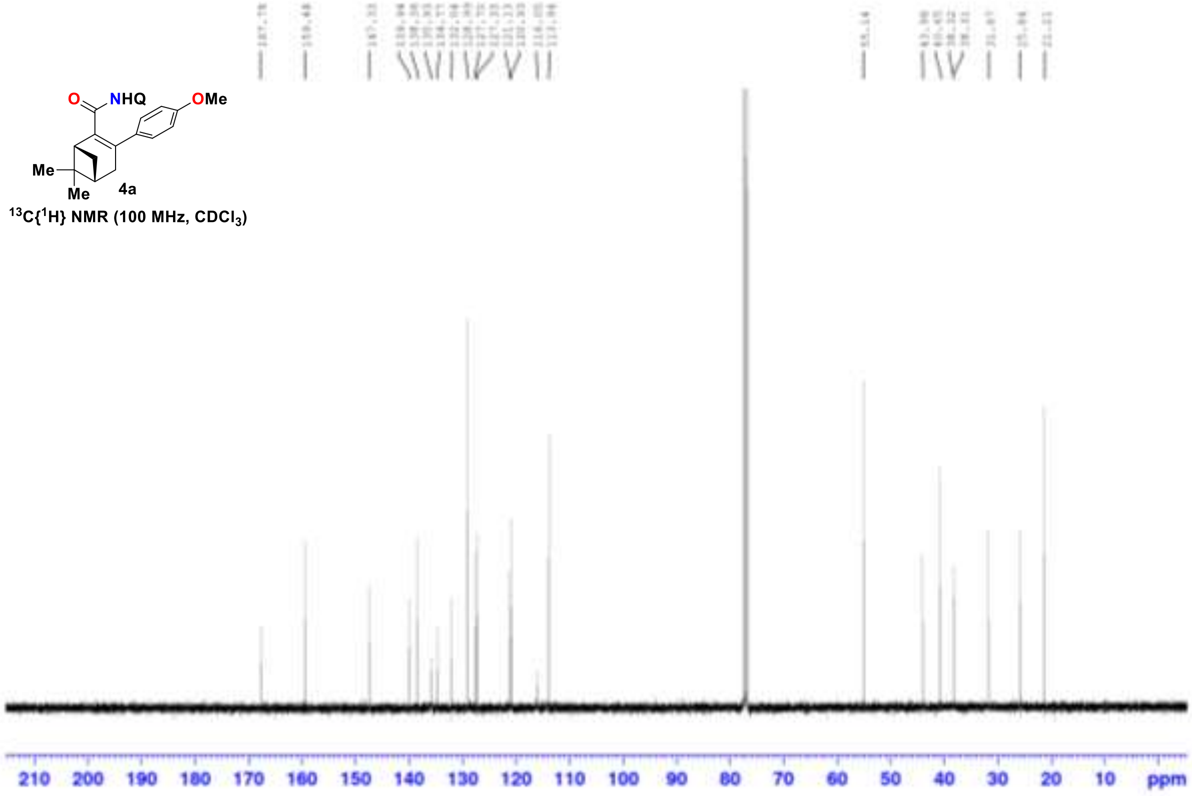

^{13}C{^{1}H} NMR (100 MHz, $CDCl_3$)

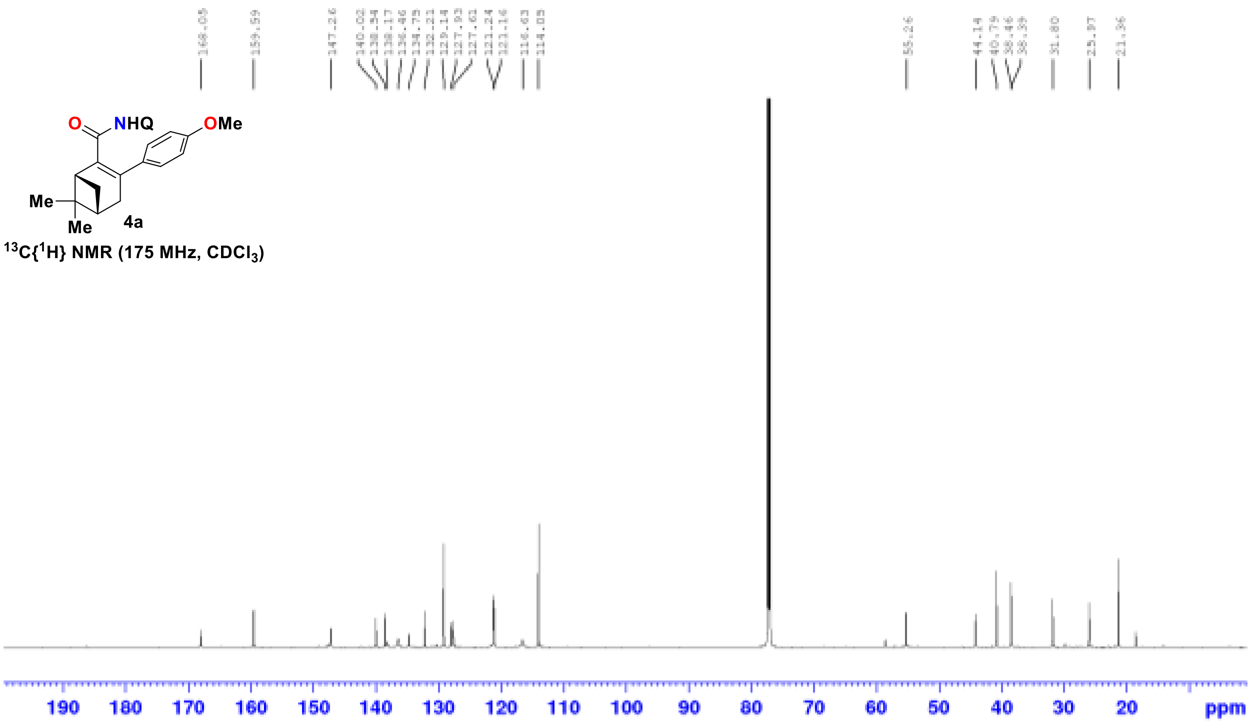
168.05
159.59
147.26
140.02
138.34
138.17
136.46
134.75
132.21
129.14
127.93
127.61
121.24
121.16
116.63
114.05
55.26
44.14
40.79
38.46
38.39
31.80
25.97
21.36
O
NHQ
OMe
Me
Me
4a
$^{13}C\{^{1}H\}$ NMR (175 MHz, $CDCl_3$)
190
180
170
160
150
140
130
120
110
100
90
80
70
60
50
40
30
20
ppm

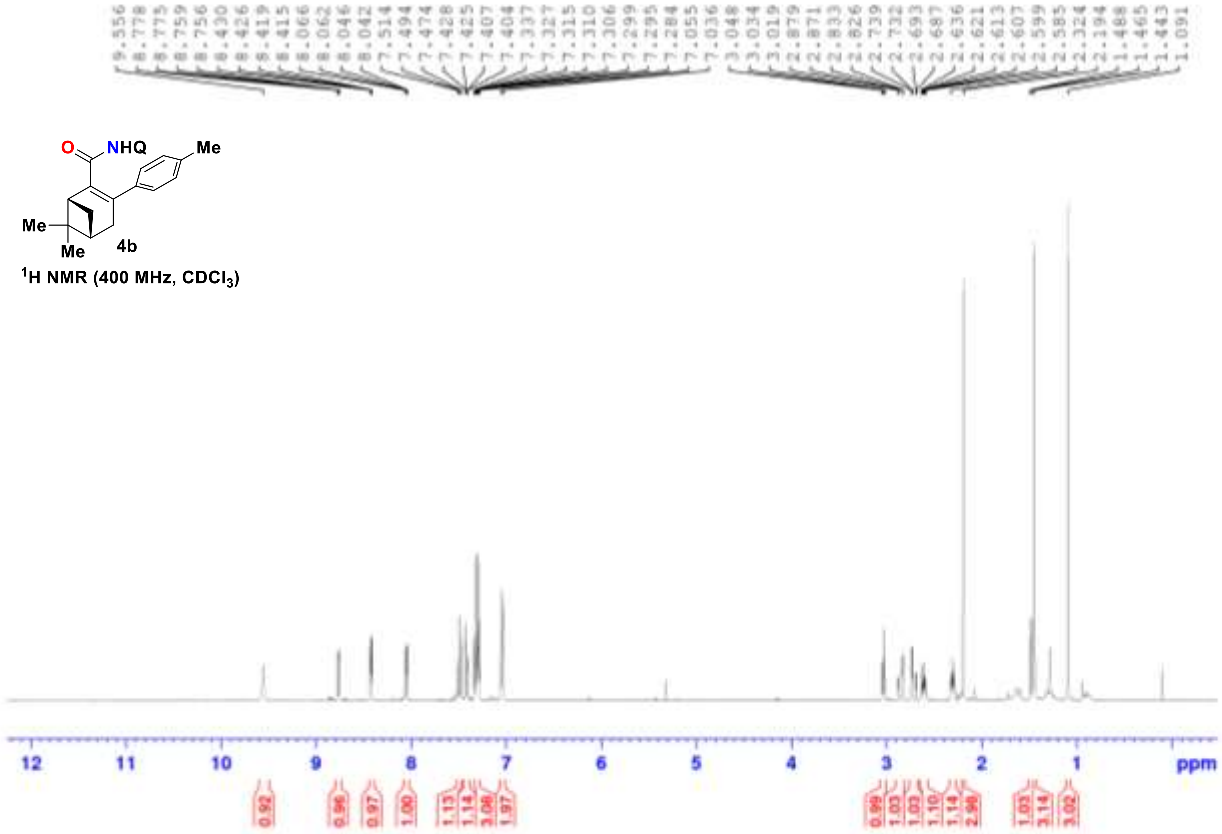
9.556
8.778
8.775
8.759
8.756
8.430
8.426
8.419
8.415
8.066
8.062
8.046
8.042
7.514
7.494
7.474
7.428
7.425
7.407
7.404
7.337
7.327
7.315
7.310
7.306
7.299
7.295
7.284
7.055
7.036
3.048
3.034
3.019
2.879
2.871
2.833
2.826
2.739
2.732
2.693
2.687
2.636
2.621
2.613
2.607
2.599
2.585
2.324
2.194
1.488
1.465
1.443
1.091
O
NHQ
Me
Me
Me
4b
1H NMR (400 MHz, CDCl3)
12 11 10 9 8 7 6 5 4 3 2 1 ppm
0.92
0.96
0.97
1.00
1.13
1.14
3.08
1.97
0.99
1.03
1.03
1.10
1.14
2.98
1.03
3.14
3.02

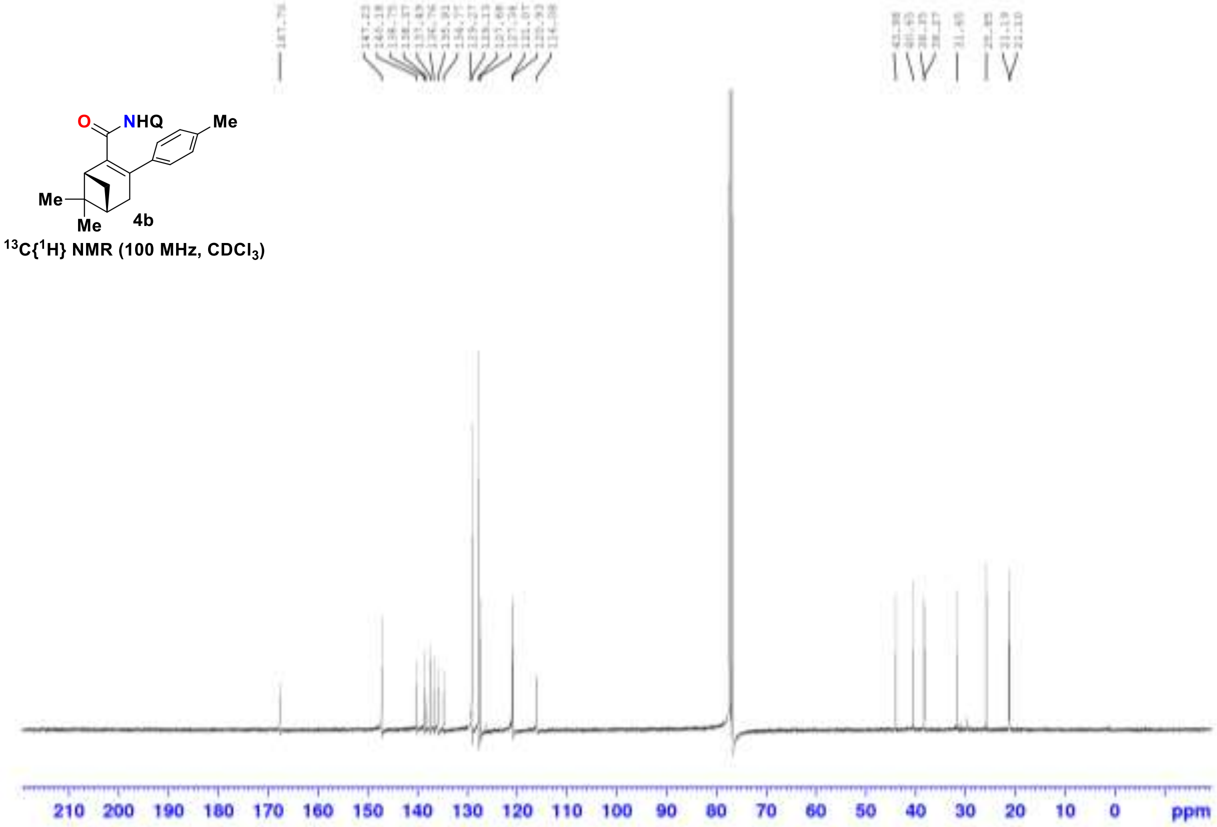
O
NHQ
Me
Me
Me
4b
13C{1H} NMR (100 MHz, CDCl3)
210 200 190 180 170 160 150 140 130 120 110 100 90 80 70 60 50 40 30 20 10 0 ppm

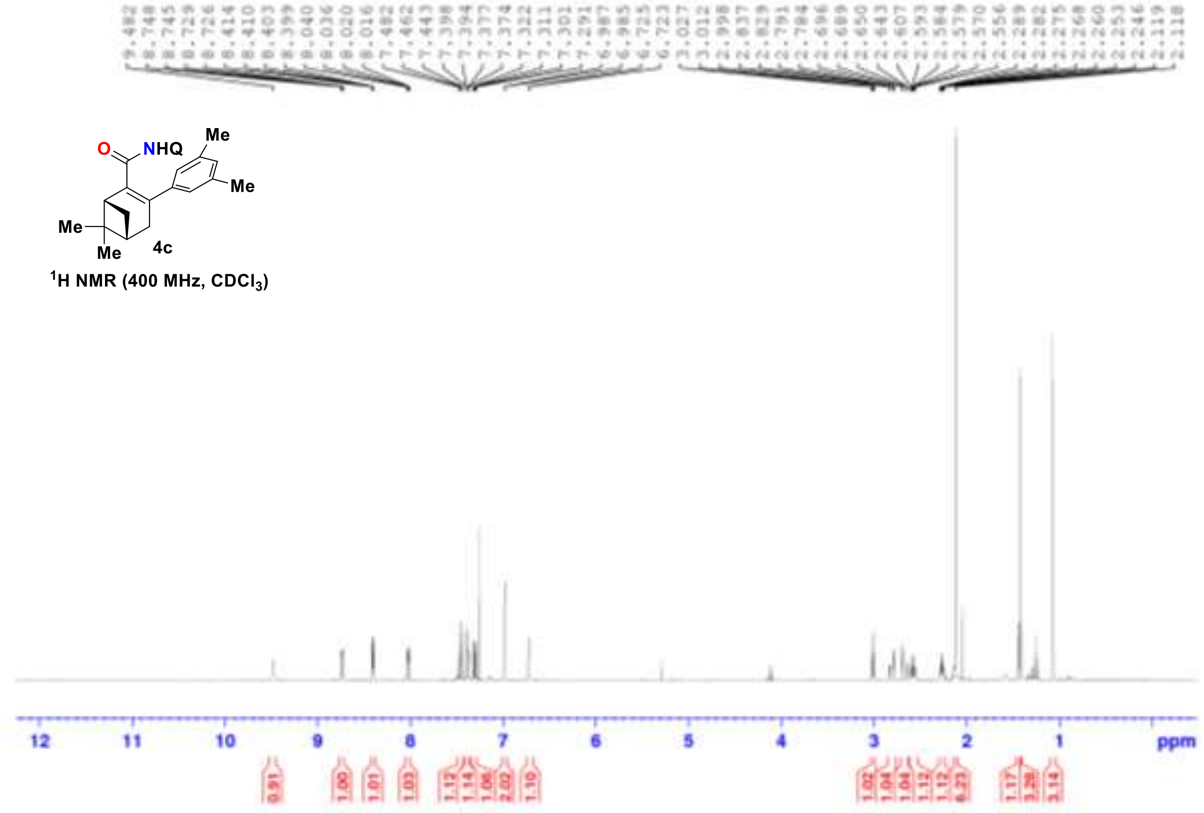
O
NHQ
Me
Me
Me
Me
4c
1H NMR (400 MHz, CDCl3)
ppm

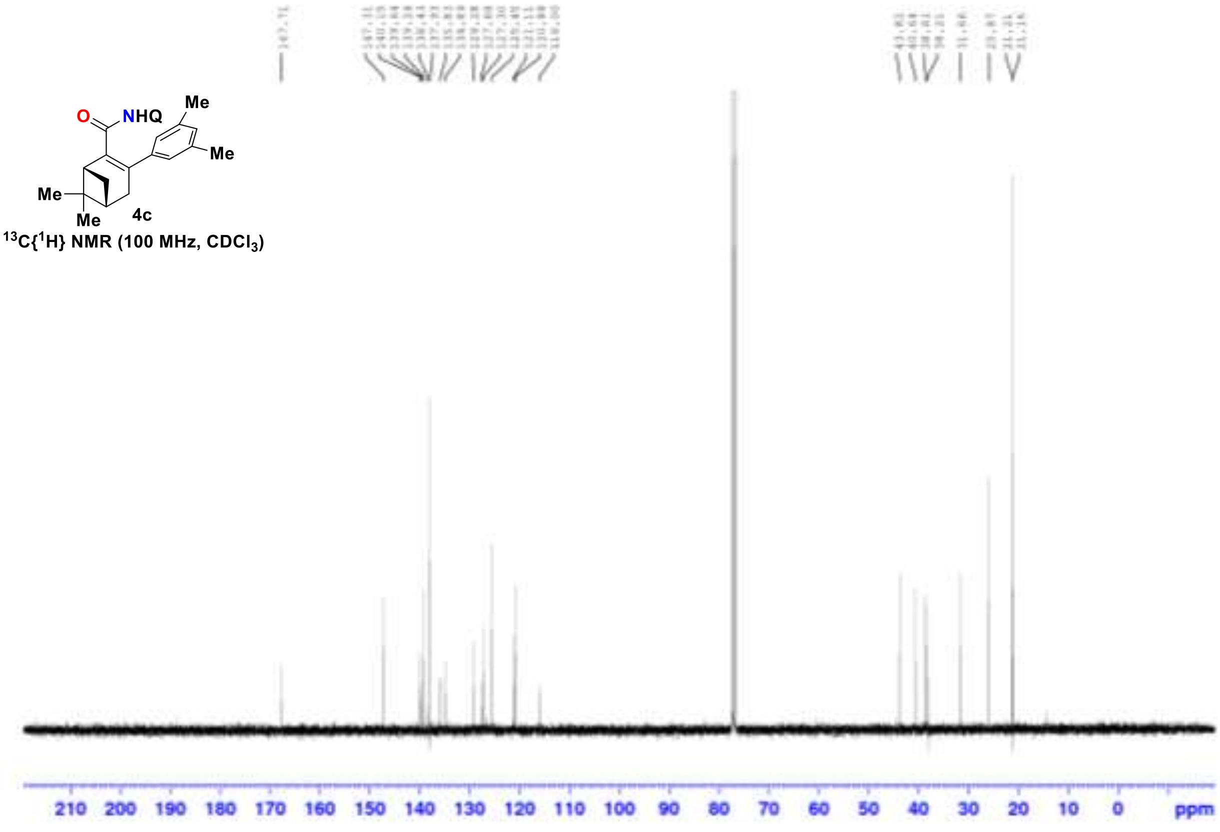
O
NHQ
Me
Me
Me
Me
4c
13C{1H} NMR (100 MHz, CDCl3)
ppm

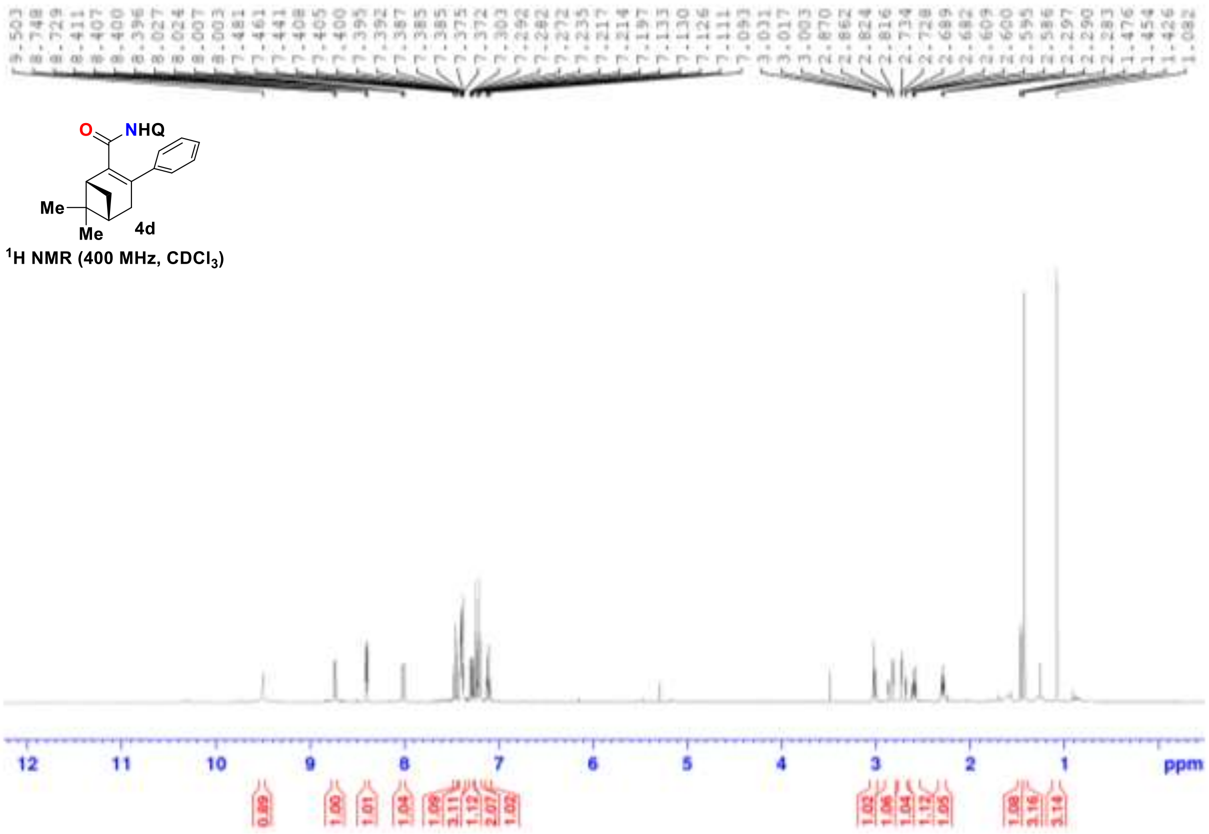
O
NHQ
Me
Me
4d
1H NMR (400 MHz, CDCl3)
12
11
10
9
8
7
6
5
4
3
2
1
ppm

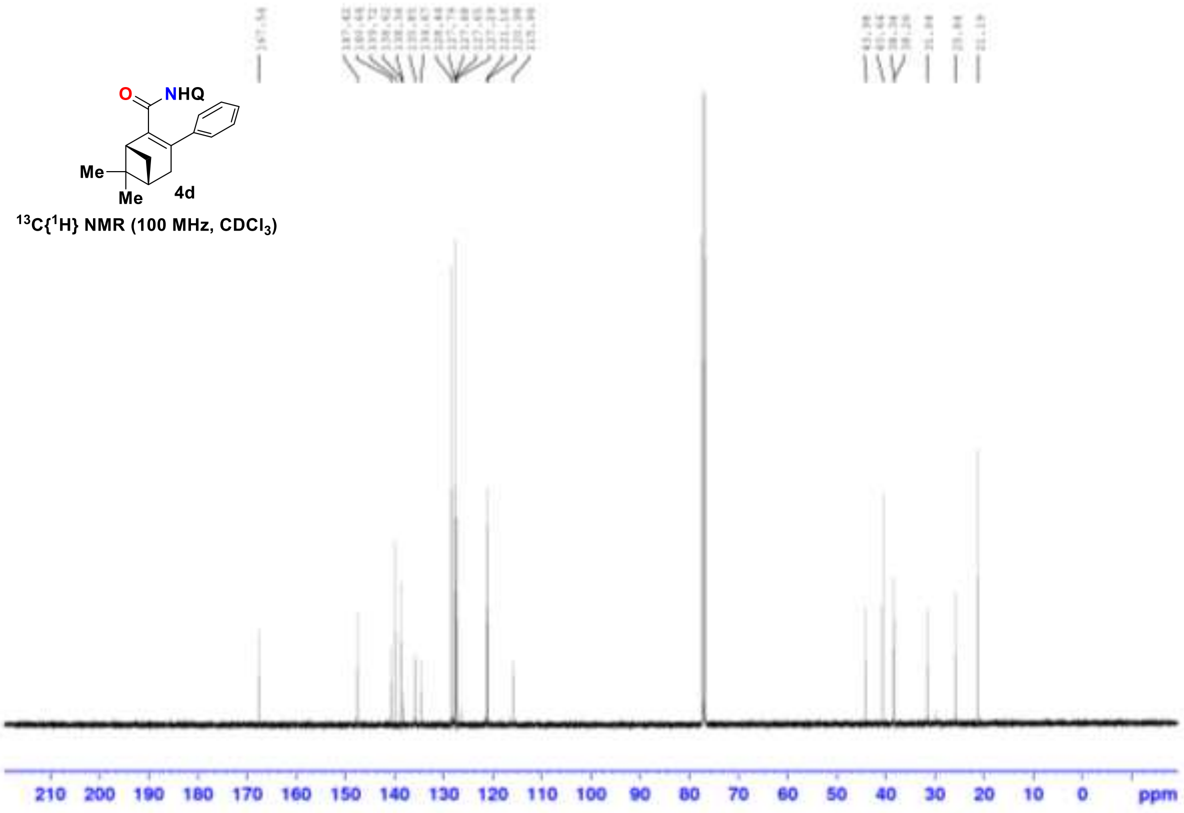
O
NHQ
Me
Me
4d
13C{1H} NMR (100 MHz, CDCl3)
210
200
190
180
170
160
150
140
130
120
110
100
90
80
70
60
50
40
30
20
10
0
ppm

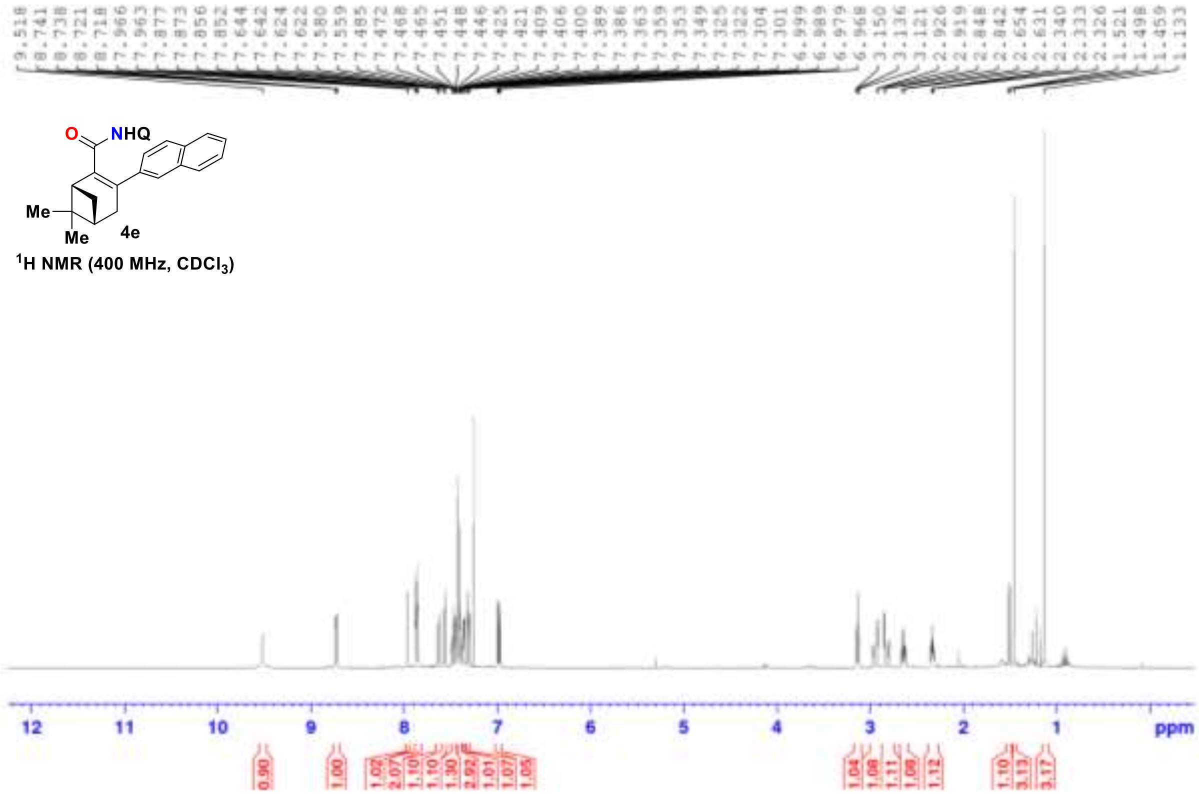
O
NHQ
Me
Me
4e
1H NMR (400 MHz, CDCl3)
ppm

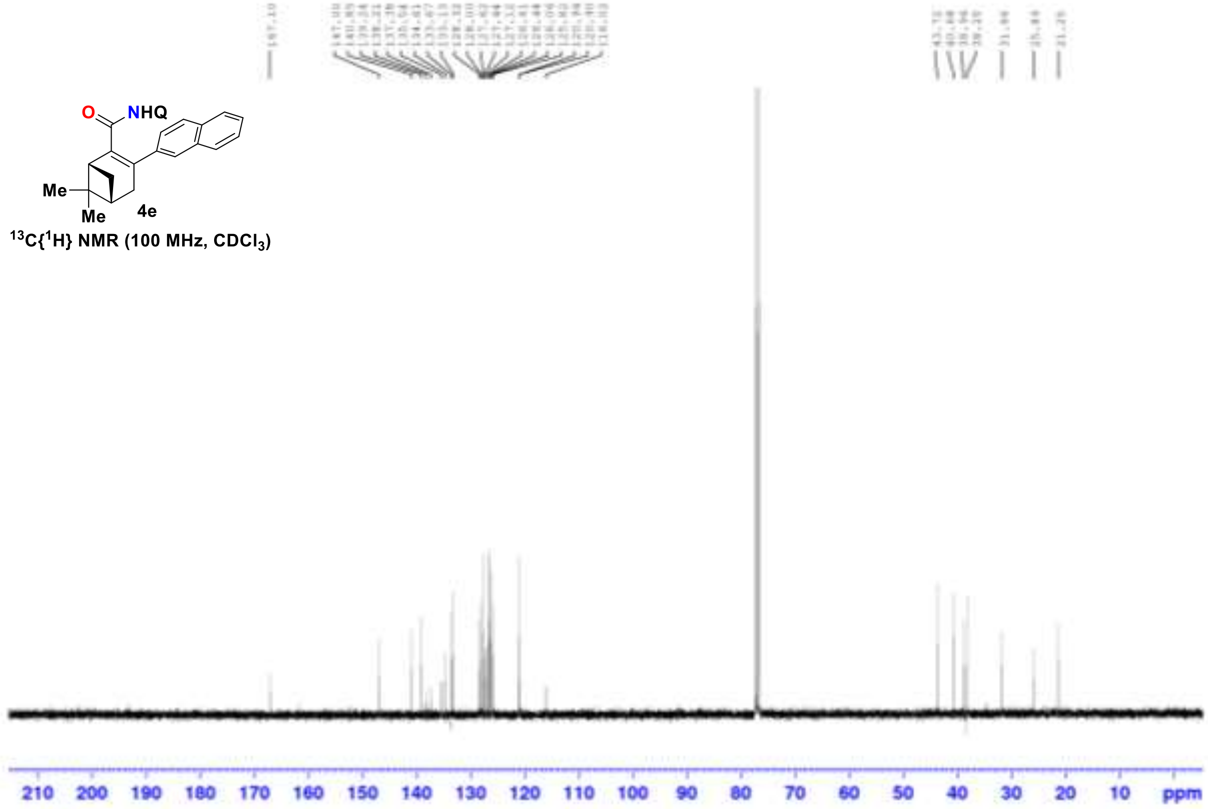
O
NHQ
Me
Me
4e
13C{1H} NMR (100 MHz, CDCl3)
ppm

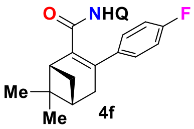

4f

^{1}H NMR (400 MHz, $CDCl_3$)

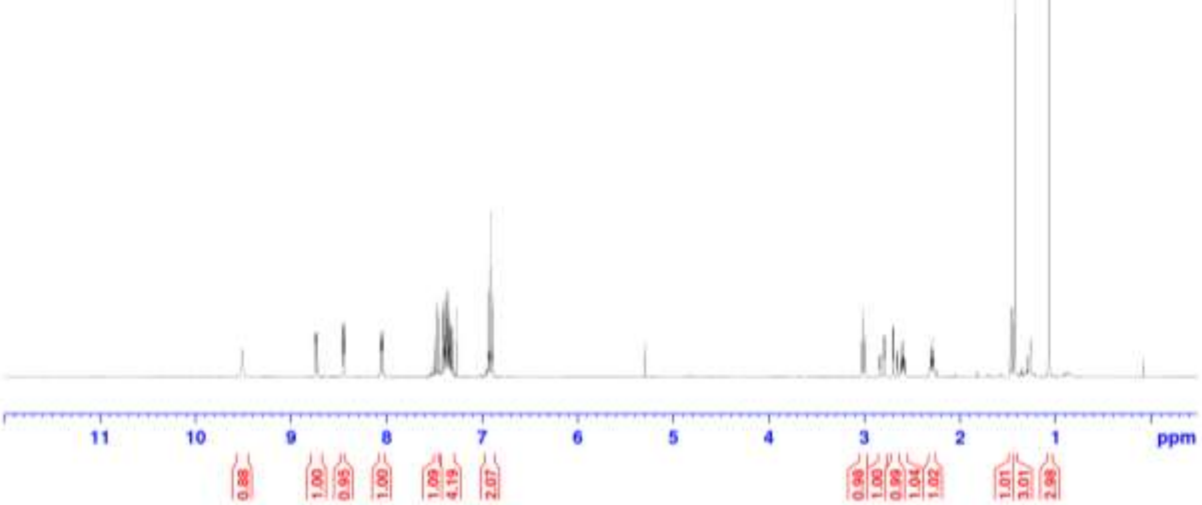

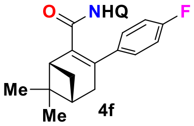

4f

$^{13}C\{^{1}H\}$ NMR (100 MHz, $CDCl_3$)

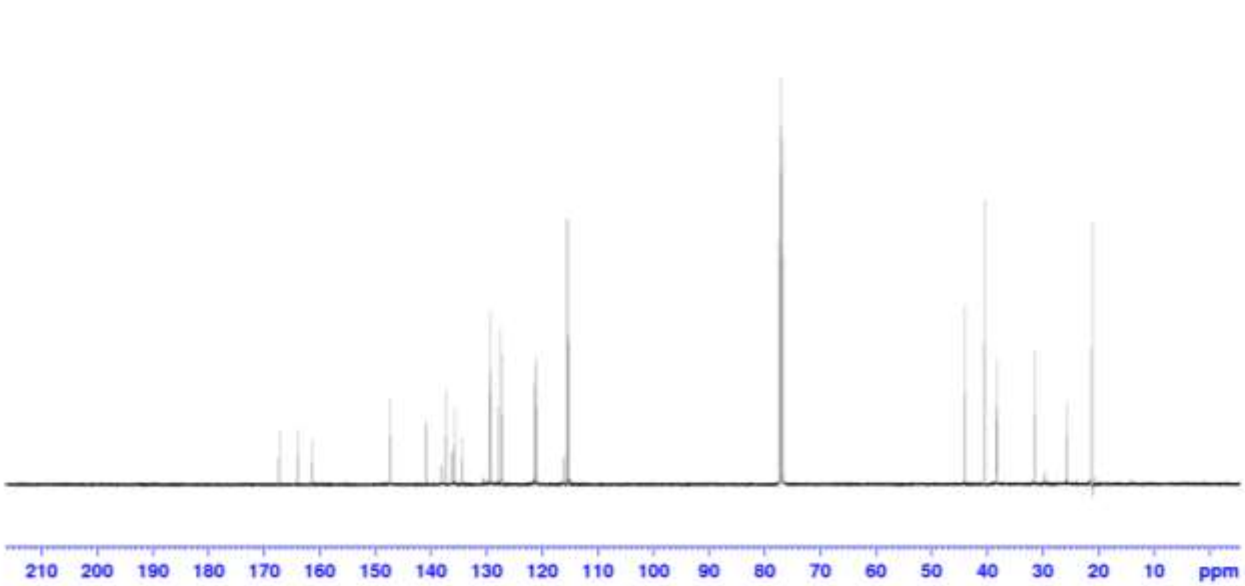

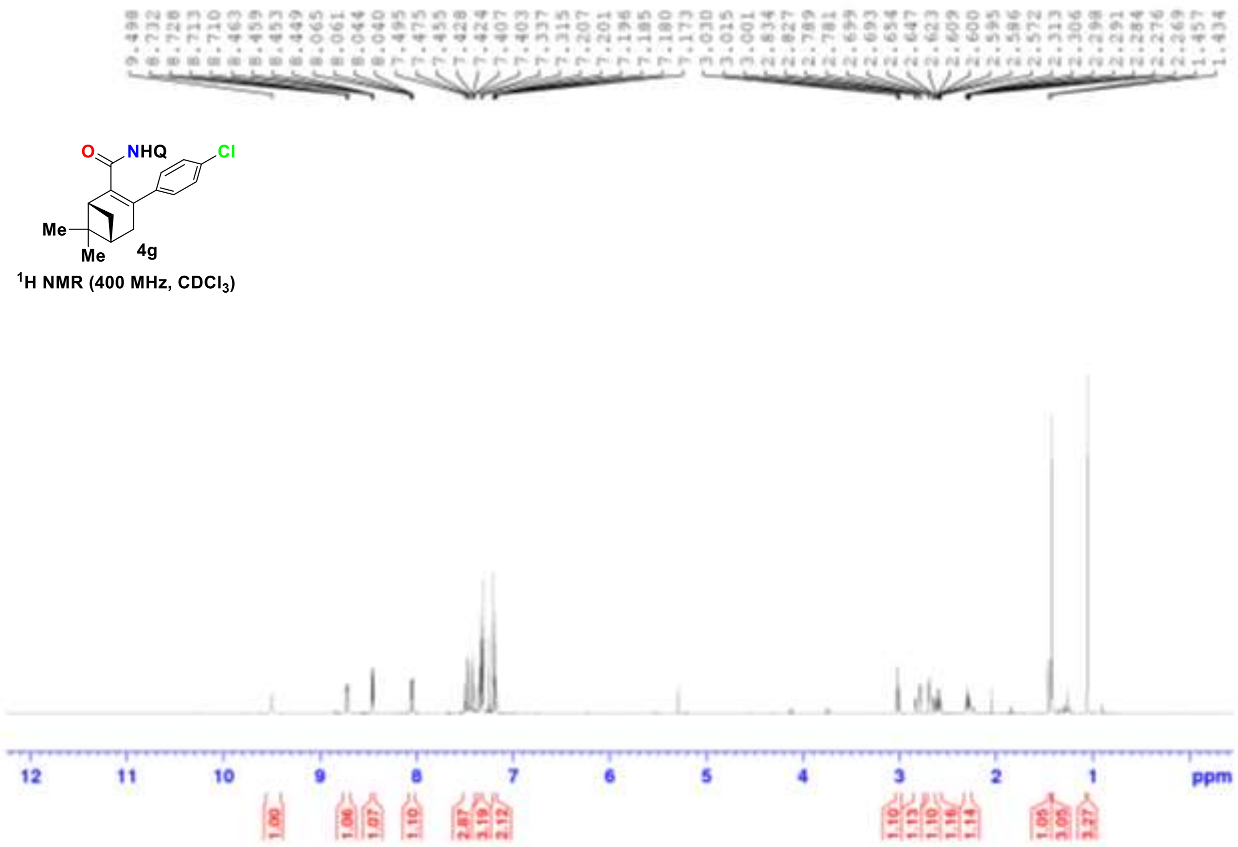

^{1}H NMR (400 MHz, $CDCl_3$)

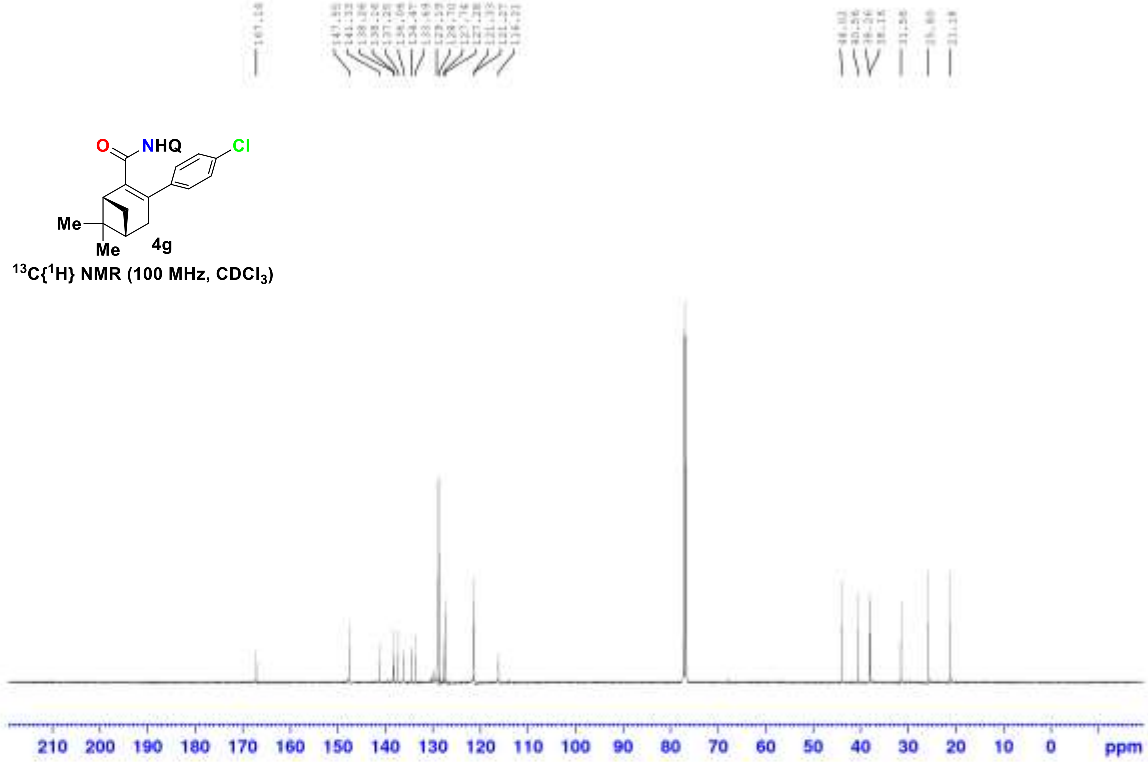

$^{13}C\{^1H\}$ NMR (100 MHz, $CDCl_3$)

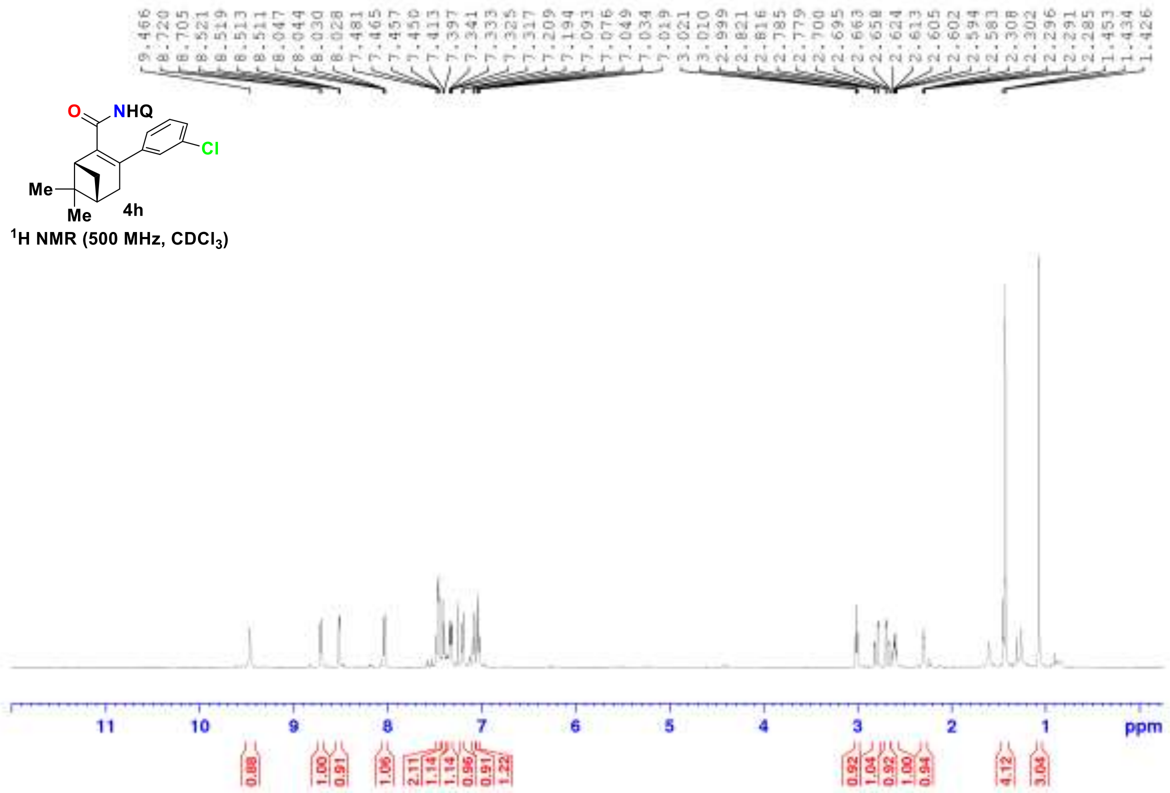
9.466
8.720
8.705
8.521
8.519
8.513
8.511
8.047
8.044
8.030
8.028
7.481
7.465
7.457
7.450
7.413
7.397
7.341
7.333
7.325
7.317
7.209
7.194
7.093
7.076
7.049
7.034
7.019
3.021
3.010
2.999
2.821
2.816
2.785
2.779
2.700
2.695
2.663
2.658
2.624
2.613
2.605
2.602
2.594
2.583
2.308
2.302
2.296
2.291
2.285
1.453
1.434
1.426
O
NHQ
Cl
Me
Me
4h
^{1}H NMR (500 MHz, $CDCl_3$)
11
10
9
8
7
6
5
4
3
2
1
ppm
0.88
1.00
0.91
1.06
2.11
1.14
1.14
0.96
0.91
1.22
0.92
1.04
0.92
1.00
0.94
4.12
3.04

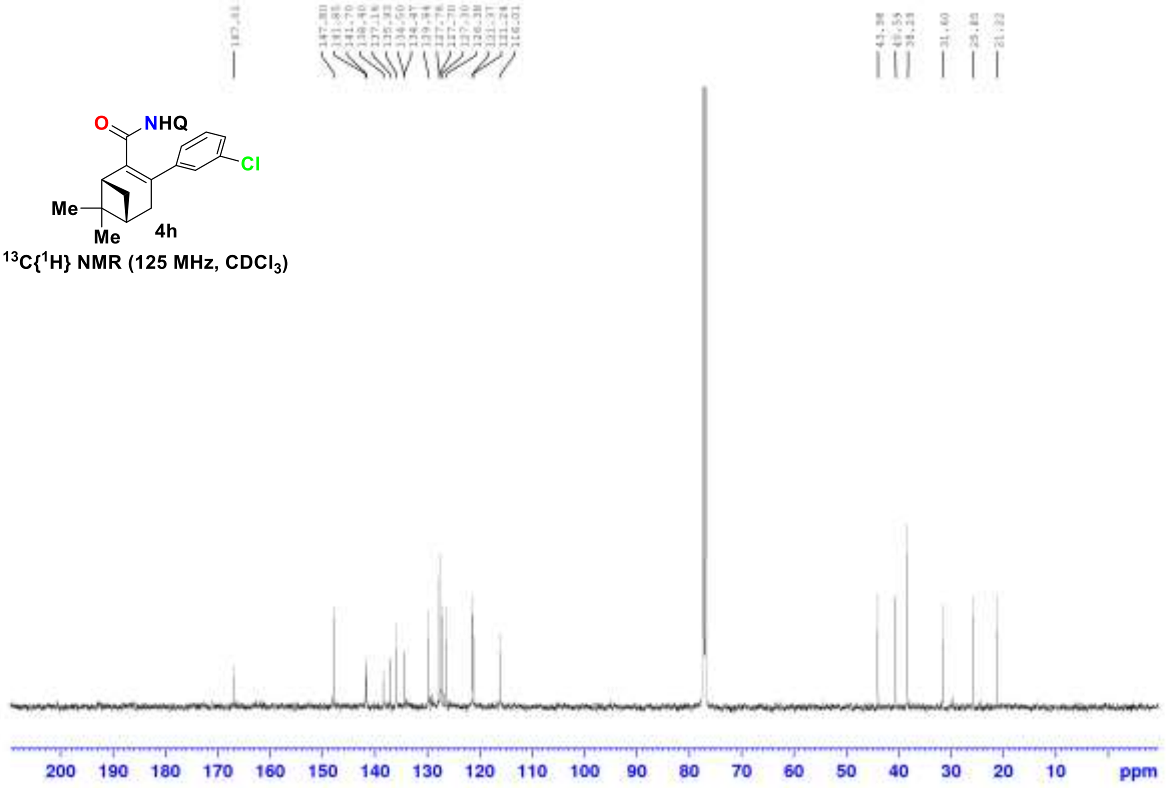
O
NHQ
Cl
Me
Me
4h
$^{13}C\{^{1}H\}$ NMR (125 MHz, $CDCl_3$)
200
190
180
170
160
150
140
130
120
110
100
90
80
70
60
50
40
30
20
10
ppm

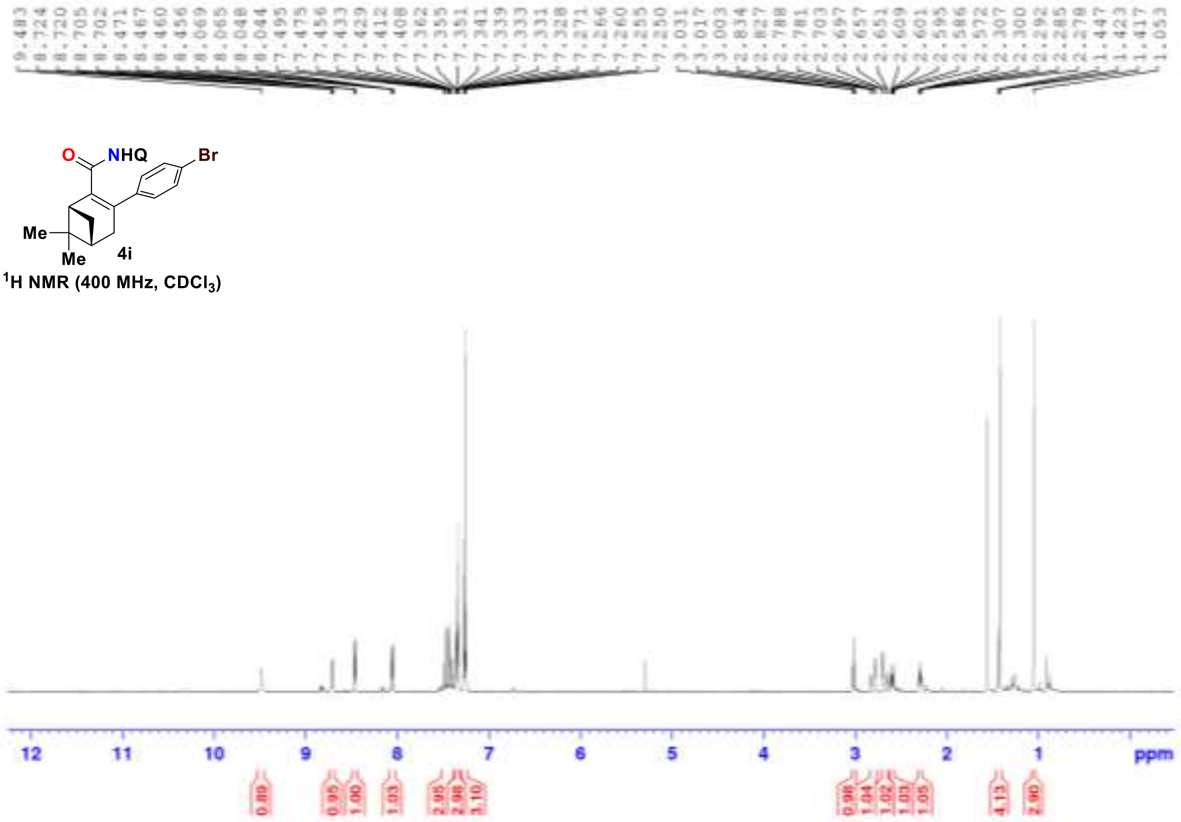
9.483
8.724
8.720
8.705
8.702
8.471
8.467
8.460
8.456
8.069
8.065
8.048
8.044
7.495
7.475
7.456
7.433
7.429
7.412
7.408
7.362
7.355
7.351
7.341
7.339
7.333
7.331
7.328
7.271
7.266
7.260
7.255
7.250
3.031
3.017
3.003
2.834
2.827
2.788
2.781
2.703
2.697
2.657
2.651
2.609
2.601
2.595
2.586
2.572
2.307
2.300
2.292
2.285
2.279
1.447
1.423
1.417
1.053
O
NHQ
Br
Me
Me
4i
^{1}H NMR (400 MHz, $CDCl_3$)
12
11
10
9
8
7
6
5
4
3
2
1
ppm
0.80
0.95
1.00
1.03
2.95
2.98
3.10
0.98
1.04
1.02
1.03
1.05
4.13
2.90

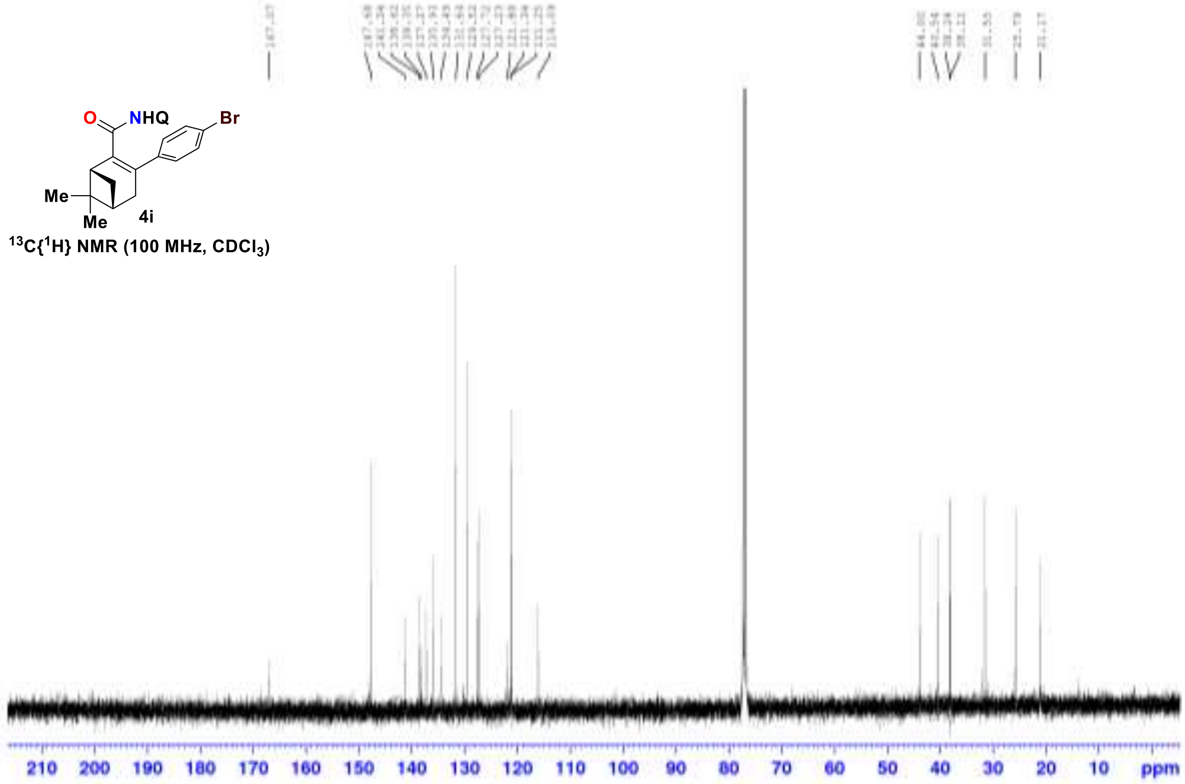
O
NHQ
Br
Me
Me
4i
$^{13}C\{^{1}H\}$ NMR (100 MHz, $CDCl_3$)
210
200
190
180
170
160
150
140
130
120
110
100
90
80
70
60
50
40
30
20
10
ppm

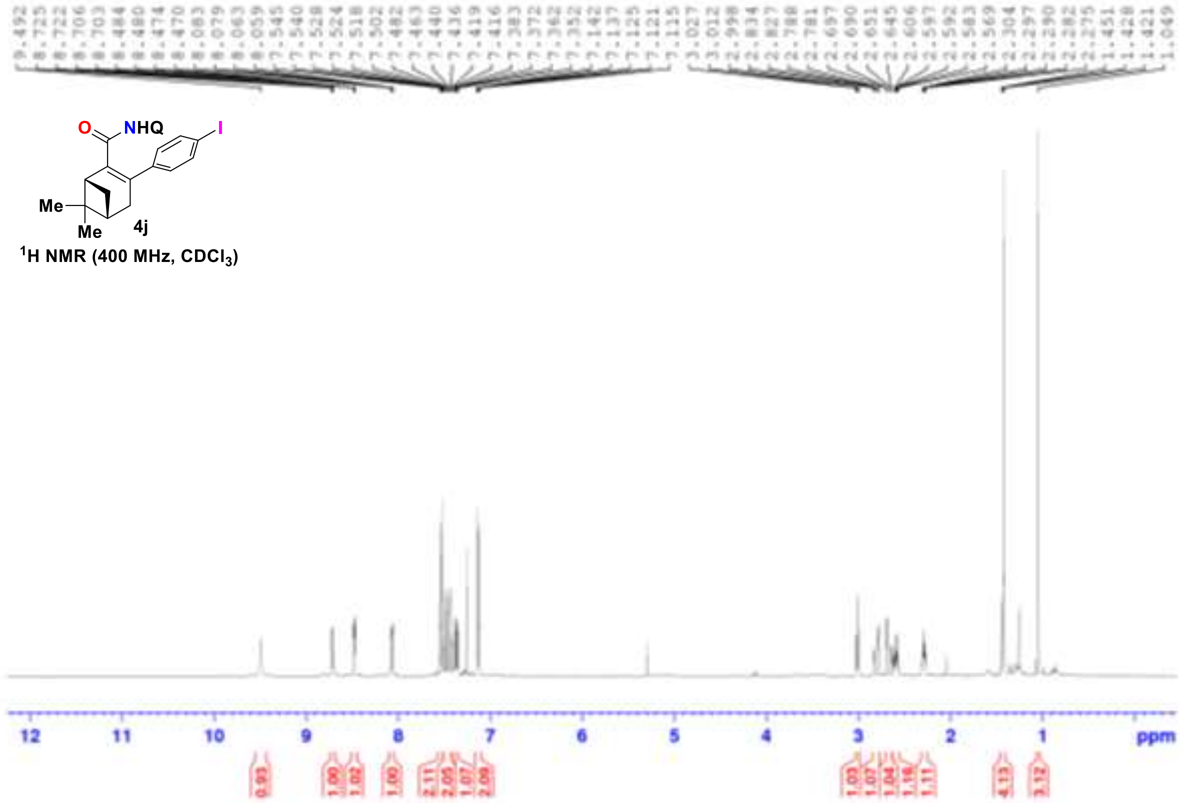
9.492
8.725
8.722
8.706
8.703
8.484
8.480
8.474
8.470
8.083
8.079
8.063
8.059
7.545
7.540
7.528
7.524
7.518
7.502
7.482
7.463
7.440
7.436
7.419
7.416
7.383
7.372
7.362
7.352
7.142
7.137
7.125
7.121
7.115
3.027
3.012
2.998
2.834
2.827
2.788
2.781
2.697
2.690
2.651
2.645
2.606
2.597
2.592
2.583
2.569
2.304
2.297
2.290
2.282
2.275
1.451
1.428
1.421
1.049
O
NHQ
I
Me
Me
4j
¹H NMR (400 MHz, CDCl₃)
12 11 10 9 8 7 6 5 4 3 2 1 ppm
0.93
1.00
1.02
1.00
2.11
2.05
1.07
2.09
1.03
1.07
1.04
1.16
1.11
4.13
3.12

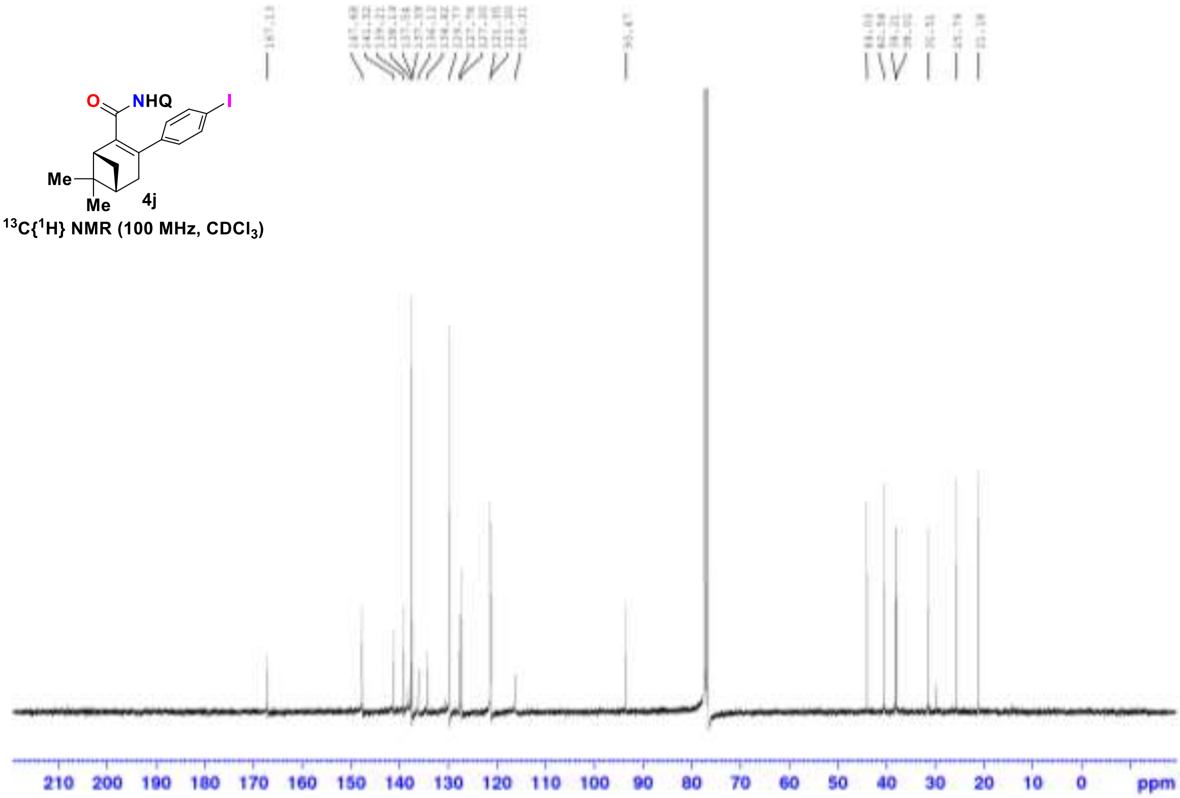
O
NHQ
I
Me
Me
4j
¹³C{¹H} NMR (100 MHz, CDCl₃)
210 200 190 180 170 160 150 140 130 120 110 100 90 80 70 60 50 40 30 20 10 0 ppm

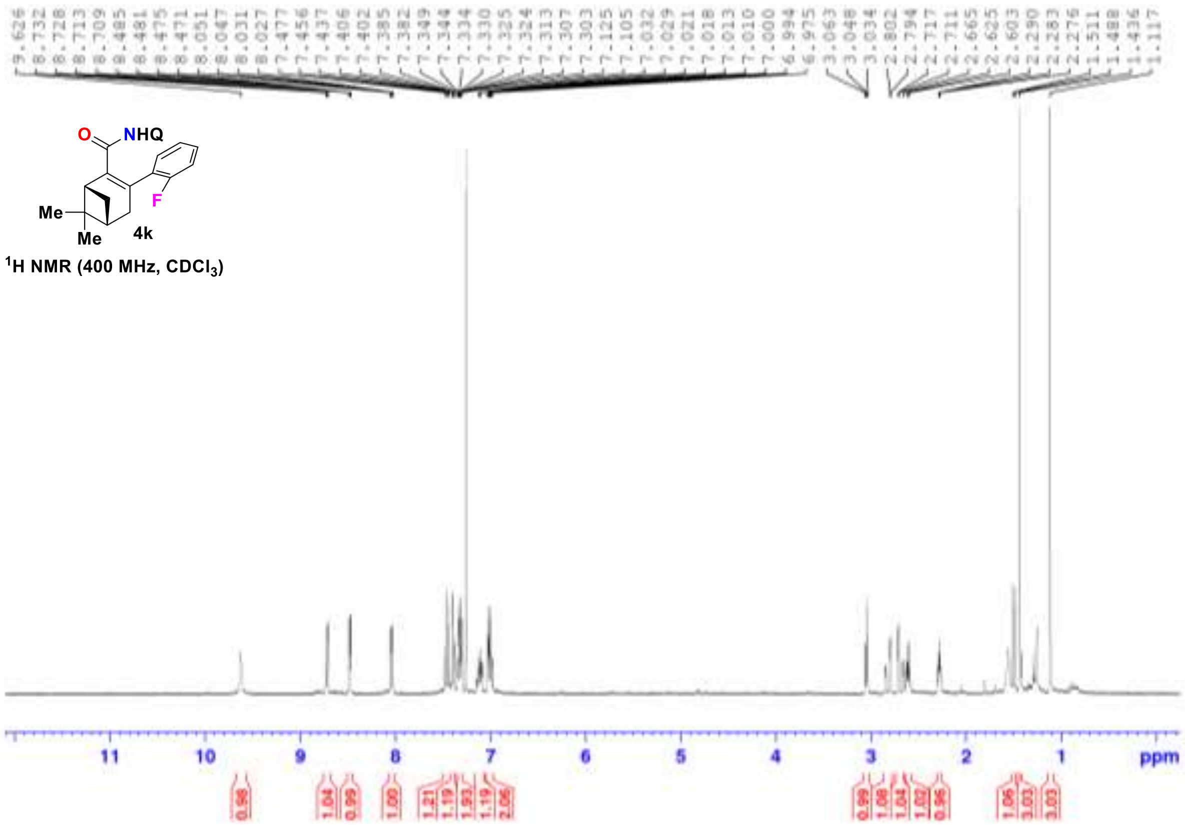
O
NHQ
F
Me
Me
4k
^{1}H NMR (400 MHz, CDCl$_3$)
ppm

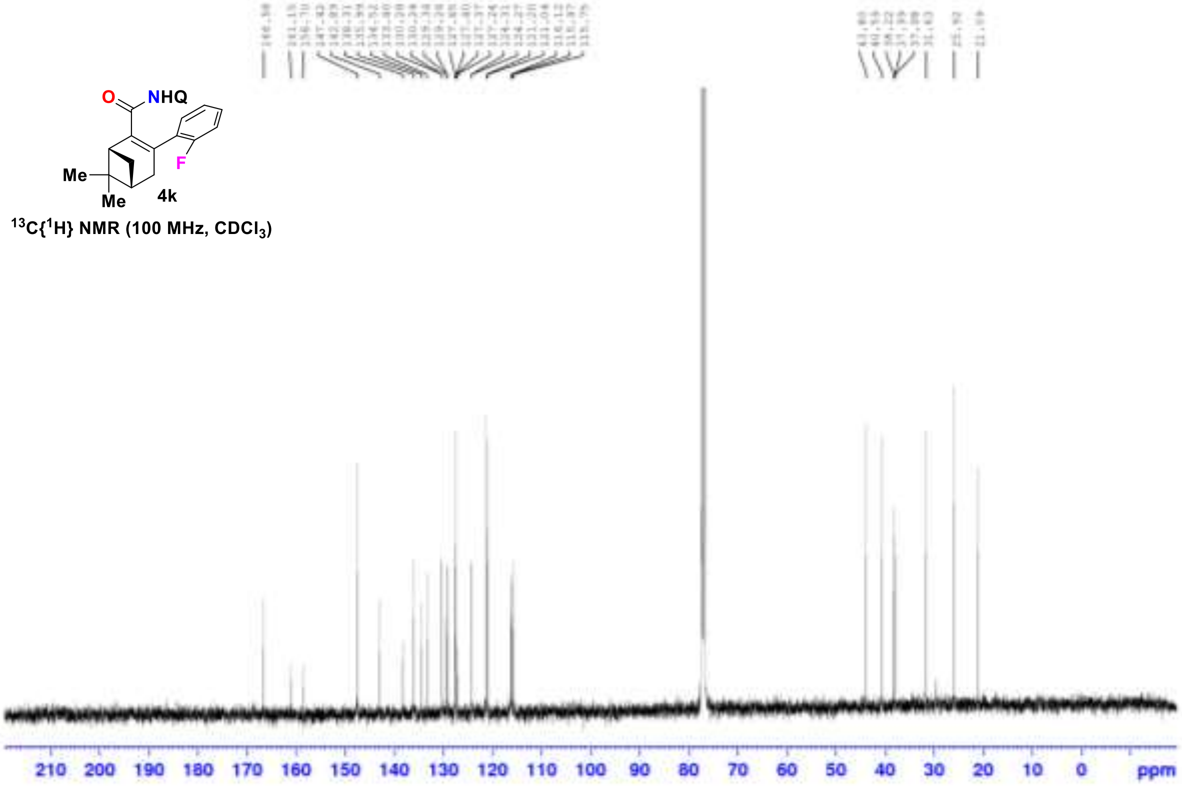
O
NHQ
F
Me
Me
4k
^{13}C{^{1}H} NMR (100 MHz, CDCl$_3$)
ppm

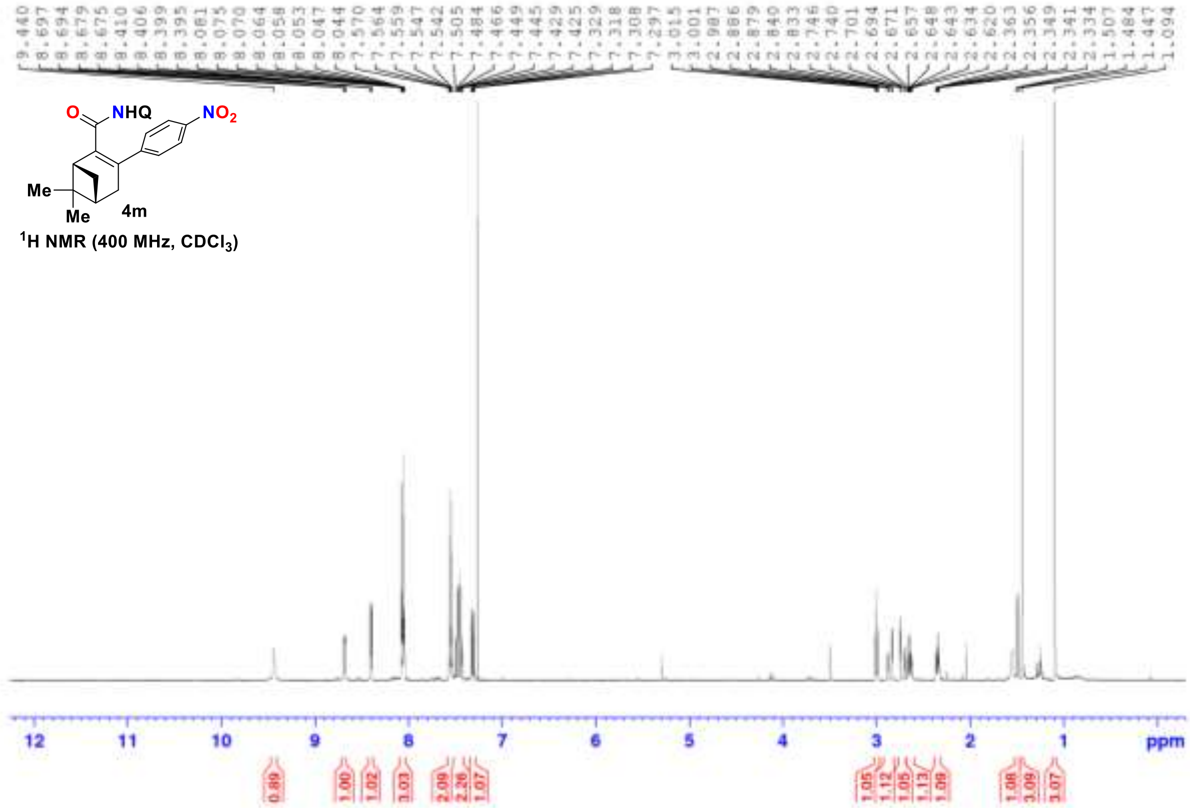
9.440 8.697 8.694 8.679 8.675 8.410 8.406 8.399 8.395 8.081 8.075 8.070 8.064 8.058 8.053 8.047 8.044 7.570 7.564 7.559 7.547 7.542 7.505 7.484 7.466 7.449 7.445 7.429 7.425 7.329 7.318 7.308 7.297 3.015 3.001 2.987 2.886 2.879 2.840 2.833 2.746 2.740 2.701 2.694 2.671 2.657 2.648 2.643 2.634 2.620 2.363 2.356 2.349 2.341 2.334 1.507 1.484 1.447 1.094
O
NHQ
NO2
Me
Me
4m
1H NMR (400 MHz, CDCl3)
ppm
0.89 1.00 1.02 3.03 2.09 2.26 1.07 1.05 1.12 1.05 1.13 1.08 1.08 3.09 3.07

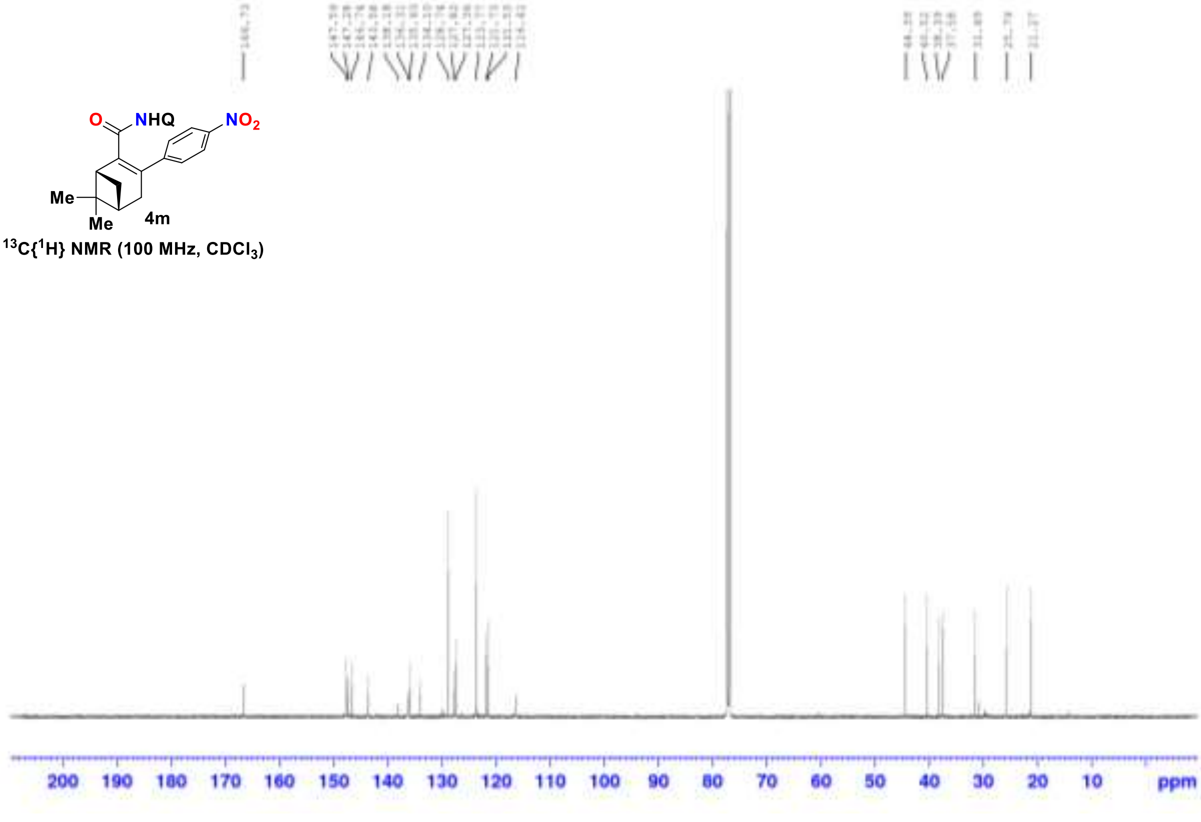
O
NHQ
NO2
Me
Me
4m
13C{1H} NMR (100 MHz, CDCl3)
200 190 180 170 160 150 140 130 120 110 100 90 80 70 60 50 40 30 20 10 ppm

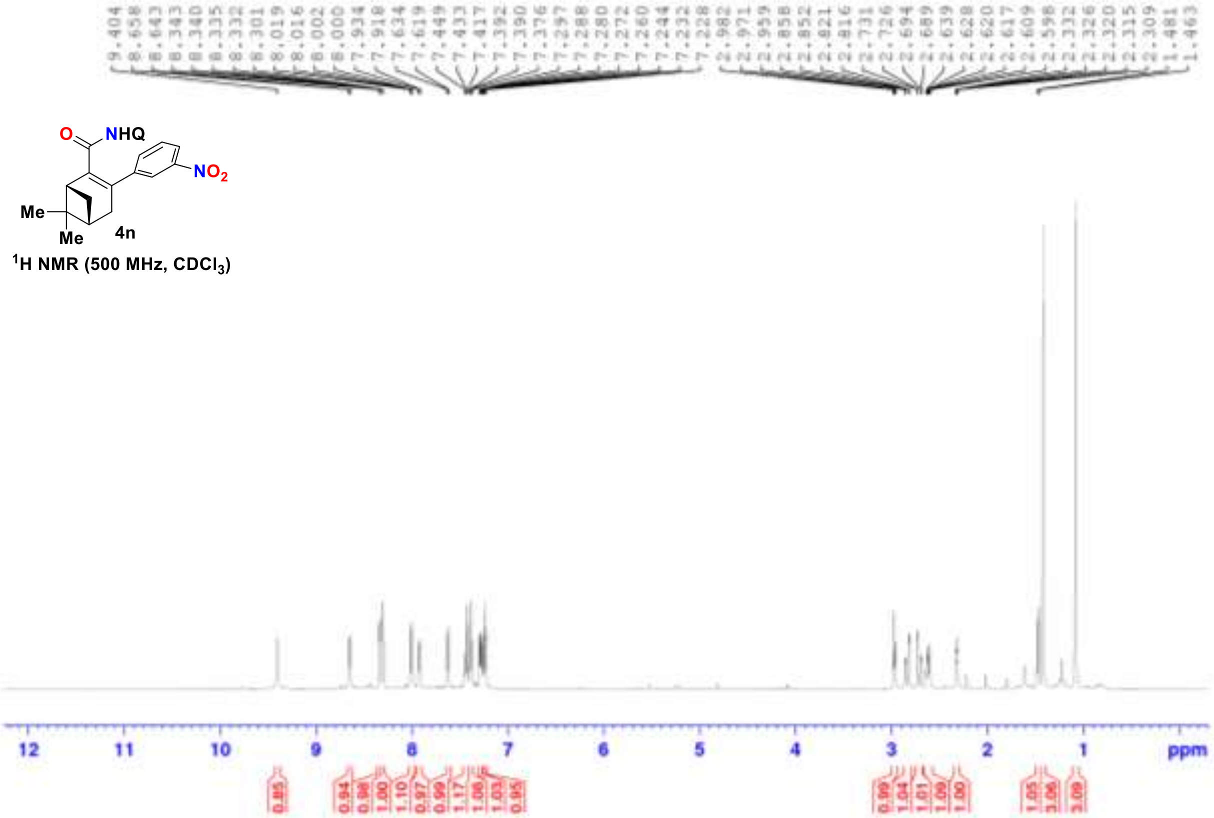

4n

^{1}H NMR (500 MHz, $CDCl_3$)

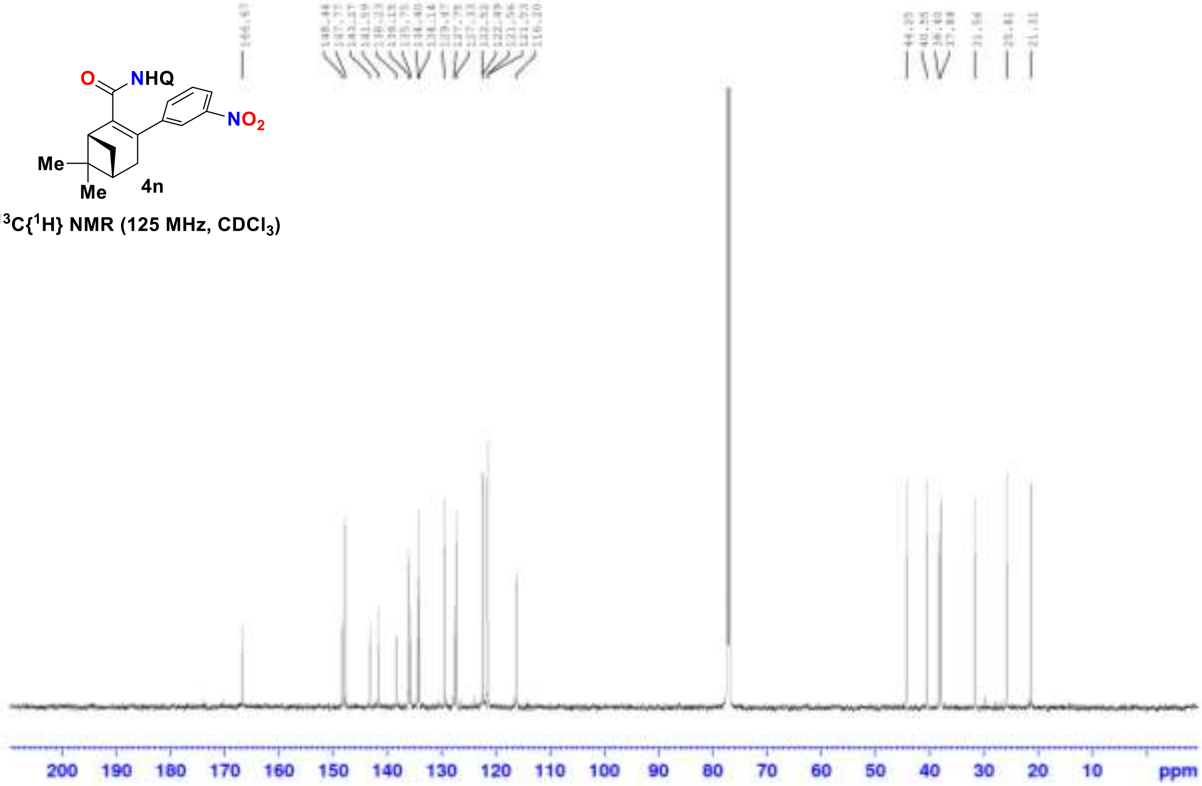

4n

$^{13}C\{^1H\}$ NMR (125 MHz, $CDCl_3$)

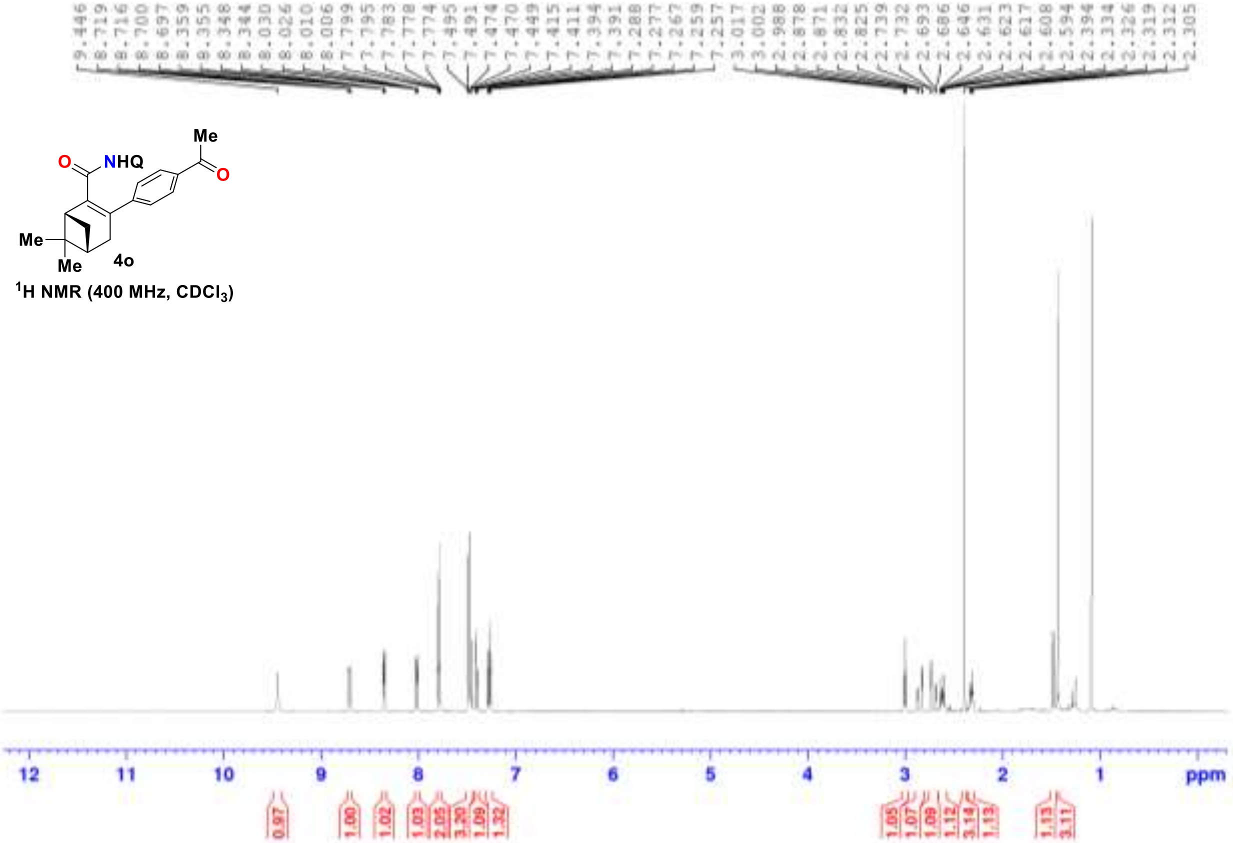
9.446 8.719 8.716 8.700 8.697 8.359 8.355 8.348 8.344 8.030 8.026 8.010 8.006 7.799 7.795 7.783 7.778 7.774 7.495 7.491 7.474 7.470 7.449 7.415 7.411 7.394 7.391 7.288 7.277 7.267 7.259 7.257 3.017 3.002 2.988 2.878 2.871 2.832 2.825 2.739 2.732 2.693 2.686 2.646 2.631 2.623 2.617 2.608 2.594 2.394 2.334 2.326 2.319 2.312 2.305
Me
O
NHQ
O
Me
Me
4o
1H NMR (400 MHz, CDCl3)
12 11 10 9 8 7 6 5 4 3 2 1 ppm
0.97 1.00 1.02 1.03 2.05 3.20 1.09 1.32
1.05 1.07 1.09 1.12 3.14 1.13 1.13 3.11

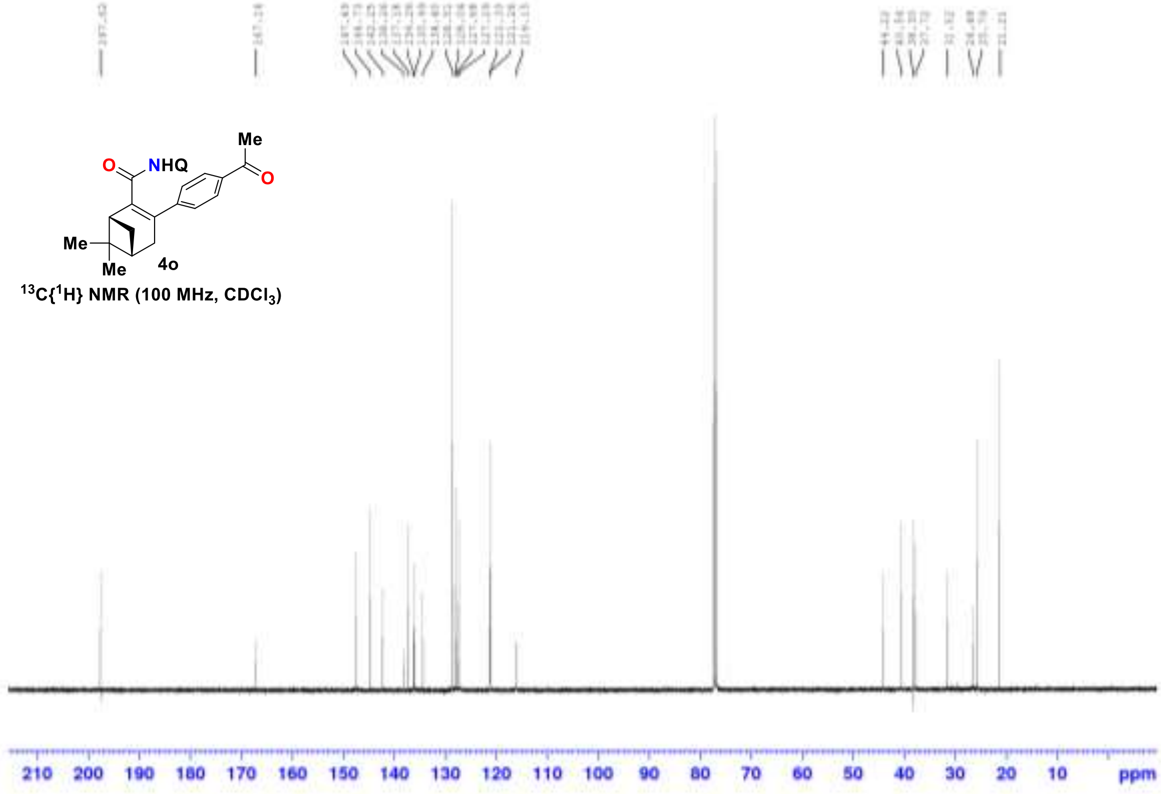
Me
O
NHQ
O
Me
Me
4o
13C{1H} NMR (100 MHz, CDCl3)
210 200 190 180 170 160 150 140 130 120 110 100 90 80 70 60 50 40 30 20 10 ppm

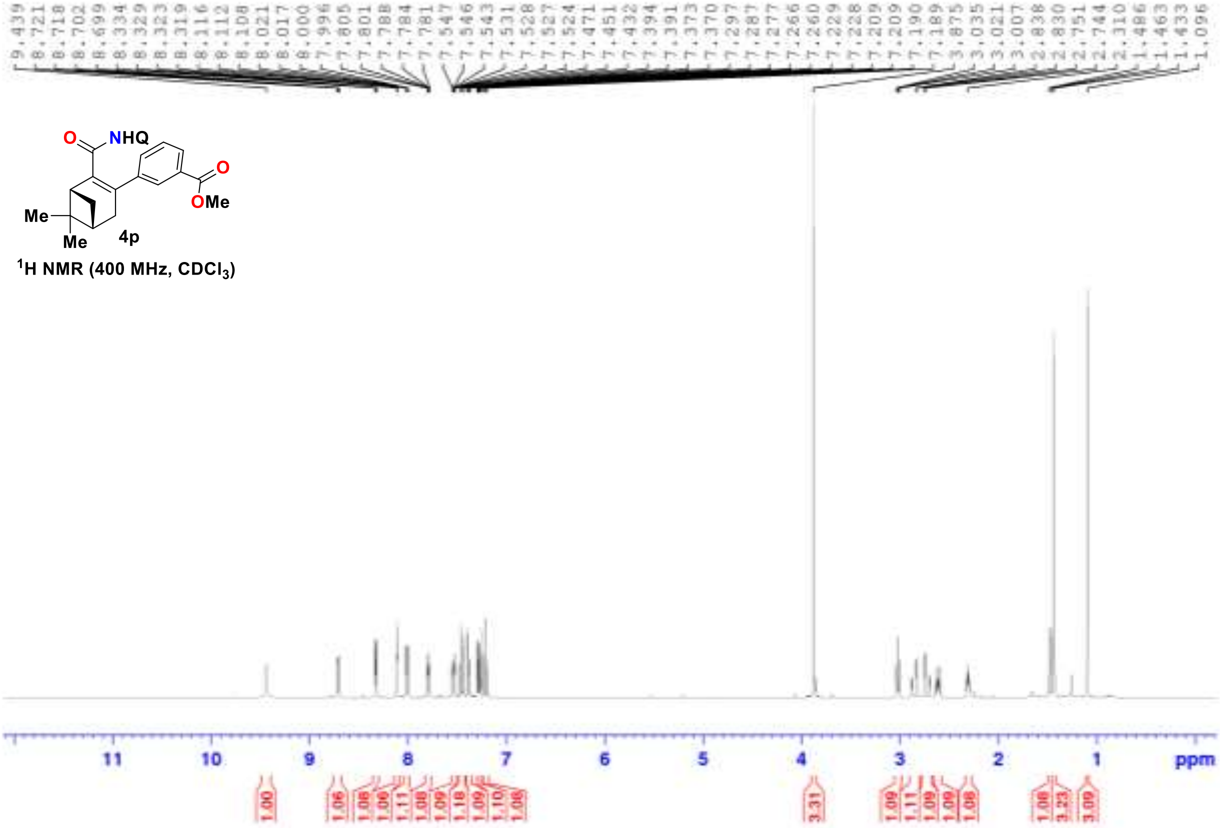
O
NHQ
O
OMe
Me
Me
4p
1H NMR (400 MHz, CDCl3)
ppm

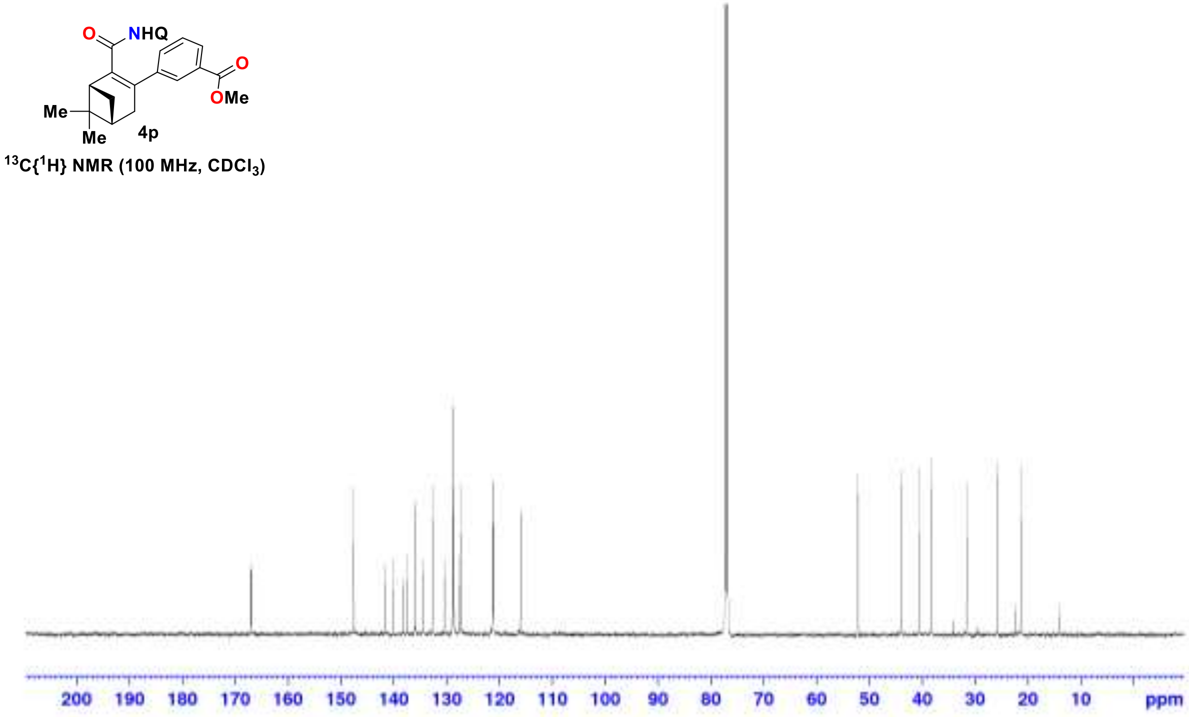
O
NHQ
O
OMe
Me
Me
4p
13C{1H} NMR (100 MHz, CDCl3)
ppm

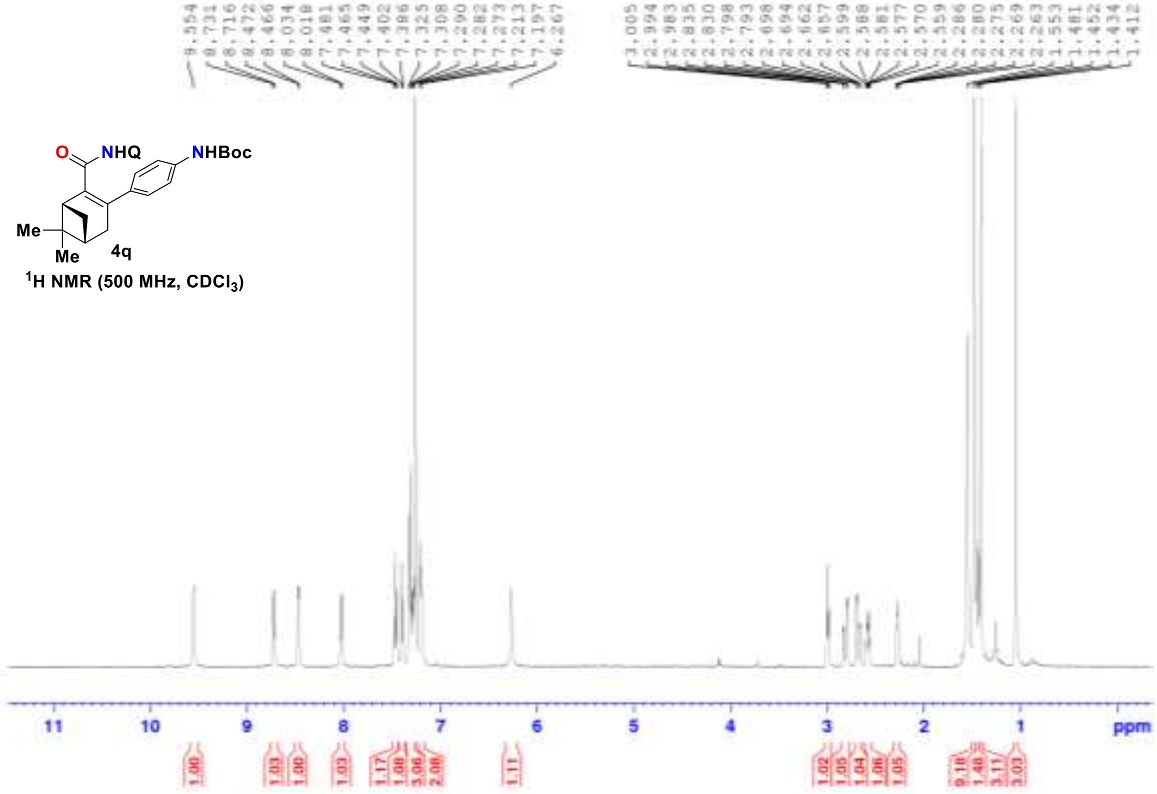

NHQ
NHBoc
O
Me
Me
4q
^{1}H NMR (500 MHz, $CDCl_3$)
ppm

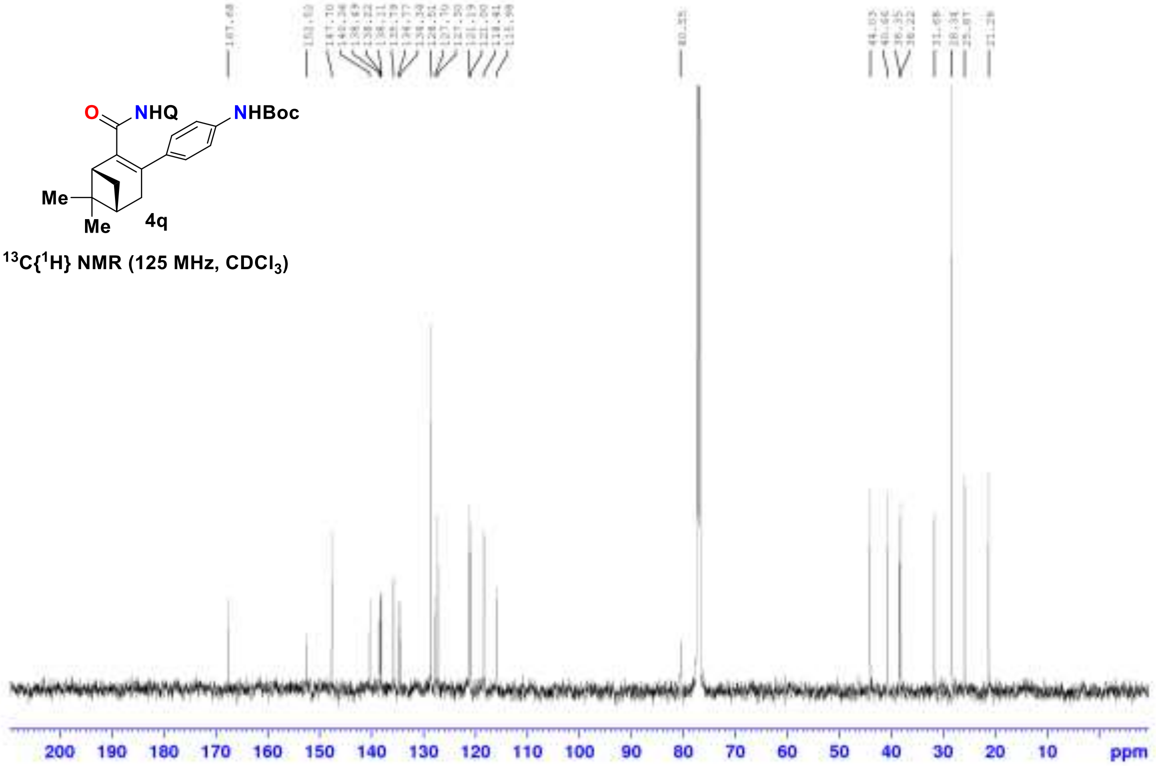

NHQ
NHBoc
O
Me
Me
4q
^{13}C{^{1}H} NMR (125 MHz, $CDCl_3$)
ppm

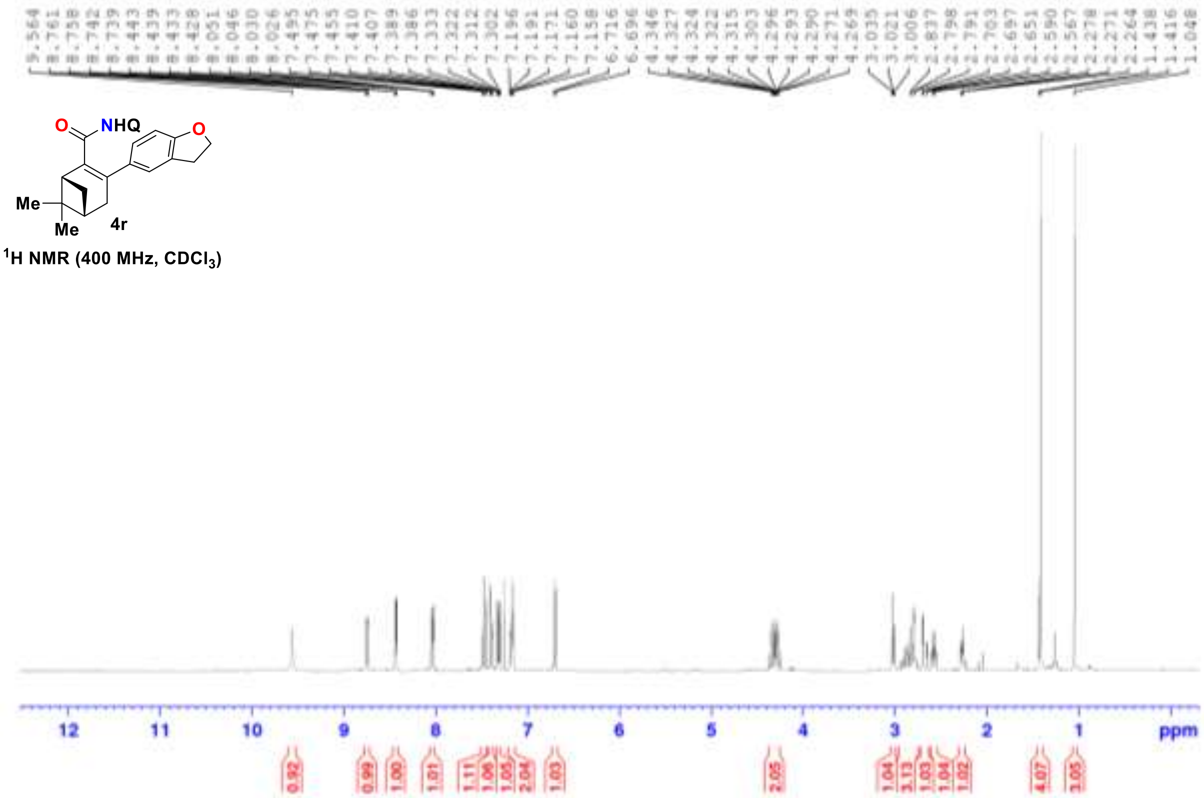
9.564 8.761 8.758 8.742 8.739 8.443 8.439 8.433 8.428 8.051 8.046 8.030 8.026 7.495 7.475 7.455 7.410 7.407 7.389 7.386 7.333 7.322 7.312 7.302 7.196 7.191 7.171 7.160 7.158 6.716 6.696 4.346 4.327 4.324 4.322 4.315 4.303 4.296 4.293 4.290 4.271 4.269 3.035 3.021 3.006 2.837 2.798 2.791 2.703 2.697 2.651 2.590 2.567 2.278 2.271 2.264 1.438 1.416 1.048
O
NHQ
O
Me
Me
4r
1H NMR (400 MHz, CDCl3)
12 11 10 9 8 7 6 5 4 3 2 1 ppm
0.92 0.99 1.00 1.01 1.11 1.08 1.05 2.04 1.03 2.05 1.04 3.13 1.03 1.04 1.02 4.07 3.05

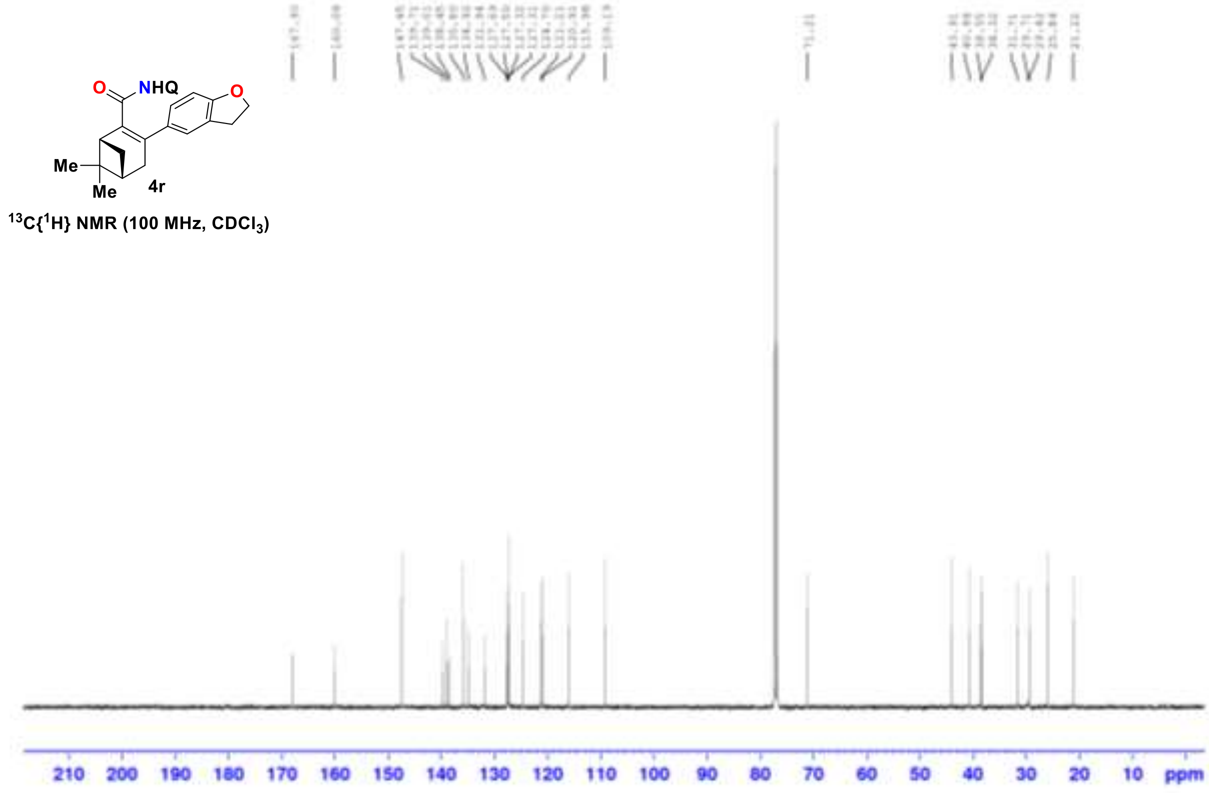
O
NHQ
O
Me
Me
4r
13C{1H} NMR (100 MHz, CDCl3)
210 200 190 180 170 160 150 140 130 120 110 100 90 80 70 60 50 40 30 20 10 ppm

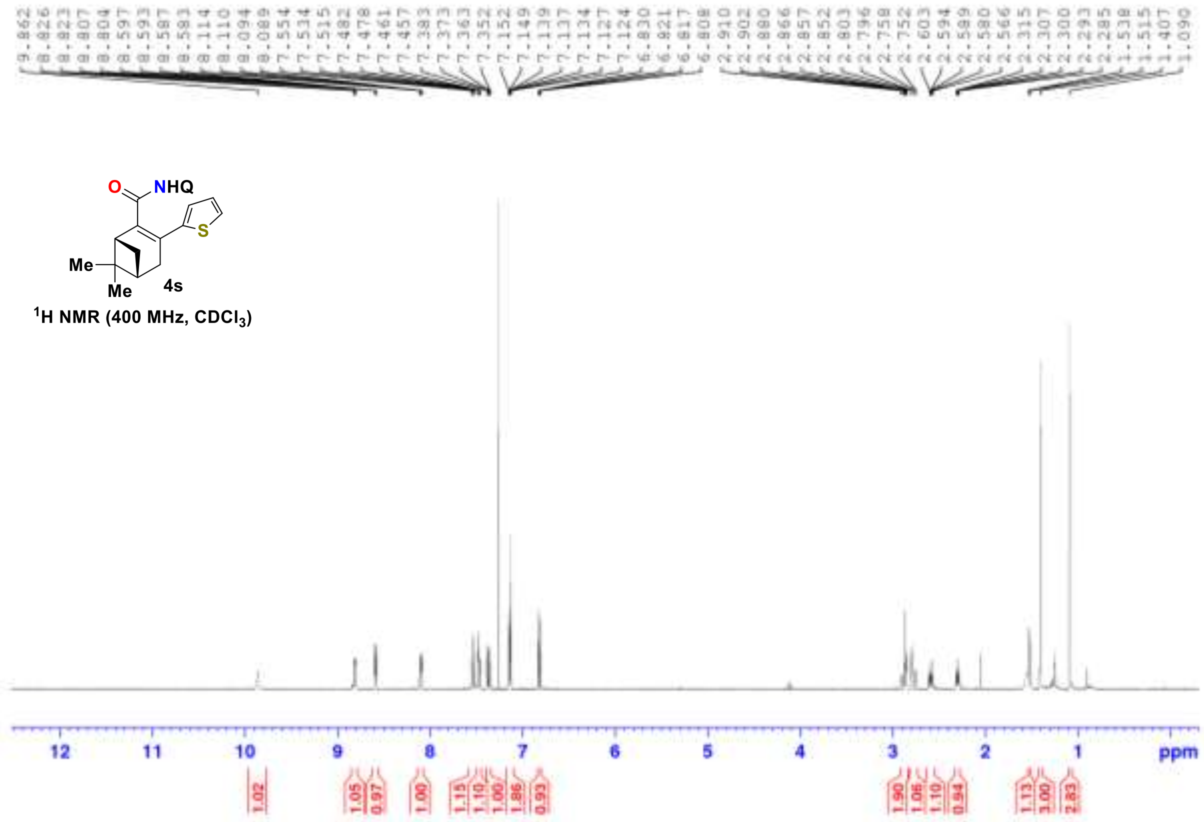
O
NHQ
S
Me
Me
4s
1H NMR (400 MHz, CDCl3)
ppm

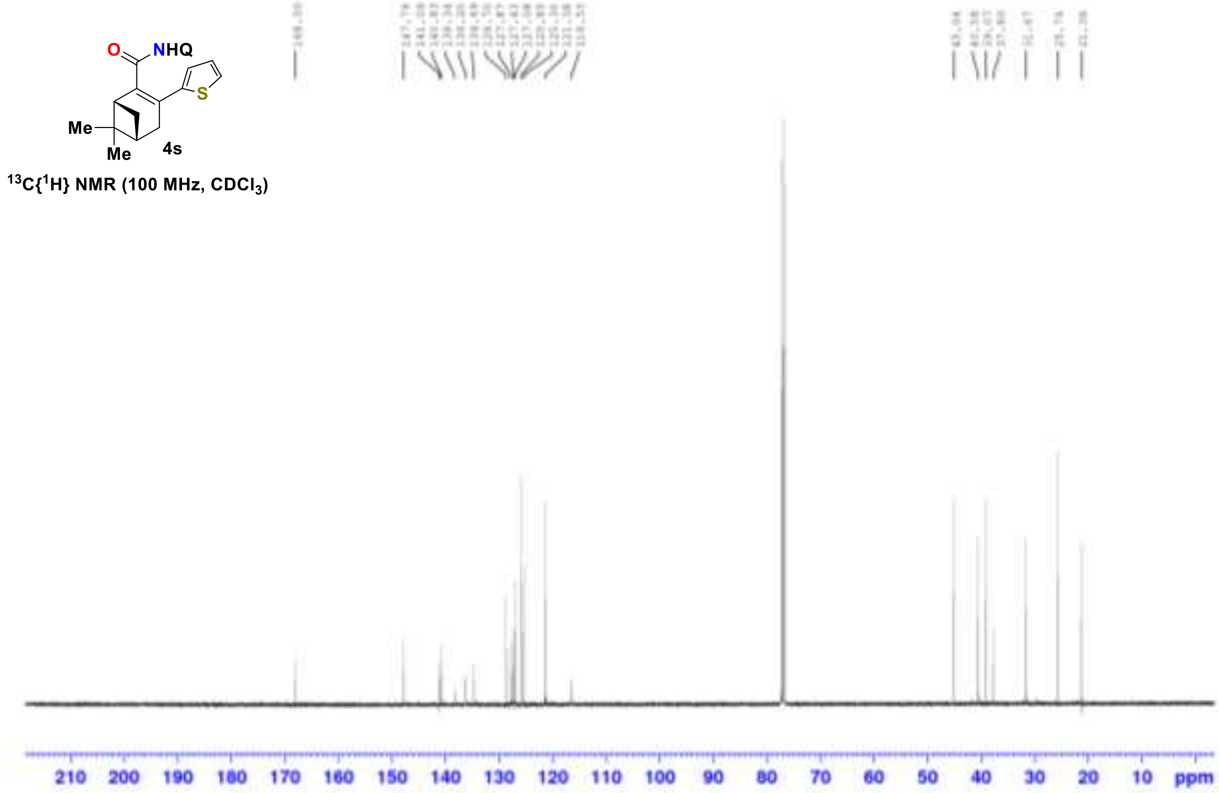
O
NHQ
S
Me
Me
4s
13C{1H} NMR (100 MHz, CDCl3)
ppm

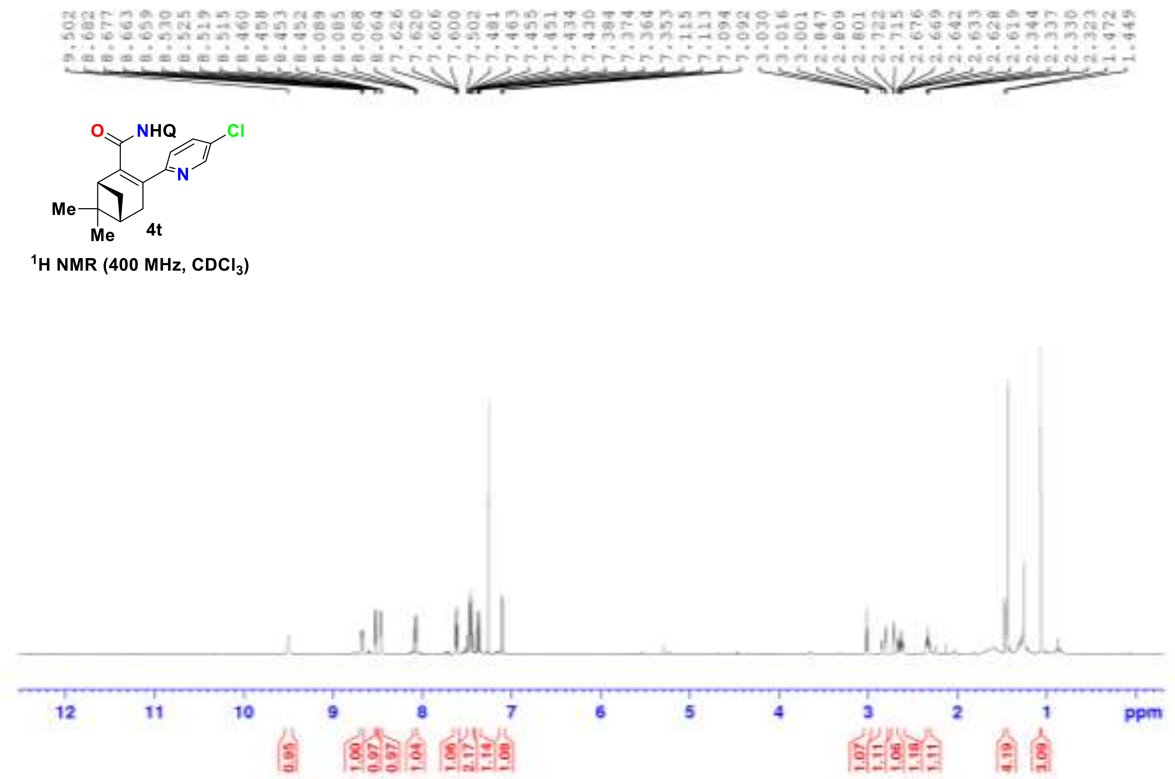
O
NHQ
Cl
N
Me
Me
4t
1H NMR (400 MHz, CDCl3)
ppm

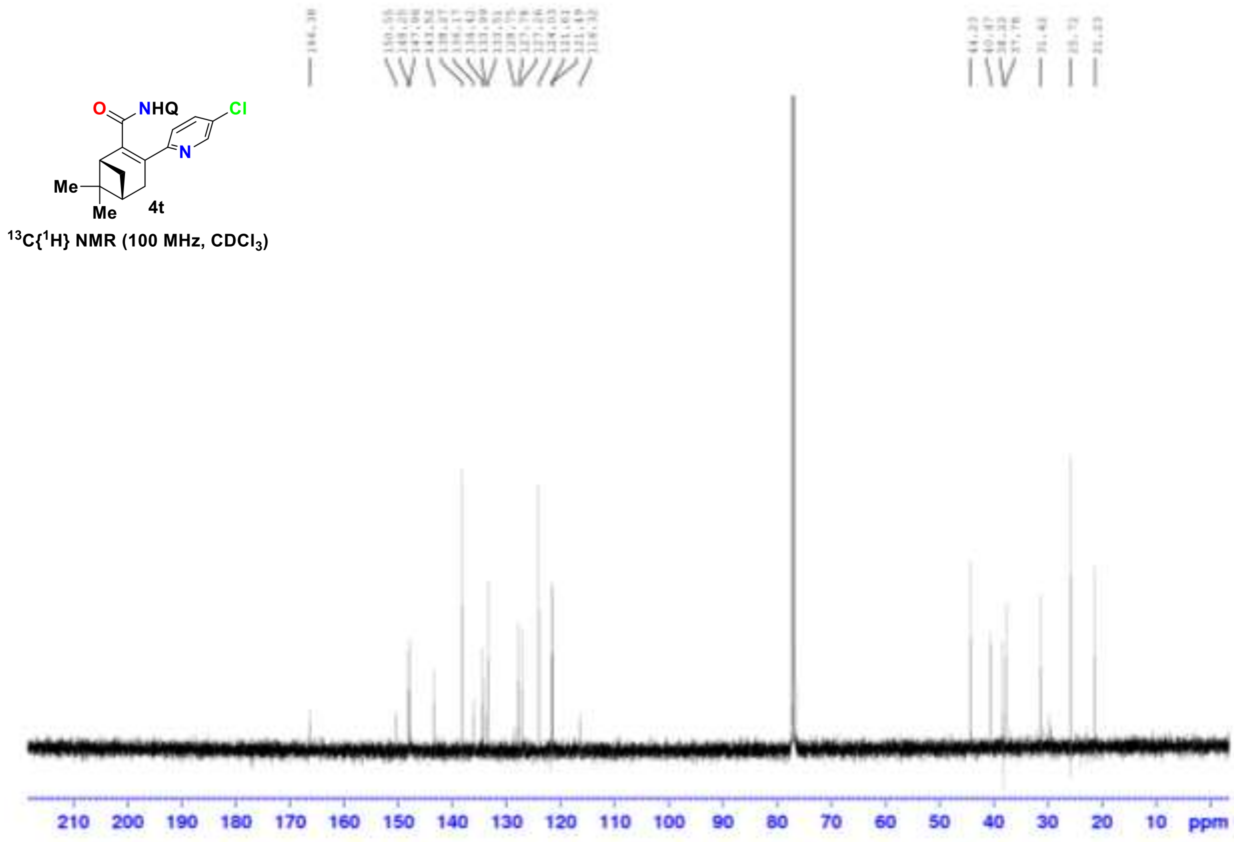
O
NHQ
Cl
N
Me
Me
4t
13C{1H} NMR (100 MHz, CDCl3)
ppm

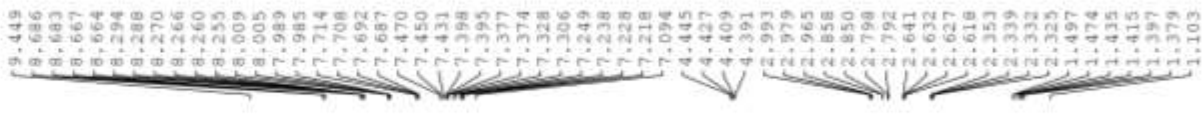

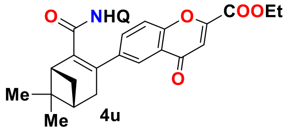

4u

^{1}H NMR (400 MHz, $CDCl_3$)

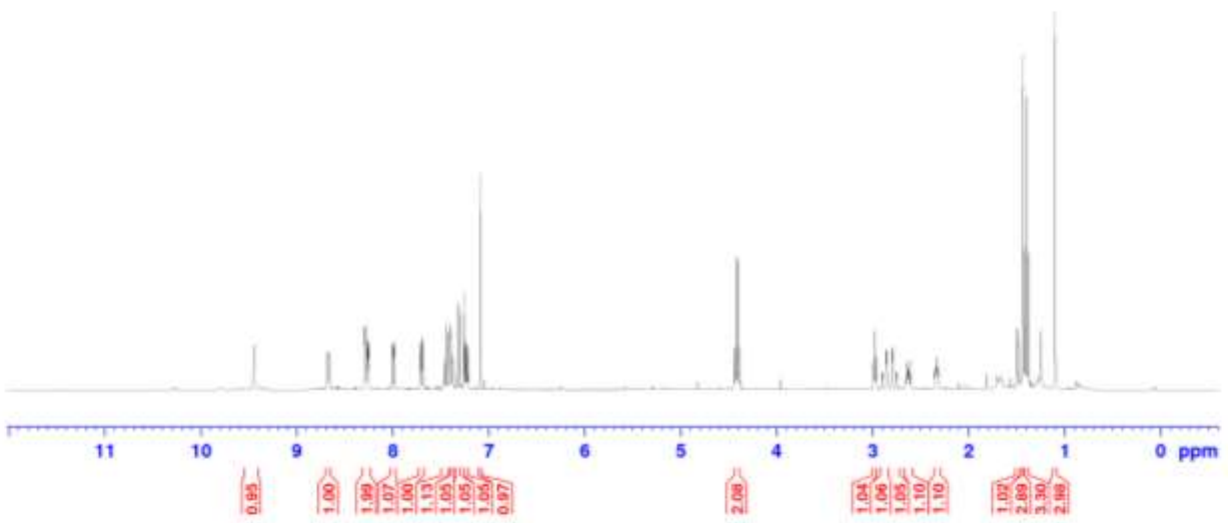

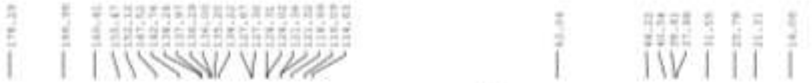

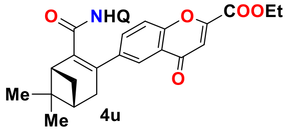

4u

^{13}C{^{1}H} NMR (100 MHz, $CDCl_3$)

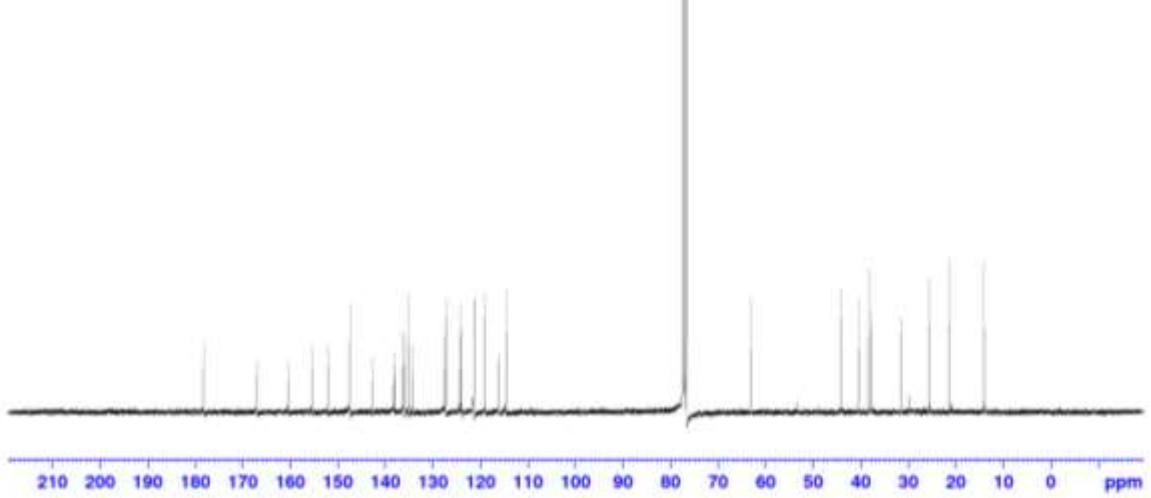

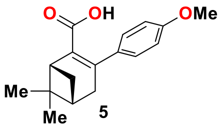

5

^{1}H NMR (400 MHz, $CDCl_3$)

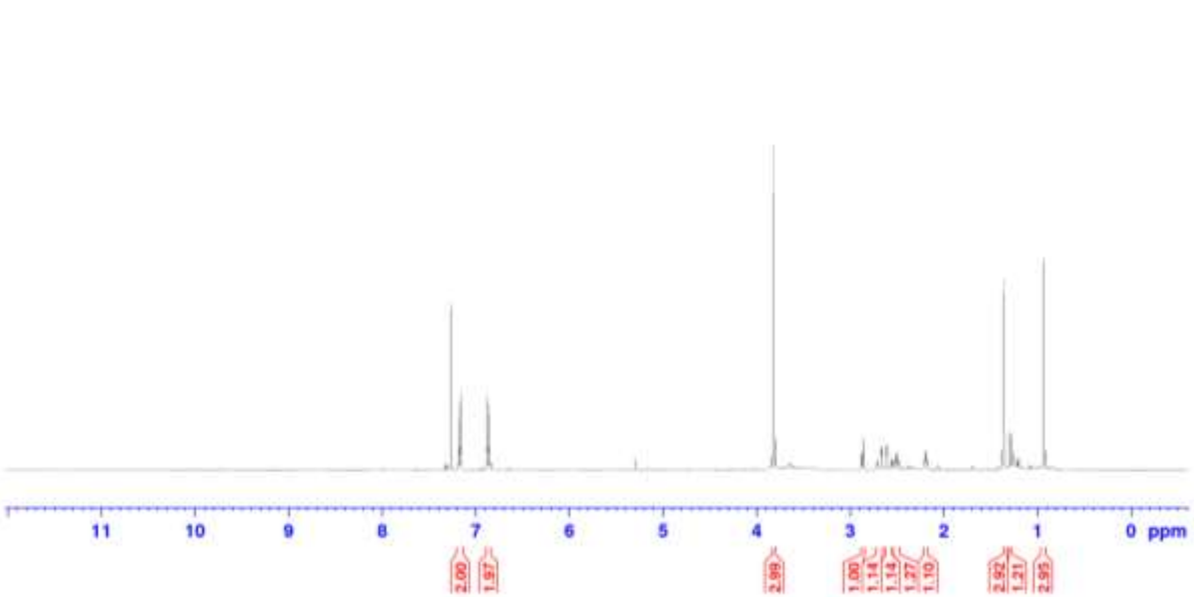

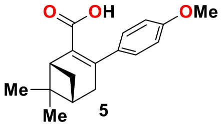

5

$^{13}C\{^{1}H\}$ NMR (100 MHz, $CDCl_3$)

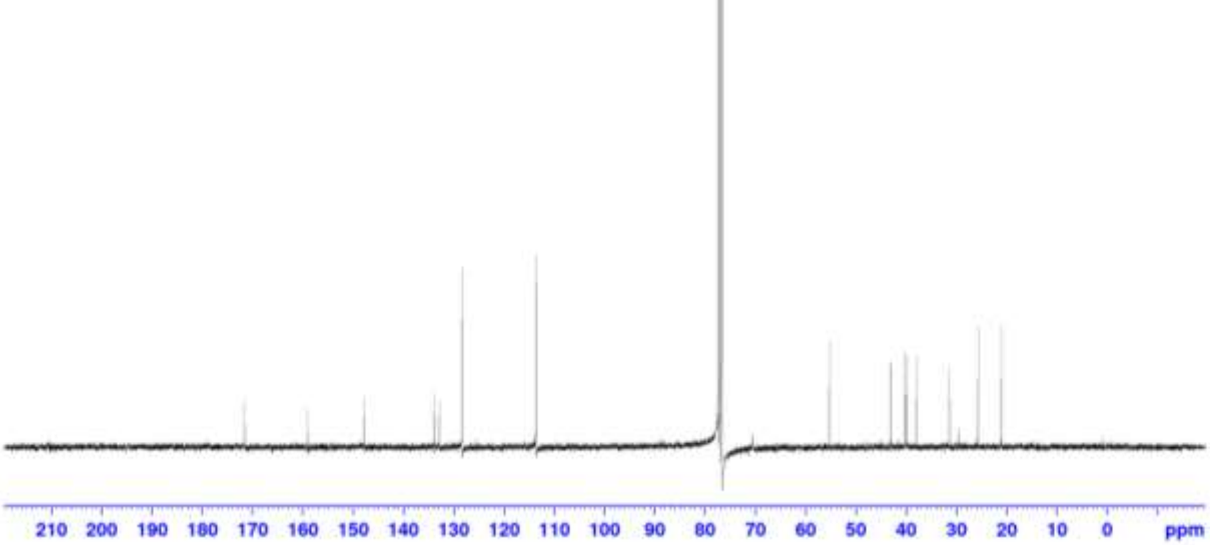

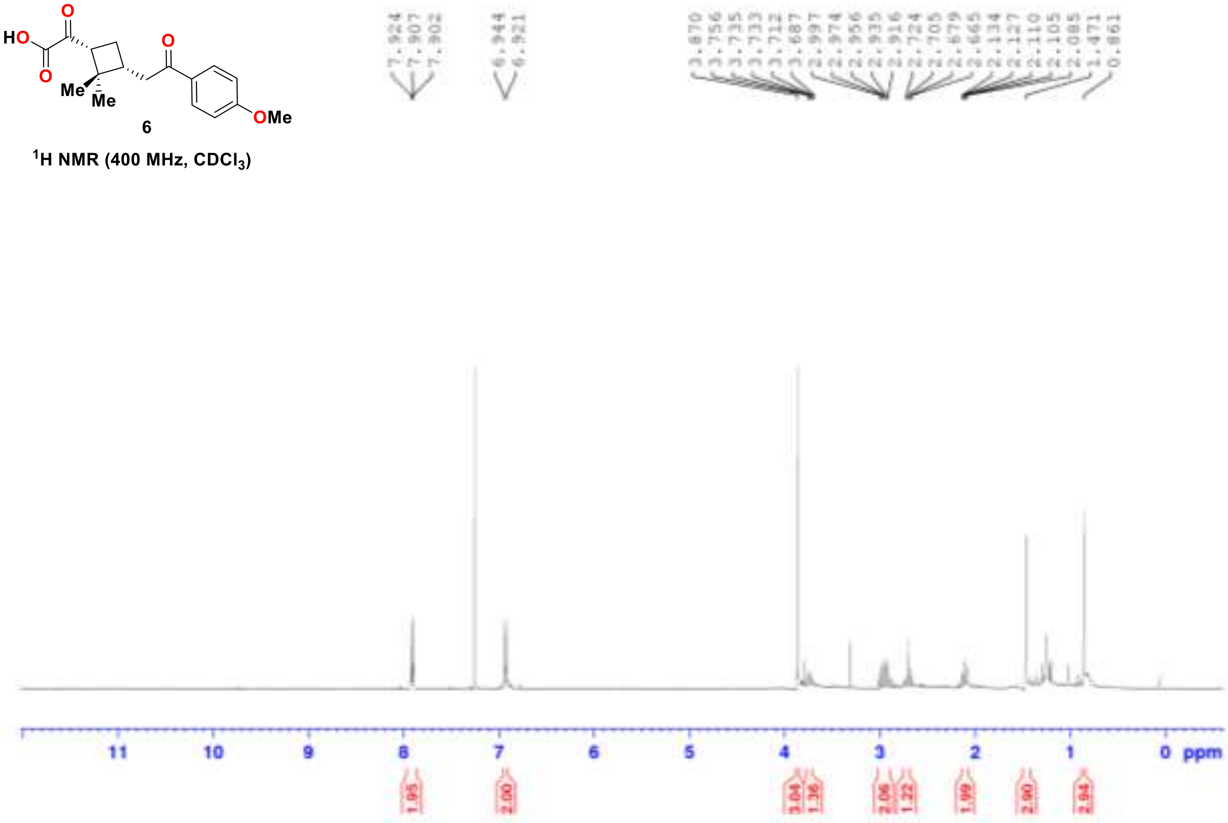
HO
O
O
O
Me
Me
OMe
6
1H NMR (400 MHz, CDCl3)
7.924
7.907
7.902
6.944
6.921
3.870
3.756
3.735
3.733
3.712
3.687
2.997
2.974
2.956
2.935
2.916
2.724
2.705
2.679
2.665
2.134
2.127
2.110
2.105
2.085
1.471
0.861
11 10 9 8 7 6 5 4 3 2 1 0 ppm
1.95
2.00
3.04
1.36
2.06
1.22
1.99
2.90
2.94

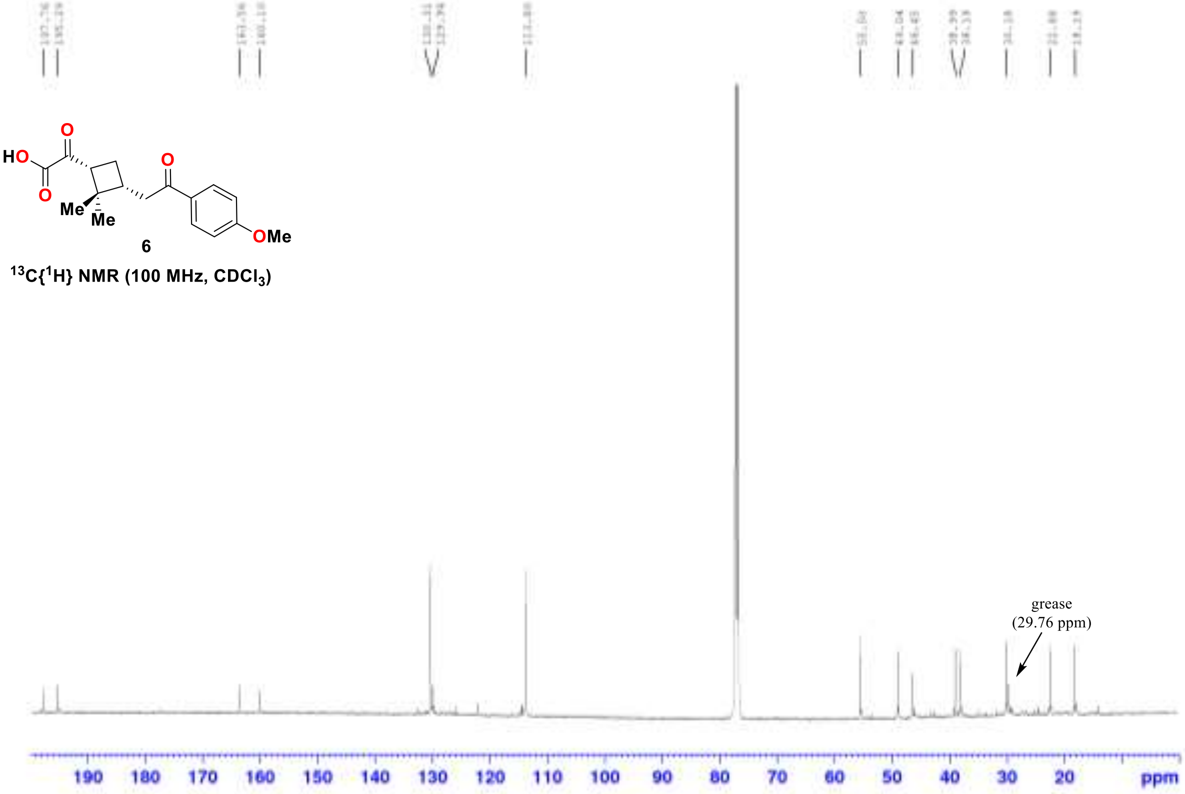
HO
O
O
O
Me
Me
OMe
6
13C{1H} NMR (100 MHz, CDCl3)
grease
(29.76 ppm)
190 180 170 160 150 140 130 120 110 100 90 80 70 60 50 40 30 20 ppm
